# Cardiovascular Performance Measurement in Water Fleas by Utilizing High-Speed Videography and ImageJ Software and Its Application for Pesticide Toxicity Assessment

**DOI:** 10.3390/ani10091587

**Published:** 2020-09-05

**Authors:** Fiorency Santoso, Viacheslav V. Krylov, Agnes L. Castillo, Ferry Saputra, Hong-Ming Chen, Hong-Thih Lai, Chung-Der Hsiao

**Affiliations:** 1 Department of Bioscience Technology, Chung Yuan Christian University, Chung-Li 320314, Taiwan; fiorency_santoso@yahoo.co.id (F.S.); ferrysaputratj@gmail.com (F.S.); 2 Master Program of Nanotechnology, Chung Yuan Christian University, Chung-Li 320314, Taiwan; 3 Papanin Institute for Biology of Inland Waters, Russian Academy of Sciences, Borok, Nekouzskii raion, Yaroslavl oblast 152742, Russia; krylovviacheslav@mail.ru; 4 Faculty of Pharmacy, The Graduate School and Research Center for the Natural and Applied Sciences, University of Santo Tomas, Manila 1008, Philippines; alcastillo@ust.edu.ph; 5 Department of Aquatic Biosciences, National Chiayi University, 300 Syuefu Rd. Chiayi 60004, Taiwan; s1070474@mail.ncyu.edu.tw; 6 Department of Chemistry, Chung Yuan Christian University, Chung-Li 320314, Taiwan; 7 Center for Nanotechnology, Chung Yuan Christian University, Chung-Li 320314, Taiwan

**Keywords:** water flea, *Daphnia*, cardiovascular performance, ImageJ

## Abstract

**Simple Summary:**

With the advantages of easy culture, body transparency, and high sensitivity to chemical pollution, water fleas have been recognized as a good model for ecotoxicity studies. In this paper, we established ImageJ-based methods to measure cardiovascular performance by evaluating the heart rate and blood flow velocity in three water fleas for the first time. Among the three water fleas, *Daphnia magna* was identified as having the most robust heartbeat and blood flow rate and is therefore suitable for ecotoxicity assessment. Many important parameters like heart rate, blood flow rate, stroke volume, ejection fraction, fractional shortening, cardiac output heartbeat regularity can be extracted from videotaping and mathematical calculation. In utilizing those physiological parameters, the potential impacts of ambient water temperature and pesticide pollution on water fleas can be precisely measured.

**Abstract:**

Water fleas are a good model for ecotoxicity studies, and were proposed for this purpose by the United States Environmental Protection Agency, due to their easy culture, body transparency, and high sensitivity to chemical pollution. Cardiovascular function parameters are usually used as an indicator of toxicity evaluation. However, due to the nature of the heart and blood flow, and the speed of the heartbeat, it is difficult to perform precise heartbeat and blood flow measurements with a low level of bias. In addition, the other cardiovascular parameters, including stroke volume, cardiac output, fractional shortening, and ejection fraction, have seldom been carefully addressed in previous studies. In this paper, high-speed videography and ImageJ-based methods were adopted to analyze cardiovascular function in water fleas. The heartbeat and blood flow for three water flea species, *Daphnia magna*, *Daphnia silimis*, and *Moina* sp., were captured by high-speed videography and analyzed using open-source ImageJ software. We found the heartbeat is species-dependent but not size-dependent in water fleas. Among the three water fleas tested, *D. magna* was identified as having the most robust heartbeat and blood flow rate, and is therefore suitable for the ecotoxicity test. Moreover, by calculating the diameter of the heart, we succeeded in measuring other cardiovascular parameters. *D. magna* were challenged with temperature changes and a pesticide (imidacloprid) to analyze variations in its cardiovascular function. We found that the heartbeat of *D. magna* was temperature-dependent, since the heartbeat was increasing with temperature. A similar result was shown in the cardiac output parameter. We also observed that the heartbeat, cardiac output, and heartbeat regularity are significantly reduced when exposed to imidacloprid at a low dose of 1 ppb (parts per billion). The blood flow rate, stroke volume, ejection fraction, and fractional shortening, on the contrary, did not display significant changes. In conclusion, in this study, we report a simple, highly accurate, and cost-effective method to perform physiological and toxicological assessments in water fleas.

## 1. Introduction

Freshwater is a vital component of the global ecosystem. Currently, freshwater pollution is becoming a major threat to the world, not only because it poses a risk to the environment, but also because it can affect human health. Heavy metals or agricultural chemicals such as pesticides, herbicides, and fertilizers are harmful at certain levels of exposure due to their accumulation in relatively high concentrations in the food chains of aquatic ecosystems. These toxic substances may have adverse effects on the development, growth, reproduction, and behavior of freshwater organisms, and can even cause death [1]. Small cladocerans, commonly called water fleas, are among the most preferred animals for aquatic toxicity testing due to their short life span, as well as the fact that they can easily be cultured and are rapidly affected by chemicals [2]. These invertebrate species are not only the major component of the zooplankton diet of fish, but also function as the first level consumers of freshwater food chains [3]. Since sensitivity varies according to the toxic agents and environmental conditions, the use of more than one cladocerans species may be helpful in eco-toxicological assays [4]. 

Water fleas have transparent bodies and relatively large hearts, facilitating direct observation under a microscope [5]. This allows for the study of the effects of toxicants on their cardiovascular systems. In water flea species, blood is not enclosed in vessels but instead is pumped into a cavity called a hemocoel, and the blood itself is called hemolymph, as the blood mixes with the interstitial fluid. As the heart beats and the animal moves, the hemolymph circulates among the body’s organs and then re-enters the heart through openings called ostia. This movement allows for the exchange of gas and nutrient in the body of *Daphnia* species [6].

*Daphnia magna* is a commonly used species for the testing of acute and chronic toxicity [7]. Its high sensitivity allows the use of an acute toxicity test, with *D. magna* used as a prescreening toxicity assay before the testing in rats, to reduce the number of mammals and for cost and time effectiveness [8]. *Daphnia similis* can also be used as a test organism, as an alternative species to *D. magna*, due to its different geological distribution, smaller size, and higher breeding speed [9]. Another species, *Moina* sp. is also an ideal animal for ecological relevance due to its wide occurrence, short life cycle, genetic uniformity, relative ease of culture in the laboratory, and sensitivity to toxicants [10]. Furthermore, a previous study reported that *Daphnia pulex* is the first crustacean to have its genome sequenced. The genome of *Daphnia* shows the strongest homology with the human genome [11]. 

Cardiovascular disease is one of the diseases which has become a major cause of death in humans. It can lead to disruption of blood flow to the heart, damage to cardiac muscle, heart rate irregularity, and death. *Daphnia* has also been proven as an emerging model in medical science. It can display varying arrhythmia on exposure to pro-arrhythmia chemicals and responds to many cardio-active drugs known to affect human heart function [12]. Moreover, Daphnia’s heart has an intrinsic ability to contract, considered a myogenic heart, representing a model system used to understand the myogenic hearts of vertebrates [12]. Therefore, this enables the application of *Daphnia* as a surrogate model to investigate the effect of toxicants on its cardiovascular function.

Acute toxicity can be measured with cladocerans in a relatively short period of 48 h [13]. The more sensitive test for chronic toxicity with daphnids has a 21-day duration, is time-consuming, and requires complex manipulation. A few studies have attempted to shorten the test period to 14 days [14], to use a single brood [15], or to use shorter test protocols [16]. However, these approaches are not widely used. Evaluating the cladoceran juvenile growth rate [17] or the rates of parthenogenetic egg development in vitro [18] was suggested as a toxicological approach for reducing the exposure period. However, the performing of such an assay is relatively laborious and requires relevant experience. Here we suggest a rapid, cost-effective, and sensitive method for assessing toxicity based on heart rate and blood flow in these freshwater cladocerans.

Traditionally, water fleas are immersed in test solutions for specific times, and the heartbeat is observed under a microscope. Some studies counted the heart rates in invertebrate animals with the aid of a stopwatch and the use of a pen and paper to tap the beats manually [3,19,20,21]. However, with this manual method, it can be challenging to gather accurate and reliable data, due to the animal’s extremely fast heartbeat [5]. On the other hand, some studies have used a complex apparatus and software analysis to study the cardiovascular system in water fleas, although these methods may be difficult for other scientists to follow [22,23,24]. Furthermore, the use of an inverted microscope was applied to study the effect of β-adrenergic antagonists propranolol and metoprolol on the heartbeat of *D. magna* [25] as well as the effect of hypoxia-tolerance, a decade ago [26,27]. In the current study, we describe a detailed protocol for a simple and cost-effective method to study the cardiovascular function parameters, e.g., heart rate, blood flow velocity, stroke volume, and cardiac output, in three water flea species—*D. magna*, *D. similis*, and *Moina* sp. An inverted microscope mounted with a high- speed charge-coupled device (CCD) camera was used to capture the image, whereas the public and user-friendly Java-based image processing program ImageJ was used to quantify the data. In addition, the effect of temperature changes and pesticide exposure on the cardiovascular function parameters were tested in *D. magna* in detail.

## 2. Materials and Methods 

### 2.1. Water Flea Maintenance and Sample Preparation

*D. magna*, *D. similis*, and *Moina* sp. were obtained from the National Chiayi University Freshwater Resource Center. The water fleas were maintained in 12 L of culture medium under a light–dark period of 14 h: 10 h with a temperature of 20 °C ± 2 °C and pH 6.7 and a water conductivity between 300 and 1500 µS. The animals were fed by either green algae or beaker yeast suspension two times a day, and the cultured water was exchanged every two days (≥80%). The maintenance culture and the following tests were performed in the Chung Yuan Christian University (CYCU), and all followed the guidelines for laboratory animals of CYCU. The protocol was approved by the Animal Ethics Committee of Chung Yuan Christian University, with approval number 107029 (8 January 2019). Three different water flea species with body areas ranging between 3 mm^2^ to 6 mm^2^ were selected to analyze the body size, heartbeat and blood flow velocity. Fifty water fleas for each species were used for the experiment. Selected water fleas were then moved to a 6-cm plastic Petri dish. Excessive water was removed before 3% methylcellulose was used to mount the water fleas to reduce movement. Subsequently, water fleas immobilized on Petri dishes were utilized for the videography procedure.

### 2.2. The Effect of Temperature Change and Pesticide Exposure on Cardiovascular Performance

**Figure A1:**
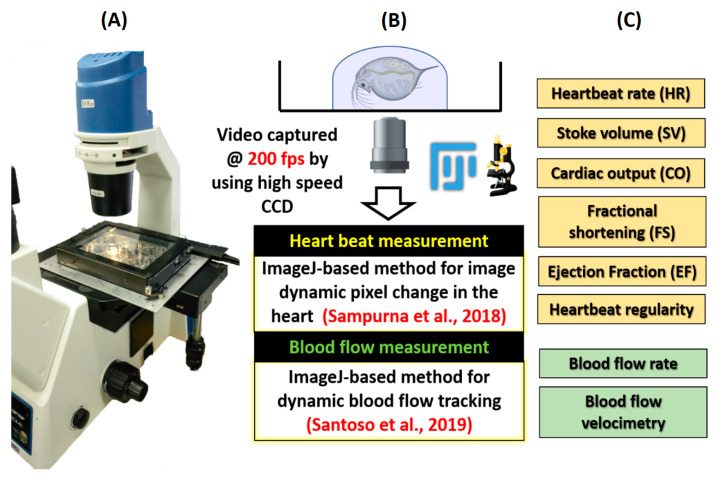
Schematic diagram showing the instrument setting (**A**), the principle used to conduct heartbeat and blood flow calculation (**B**), and some physiological endpoints that can be measured (**C**) in water fleas.

In this study, *D. magna* was taken as the representative animal model. To study the temperature effect, a temperature chamber (U 140A, BLAST, Japan) connected to a heater was used to maintain the temperature. This chamber was placed onto the holding stage of an inverted microscope (Appendix A, Figure A1). An ice cube was put into the chamber to actualize cold temperature, and the heater was then used to increase the temperature. Video recording was done every 5 °C, starting from 15 °C to 35 °C. One-minute acclimatization was performed on each temperature before recording the video image. In this study, twenty *D. magna* were used. On the other hand, imidacloprid (Aladdin, Shanghai, China) was chosen to study the effect of pesticide in *D. magna*. Imidacloprid at 0.1-ppb, 1-ppb, 10-ppb, and 100-ppb concentrations were applied to the animals for 24 h in a 9-cm petri dish with around 50 mL volume. After 24 h, each water flea was individually transferred to a 35-mm plastic Petri dish and heart and blood flow images were recorded. Thirty *D. magna* were used for each concentration to study cardiovascular performance.

### 2.3. High-Speed Videography

In analyzing the correlation of body size with the heartbeat and stroke volume, a dissecting microscope equipped with a digital charge-coupled device (CCD) camera was used to capture the images of water fleas for body size estimation. Subsequently, water fleas mounted with 3% methylcellulose on Petri dishes were transferred to record the heartbeat and blood flow using an inverted microscope (ICX41, Sunny Optical Technology, China) equipped with a high-speed digital charged coupling device (CCD) (Zgenebio, Taipei, Taiwan). HiBestViewer (AZ Instrument, Taiwan) software was used to conduct high-speed video recording at 200 fps (frames per second) for 10 s. This study was performed according to our previously established method [28].

### 2.4. Cardiovascular Parameter Calculation

The Fiji distribution of ImageJ (https://imagej.net/Fiji/Downloads) was used as a major platform to conduct the experiment. The TrackMate plug-in (https://imagej.net/TrackMate) with default settings was used to track the blood cell movement. Combining the Gaussian filter before applying the Laplacian filter, called Laplacian of Gaussian (LoG) segmentation, was the algorithm used by the TrackMate plug-in to track single blood cell motion. Blood cell velocity can be calculated according to the X and Y coordinates. On the other hand, the heartbeat can be measured from dynamic pixel change data obtained from the Time Series Analyzer plug-in (https://imagej.nih.gov/ij/plugins/time-series.html). The data were then processed using Origin 9.1 software (Originlab Corporation, Northampton, MA, USA) and Microsoft Excel (Microsoft 2016 version, Seattle, WA, USA). The heart rate was defined as beats per minute (bpm) and was obtained by dividing one minute (60 s) by the time interval. The heartbeat time interval was calculated by subtracting two consecutive time points [28,29] (detailed method can be found in the File S1).

Other cardiovascular parameters, including ejection fraction, fractional shortening, stroke volume, and cardiac output, can also be obtained by analyzing heart images. The heart’s diameter at the diastolic stage (heart relaxation) and systolic stage (heart contraction) was manually measured using ImageJ. Fractional shortening (FS) is calculated by measuring the percentage change in the short diameter of the heart during the diastole and systole stages (Equation (1)). D_SD_ refers to the length of the short heart diameter in the diastolic stage, whereas D_SS_ represents the length of the short heart diameter in the systolic stage.

(1) $$\mathrm{FS}=\;\frac{D_{\mathrm{SD}}\left(\mathrm{diastolic}\;\mathrm{stage}\right)\;-{\;D}_{\mathrm{SS}}\;\left(\mathrm{systolic}\;\mathrm{stage}\right)}{D_{\mathrm{SS}}\left(\mathrm{systolic}\;\mathrm{stage}\right)}\;\times \;100\%$$

Stroke volume (SV), the volume ejected in each heartbeat, was calculated by subtracting the heart’s end-diastolic volume (EDV) and end-systolic volume (ESV) (Equation (2)).

SV = EDV − ESV(2)

*D. magna* was considered to have a heart with an ellipsoid shape. Therefore, the heart volume can be measured using Equation (3), where D_L_ refers to the length of the long diagonal axis and D_S_ refers to the length of the short diagonal axis [30].

(3) $$\mathrm{Volume}\;=\;\frac{1}{6}\times D_{L}\times D_{S^{2}}$$

Stroke volume is also in cooperation with ejection fraction (EF). EF is the percentage change in end-diastolic volume and was calculated by Equation (4).

(4) $$\frac{\mathrm{SV}}{\mathrm{EDV}}\;\times \;100\%$$

Cardiac output (CO) is the amount of blood the heart pumps in 1 min. It is determined by multiplying the heart rate by the stroke volume (Equation (5)).

CO = SV × Heart Rate(5)

The data were then was processed using Origin 9.1 software (Originlab Corporation, Northampton, MA, USA) and Microsoft Excel (Microsoft 2016 version, Seattle, WA, USA). Heartbeat frequency was calculated by a short-time Fourier transform (STFT) and Poincaré plot in Origin 9.1 software with default settings.

### 2.5. Statistics

The statistical and graphic analysis was operated by GraphPad Prism 8 software (GraphPad Software, Inc., version 8.00, La Jolla, CA, USA). Data were presented as mean ± SEM, and a *t*-test (with parametric assay) and one-way ANOVA were used to calculate the significance. The difference between two means was significant when * *p* < 0.05, ** *p* < 0.005, *** *p* < 0.001, and **** *p* < 0.0001. All the experiments were carried out based on our previously published protocols [28,29].

## 3. Results

### 3.1. Overview of Instrument Settings and Algorithms Used to Conduct the Experiment

The simple cardiac structure and large body size of the water flea make it an ideal model for toxicological or pharmacological assessment. For heartbeat and blood flow measurements, we first mounted water fleas with methylcellulose to immobilize them in a 35-mm Petri dish. The heartbeat and blood flow images were captured by using a high-speed CCD mounted onto an inverted microscope. After video capturing, we followed our previously published ImageJ-based protocol for heartbeat measurement in zebrafish [29]. The fundamental principle of this ImageJ-based method is monitoring the dynamic pixel change in the heart chamber. To avoid the loss of image details for faster heart beat and blood flow, we set the image capturing speed at 200 fps (frames per second). The measurement of blood flow velocity was carried out according to published protocols reported by Santoso et al. [28]. Another basic principle of this ImageJ-based method is monitoring the dynamic image changes of blood flow above the daphnid’s egg chamber. Furthermore, the images of the heart were used to calculate the stroke volume and cardiac output. The overall schematic studies and the detailed operation method are attached as Appendix A Figure A1 and File S1. 

### 3.2. The Illustration of Water Flea Images and Calculation of Cardiovascular Function

**Figure 1:**
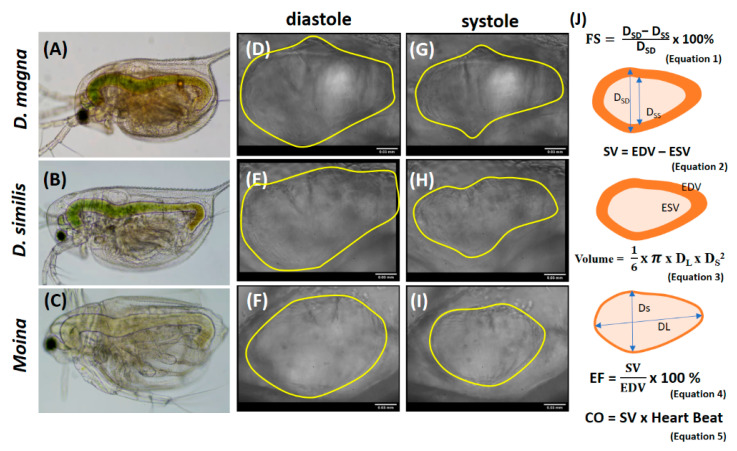
Comparison of the body images between three different water flea species: *Daphnia magna*, *Daphnia similis*, and *Moina sp*, respectively (**A**–**C**). Heart images of *D. magna*, *D. similis*, and *Moina* sp., respectively, in the diastole stage (**D**–**F**) and systole stage (**G**–**I**). (**J**) The calculation of cardiovascular parameter (fractional shortening (FS), stroke volume (SV), ejection fraction (EF), and cardiac output (CO) in water fleas. Ds_D_: heart chamber diameter in the short axis during systolic phase; Dss: heart chamber diameter in the short axis during diastolic phase; EDV: end-diastolic volume of the heart; ESV: end-systolic volume of the heart. The scale bar in (**D**), (**G**), (**E**), (**H**), (**F**), and (**I**) is 0.03 mm.

For this study, three common water flea species—*D. magna*, *D. similis,* and *Moina* sp.—were tested. The bodies of three different water fleas were observed (Figure 1A–C), as well as their hearts in either the diastole (Figure 1D–F) or the systole stage (Figure 1G–I). Figure 1J shows the image used to calculate the cardiovascular performance. Fractional shortening (FS) is the fraction of any diastolic dimension which is lost in the systole stage. This can be calculated by determining the percentage change in the end-diastolic diameter that occurs by the end of systole (Equation (1)) [31]. Stroke volume (SV), the volume ejected in each heartbeat, was calculated by subtracting the heart’s end-diastolic volume (EDV) and end-systolic volume (ESV) (Equation (2)) [32]. *D. magna* was considered to have a heart with an ellipsoid shape. Therefore, heart volume can be measured using Equation (3) [30]. Ejection fraction (EF) is the percentage change in end-diastolic volume. Ejection fraction, in cooperation with stroke volume, are two parameters that are commonly measured to evaluate the cardiovascular performance. Therefore, EF can be measured by dividing EDV with SV (Equation (4)) [32]. Cardiac output is the amount of blood the heart pumps in 1 min. It is determined by multiplying the heartbeat by the stroke volume (Equation (5)) [33].

### 3.3. Correlation of Body Size and Cardiovascular Performance in Daphnids

**Figure 2:**
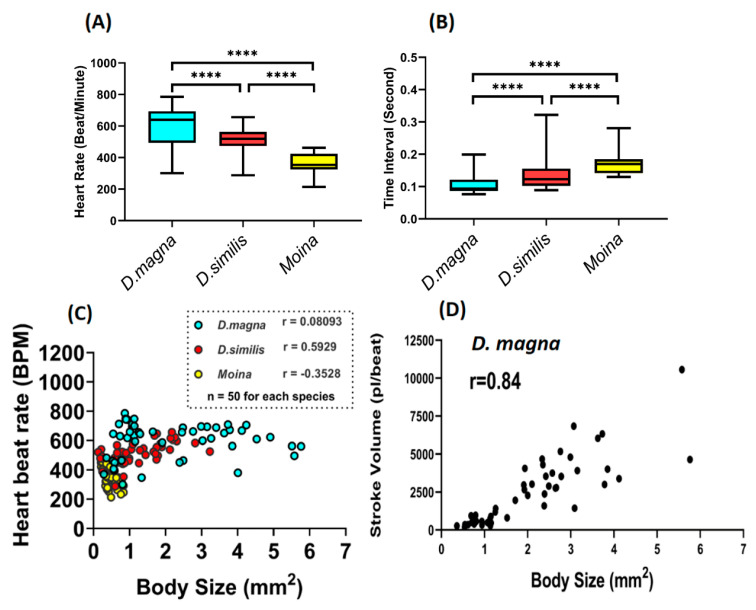
Comparison of the heart rate (**A**) and heat beat interval (**B**) between three different water flea species: *Daphnia magna* (blue), *Daphnia silimis* (red), and *Moina* sp. (yellow) (**C**) The correlation plot for body size and heartbeat among the three water flea species. (**D**) The Pearson correlation plot for body size and stroke volume of *D. magna*. The significance in (**A**) and (**B**) was tested using one-way ANOVA with a non-parametric Mann–Whitney test (**** *p* < 0.001). The sample size for (**A**) to (**D**) is 50 for each water flea species.

In this study, we measured the introductory heart rate for different water flea species using the Image-J method. Among the three species of water fleas, *D. magna* displays the fastest heartbeat (600.30 ± 17.09 bpm), *D. similis* has a lower heart rate (514.70 ± 10.35 bpm), and *Moina* sp. displays the slowest heartbeat (361.30 ± 9.69 bpm) (Figure 2A). The heartbeat time interval is estimated at 0.105 ± 0.003 s per beat for *D. magna*, 0.143 ± 0.008 s per beat for *D. similis,* and 0.173 ± 0.005 s per beat for *Moina* sp. (Figure 2B). The potential correlation between body size and heartbeat for three water fleas was also studied. Different sizes of water fleas were tested, and their body size and heartbeat were measured. *D. magna* displayed an immense body size (with max. 6 mm^2^) compared to the other two species (with max. 3 mm^2^ for *D. silimis* and 1 mm^2^ for *Moina* sp.). Using the Pearson correlation test, we found no correlation between body size and heartbeat in the three water fleas species tested (Figure 2C). However, in this study, we found that body size was correlated with the stroke volume in *Daphnia magna* (Figure 2D). These data demonstrate that the heartbeat of water fleas was size-independent, but stroke volume was size-dependent. Therefore, in this study, normalization of body size was required in order to obtain relevant data for stroke volume.

### 3.4. The Evaluation of the Effect of Temperature Changes on Water Fleas Based on Heartbeat

**Figure 3:**
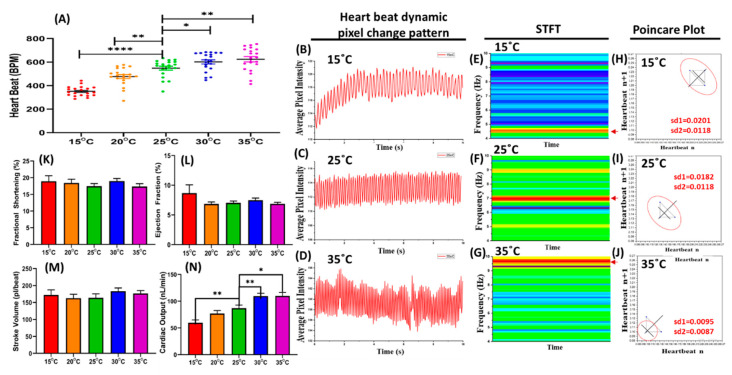
Evaluation of temperature changes on heart rate in *Daphnia magna*. (**A**) The increase of temperature leads to the enhancement of the heartbeat in *D. magna*. The dynamic pixel change pattern extracted from *D. magna* heartbeat (**B**) at 15 °C, (**C**) at room temperature 25 °C, and (**D**) at high temperature 35 °C. (**E**–**G**) Short-time Fourier transform (STFT) and (**H**–**J**) Poincaré plot of *D. magna* after exposure to three different temperatures, at either 15 °C, 25 °C or 35 °C. The effect of temperature changes on fractional shortening (**K**), ejection fraction (**L**), stroke volume (**M**), and cardiac output (**N**). One-way ANOVA with non-parametric Mann–Whitney Test assayed the significance in A, K, L, M, and N (* *p* < 0.05, ** *p* < 0.01, **** *p* < 0.001). The sample size for (**A)** to (**D)** was 50 for each water flea species. The sample size in (**A**), (**K**), (**L**), (**M**), and (**N**) was 20 *Daphnia magna* at each tested condition.

Since the heartbeat of water flea species was effectively measured using the ImageJ-based method, the effect of temperature on heartbeat was then studied. *D. magna,* a commonly used species, was chosen for this experiment. In this study, a specially designed temperature chamber connected to the heater was used to maintain the temperature. An ice cube was put into the chamber to reduce the temperature to 15 °C, and the heater was used to increase the temperature to 35 °C. In these conditions, the cardiovascular function was analyzed every 5 °C. At the lowest temperature, 15 °C, the heart rates were 264.1 ± 13.03 bpm, and doubled to 546.4 ± 36.25 bpm after the shift to 35 °C. Based on our study, the heart rates in *D. magna* were temperature-dependent (Figure 3A, Video S1). This phenomenon is also consistent with the heartbeat dynamic pixel change pattern (Figure 3B–D). The heart rhythm was loosened at the lowest temperature and became more compact in correspondence with the temperature elevation. A short-time Fourier transform (STFT) was used to transform the time interval into the frequency domain (Figure 3E–G). The heartbeat frequency was elevated along with the increment of temperature. The time interval frequency at 15 °C, 25 °C, and 35 °C was 0.3–0.8 Hz, 1.0–1.3 Hz, and 1.4–1.8 Hz, respectively. A Poincaré plot is mainly used for quantifying the heart rate variability (HRV) and has proven to be quite an adequate measure of this marker [34]. Figure 3H–J shows that the highest temperature has a lower SD (standard deviation) value in the Poincaré plot, consistent with the heartbeat dynamic pixel change pattern.

On the other hand, the temperature effect did not alter other cardiovascular parameters, excluding cardiac output. (Figure 3K–N). Fractional shortening (FS) is calculated by measuring the percentage change in heart diameter during the systole period. This is strongly correlated with ejection fraction (EF) [35]. EF is the percentage change in end-diastolic volume. Ejection fraction and stroke volume are two parameters that are commonly measured to evaluate cardiovascular performance. Stroke volume is the difference between end-diastolic and end-systolic volume, or the volume ejected in each heartbeat [36]. Cardiac output is the amount of blood the heart pumps in 1 min. It is determined by multiplying the heartbeat by the stroke volume [33]. Based on Figure 3N, cardiac output was significantly altered at 15 °C, 30 °C, and 35 °C, compared to room temperature (25 °C). This result was consequent to the previous finding. As the temperature increased up to 6 °C, the daphnids became more active and increased their breathing and heartbeat [37].

### 3.5. Blood Flow Measurement for Different Water Flea Species

**Figure 4:**
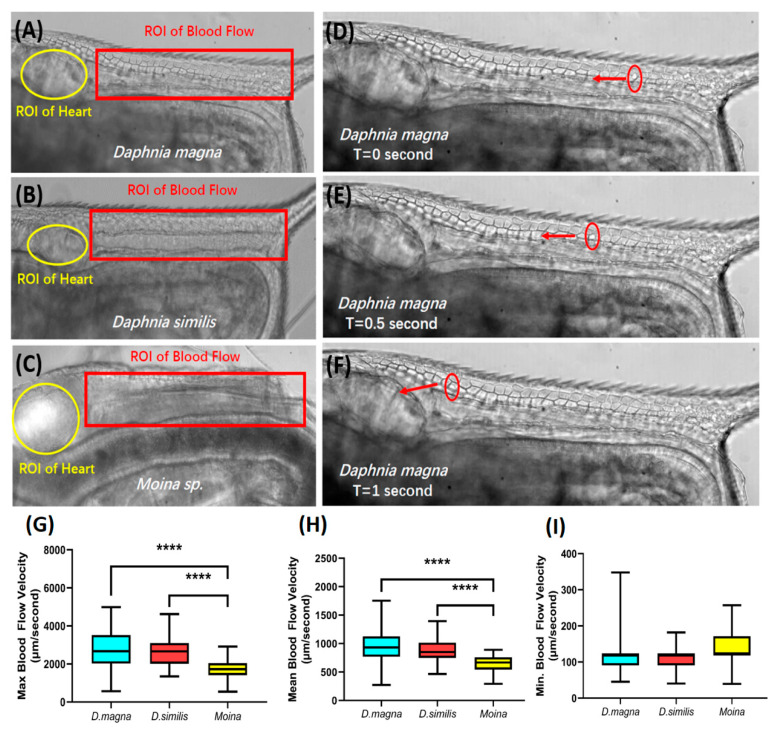
Comparison of the blood flow rates among the three different water flea species: *Daphnia magna*, *Daphnia silimis,* and *Moina* sp. The region of interest (ROI) is used to conduct blood flow velocity calculations (**A**–**C**). Three success panels show a single blood cell (hemocyte) position at different time points (**D**–**F**). The maximal (**G**), medium (**H**), and minimal (**I**) blood flow rate comparisons among the three water flea species. The significance in (**G**), (**H**), and (**I**) was assayed by one-way ANOVA with a non-parametric Mann–Whitney Test (**** *p* < 0.001). The sample number in (**G**), (**H**), and (**I**) is 50 for each water flea species.

Next, we used the ImageJ-based method to measure the blood flow rate for different water flea species. For this purpose, the area posteriorly adjacent to the heart (the dorsal part of the thorax, highlighted by a red rectangle) for the three water flea species was selected as the region of interest (ROI) for blood flow monitoring and calculation (Figure 4A–C). In three water flea species surveyed, we found the hemocytes were pumped out by heart chamber contraction, circulated to the whole body, and finally, returned to the heart chamber. The movements of hemocytes can be precisely tracked and have good linear flow patterns over time, when they travel across the dorsal part of the thorax. The velocity of blood flow was measured by converting video to sequential images and monitoring specific hemocyte movement distance. For example, in *D. magna*, the XY coordination for single hemocyte (labeled by a red circle) was able to be tracked at t = 0 (Figure 4D), t = 0.5 s (Figure 4E), and t = 1 s (Figure 4F), and its movement velocity could be calculated by dividing the distance with time.

Based on Figure 4G–I, it can be seen that there was a significant difference in the maximal and mean blood flow velocity between *Daphnia* species and *Moina* sp. Among the three water fleas, *D. magna* (2780 ± 134.8 μm/s) displays a similar level of max blood flow velocities to that of *D. similis* (2656 ± 105.2 μm/s), and significantly higher level than that of *Moina* sp., at 1711 ± 72.33 μm/s (Figure 4G). In addition, the mean blood flow velocity of *D. magna* (959.9 ± 38.92 μm/s) displays a similar level of blood flow velocity to that of *D. similis* (867.5 ± 27.30 μm/s). In contrast, *Moina* sp. displays a relatively lower blood flow velocity, at 647.3 ± 18.86 μm/s (Figure 4H). The minimal blood flow velocity, on the contrary, displays no difference among the three water fleas (*D. magna* 117.1 ± 6.891 μm/s, *D. similis* 119.1 ± 4.752 μm/s, and *Moina* sp. 132.6 ± 5.574 μm/s, respectively) (Figure 4I).

### 3.6. Toxicity Assessment of Pesticide in Water Fleas Based on Heartbeat and Blood Flow

**Figure 5:**
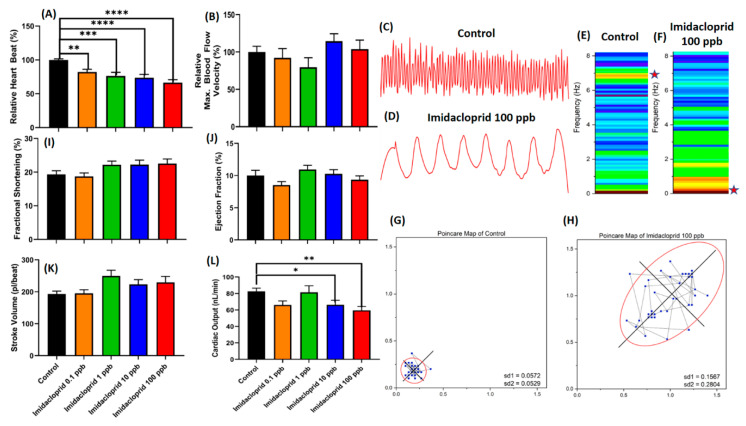
Toxicity evaluation of imidacloprid on cardiovascular performance in *Daphnia magna*. (**A**) The cardiotoxicity of imidacloprid on relative heartbeat and (**B**) relative max. blood flow velocity of *D. magna*. The dynamic pixel change pattern extracted from control *D. magna* heartbeat (**C**) and after treating with 100 ppb of imidacloprid (**D**). (**E**,**F**) Short-time Fourier transform (STFT) and (**G**,**H**) Poincaré plot of control and treated *D. magna*. (**I**,**K**) The effects of imidacloprid on cardiovascular performance of *D. magna,* fractional shortening, ejection fraction, stroke volume, and cardiac output, respectively. The significance in (**A**), (**B**), (**I**), (**J**), (**K**), and (**L**) was assayed by one-way ANOVA with non-parametric Mann–Whitney Test (* *p* < 0.05, ** *p* < 0.01, *** *p* < 0.005, **** *p* < 0.001). The sample size in (**A**), (**B**), (**I**), (**J)**, (**K**), and (**L**) is 15 for each testing condition.

In validating the potential utility of the established method in this study, the pesticide imidacloprid was used to examine the toxicity effect on cardiovascular performance of water fleas. Based on Figure 5, the results indicate that imidacloprid induced dose-dependent inhibition of heartbeat on *D. magna*, even in the lowest concentration of 0.1 ppb (Figure 5A, Video S2). On the contrary, no alteration appeared in maximum blood flow velocity when *D. magna* was challenging with imidacloprid ranging from 0.1 to 100 ppb (Figure 5B). No alteration in other cardiovascular parameters was found (Figure 5I–K, Video S3). However, imidacloprid could significantly reduce cardiac output in *D. magna* at a concentration of 10 ppb and 100 ppb (Figure 5L). The effect of imidacloprid at the highest concentration also caused the heart rhythm to loosen in 10 s (Figure 5C,D). The number of peaks in one second was significantly lower in *D. magna* after exposure to 100 ppb of imidacloprid. The time interval frequencies of control and imidacloprid treatment were 6.7–7.0 Hz, and 0.7–1.0 Hz, respectively (Figure 5E,F). Similarly to the STFT data, the Poincaré plot showed that the highest concentration of imidacloprid treatment, at 100 ppb, had a higher heartbeat irregularity with a higher standard deviation of heartbeat time interval (Figure 5G,H).

## 4. Discussion

Cardiovascular diseases have rapidly become a major threat to the world. The use of animal models to study cardiovascular disease substantially contributes to increasing our understanding of disease pathogenesis, also helping to improve the development of diagnostic techniques, and to verify the effectiveness of different preventative and therapeutic approaches [38]. The cladocerans, especially *Daphnia*, *Ceriodaphnia*, and *Moina*, have been used in aquatic toxicity tests for a long time [39]. In contrast to human beings, who have a closed circulatory system, the Cladocera have an open circulatory system. *Daphnia*’s heart has no compartments or valves and is classified as an open circulatory system [40]. The blood cells (hemocytes) of *Daphnia* are easily observed through the transparent body as they flow rapidly through the body cavity. Furthermore, the heart is relatively large in the water flea and is located beside the brood chamber [41]. *Moina* also belongs to the cladocerans group, which means it has a similar circulatory system to *Daphnia,* although it has a smaller body size [39].

Due to their high sensitivity, many studies have observed the effects of environmental factors on the heart rates of water fleas. Our previous study developed a simple and cost-effective method using high-speed videography and ImageJ software to study cardiovascular performance in zebrafish [28,29]. In the present study, we successfully measured the cardiovascular function in water fleas and its application for toxicity assessment using a similar approach. Using the Time Series Analyzer plug-in in ImageJ, a dynamic pixel change method was used to measure the heartbeat. Based on the dynamic pixel change, the pixel intensity number was observed to increase when hemocytes were pumped out of the heart chamber, defined as a systolic contraction phase, and the higher peak rhythm was formed at that time. On the contrary, a lower peak rhythm was formed during the relaxation or diastolic phase [29]. Next, LoG segmentation in the TrackMate ImageJ plug-in was used to track single blood cell motion. This plug-in could provide single-particle tracking via segmenting and following the object over time. The velocity of blood cell movement can be calculated according to the *x* and *y* coordinates [28]. By utilizing this method, we are able to detect blood flow velocity in water fleas for the first time, to our knowledge. We showed that different water flea species have different performance in heart rate and blood flow velocity, with *D. magna* showing the highest result among tested water flea species and thus being recognized as a suitable model for cardiovascular performance studies. Moreover, by measuring the single chamber heart’s diameter, we demonstrated the successful measurement of other cardiovascular parameters, including ejection fraction, fractional shortening, stroke volume, and cardiac output in water fleas. The study of stroke volume and cardiac output also has been performed in other small crustaceans [42] and in *Daphnia magna* [30]. The cardiovascular response can be better understood by evaluating the heart rate, stroke volume, and cardiac output, rather than evaluating the heart rate alone [42]. Therefore, we proposed that multiple cardiovascular parameters should be measured to fully recapitulate the physiological or toxicological responses in water fleas.

In this study, temperature changes and the use of pesticide were studied to see the variety of cardiovascular function in *Daphnia magna*. A specially designed temperature chamber connected to a heater was used to maintain the temperature changes. The temperature chamber used in this study was sufficient to maintain the temperature conditions. Previous studies used a manual method to evaluate temperature changes on the heartbeat in daphnids. In one such study, five minutes were allowed for daphnids to adjust the water temperature, and the water temperature was measured. The water was then changed into cold water, and warm water obtained from a water bath and acclimation were carried out for 5 min before counting the heartbeat [43]. Compared to the previous method, in this study, we proved that the temperature could be well maintained by using such a temperature chamber and the observation could be done precisely. Based on the results, we found that the heartbeat was temperature-dependent in water fleas. This result was consistent with previous findings showing that daphnids become more active and increase their rate of breathing and heartbeat when temperature increased up to 6 °C [37]. Another method also used cold water at 10 °C to slow down the heartbeat in *D. magna* [44]. An increase in temperature can increase the metabolism of the organism and decrease its oxygen concentration. Consequently, a lack of oxygen could result in an insufficient amount of oxygen in the blood, Therefore, the heart would have to work harder to pump blood throughout the body, making the heartbeat increase [37]. 

According to Wilson et al. (2011), cardiac output increases during whole-body heating but does not significantly alter during whole-body cooling. On the contrary, stroke volume remains the same or slightly increased by both thermal effects. This statement was in line with our study, which showed that only the highest temperature could alter cardiac output. The enhancement of the heartbeat also generates an increase in cardiac output. Meanwhile, no significant result was observed in stroke volume for all the temperatures studied. The increase in temperature elevates the demand for O_2_ and leads to an increase in cardiac output. To our knowledge, the study of the ejection fraction and fractional shortening in daphnids remains limited. Fractional shortening is an excellent indicator used to study cardiac performance abnormalities. It has a strong correlation with ejection fraction. Both of these parameters are affected by end-diastolic and end-systolic conditions [45]. Based on this study, temperature changes do not affect those parameters.

Imidacloprid (1-(6-chloro-3-pyridylmethyl)-2-nitroiminoimidazolidine) belongs to a major new synthetic insecticides class called neonicotinoids, and is widely used to control insect pests on crops and fleas on domestic animals. However, these insecticides are found to affect non-target organisms, including humans, depending on the agent and the exposure [46]. Imidacloprid is the fastest-growing insecticide in sales due to its low selectivity for insects and its apparent safety for mammals [47,48]. Although imidacloprid is currently being considered as a substitute for other existing pesticides, its risks still need to be assessed. Imidacloprid is known as a nicotinic acetylcholine receptor (nAChR) agonist, and was found to significantly reduce the heartbeat and blood flow, and lead to malformation in zebrafish [49]. Another study also showed that neonicotinoid reduces the heartbeat in *D. magna* after 48 h of exposure [50]. A similar result was found in this study, showing that imidacloprid significantly reduces the heartbeat in *D. magna* at all concentrations tested. However, limited studies of the effect of imidacloprid on other cardiovascular parameters have been reported in the previous literature. This study found that imidacloprid could only alter the cardiac output in the highest concentration, which is 10 ppb and 100 ppb. Other cardiovascular parameters like stroke volume, ejection fractioning, and fraction shortening remain unaltered after imidacloprid exposure. A previous study reported that imidacloprid’s acute 48-h mean lethal concentration (LC_50_) value with regards to *D. magna* was 10–17 ppm [51], and the acute toxicity value (48-h mean effective concentration (EC_50_)) was 43 ppm [52]. In addition, based on data from the ECOTOX database, imidacloprid’s acute toxicity (48-h LC_50_) to *D. magna* was reported to be 10–65 ppm [53]. However, in this study, we found cardiovascular performance is a sensitive marker for pesticide toxicity assessment. The cardiac parameter alteration could be detected in *D. magna* even if the given dose of imidacloprid was 10,000-fold less than the reported EC_50_ or LC_50_.

In this study, we overcome several problems that were limitations of previously published methods. Campbell et al. [54] reported using a droplet of water to stabilize the temperature of water flea heartbeat measurements. In this study, we introduced the usage of a temperature-controllable chamber as it can stabilize the ambient temperature at the time of recording without the need to consistently add water droplets while recording. Furthermore, Oujesky et al. [55] reported a method to remove the surrounding water to reduce the body movement for better *D. magna* heart rate recording. As water fleas are aquatic animals, the removal of water causes some stress. In our study, we propose a simple method by using methylcellulose as a mounting solution to reduce the movement of water fleas for short-term cardiovascular performance endpoint recoding.

**Figure A2:**
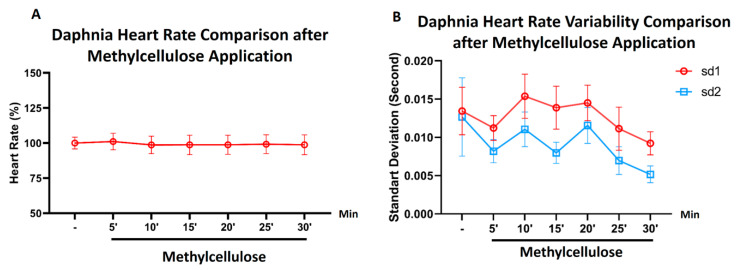
The comparison of *Daphnia magna*’s heartbeat rate (**A**) and heartbeat regularity (**B**) after application of methylcellulose as a mounting medium. The heartbeat rate and heart rate variability for *D. magna* were measured before application of 3% methylcellulose (-) and every 5 min after methylcellulose application. The data are shown as mean ± SEM (*n* = 10). Degree of significance was calculated using repeated measure one-way ANOVA (A) and Friedman two-way ANOVA. No significant difference for either heartbeat rate or heartbeat regularity was detected between control and methylcellulose-treated groups.

Although the method reported in this study provided a simple and fast protocol for measuring cardiovascular performance in water fleas, some limitations attracted our attention and need to be overcome in future studies. First, the use of methylcellulose to immobilize water fleas might reduce the limb movement and reduce their ventilation for oxygen exchange. Our pretesting data proved that aspects of cardiac performance like heartbeat rate and heartbeat regularity are unaffected by short-term mounting with methylcellulose within 30 min of observation (Appendix A, Figure A2). However, for long-term, continuous, and unbiased cardiac physiology observation in water fleas, we have proposed that a specially-designed viewing chamber will be required, in order to prevent any possible cardiac rhythm alterations due to the hypoxia problem [56]. Second, because we used methylcellulose to reduce the movement of Daphnids, several other parameters including thoracic limb movement, mandible movement, and second antennae movement could not be calculated, as the movement of these organs was obstructed. However, this helped us to focus on the cardiovascular system. The movement of the region of interest is of great importance in video imaging-based methods like the one we propose. Third, the current ImageJ-based method still suffers from a tedious operation/calculation process, limiting overall assay throughput. The use of some automation tools like macros [57] or artificial intelligence (AI)-based methods can be considered in order to overcome these limitations [58]. For example, Akerberg et al. (2019) developed a deep learning-based method to automatically measure volumetric assessments of cardiac function in zebrafish [58]. We believe that by adopting a similar approach, more high-throughput and convenient methods will be invented to promote water fleas as an excellent lower invertebrate model for cardiovascular studies.

## Supplementary Materials

The following are available online at https://www.mdpi.com/2076-2615/10/9/1587/s1, File S1, Standard operation protocol (SOP) for cardiovascular measurement in water fleas. Video S1. Temperature effects on *Daphnia magna* heartbeat. *Daphnia magna* were kept at either 15 °C (left), 25 °C (middle), or 35 °C (right). This slow-motion video is played at 7× slowed speed. Video S2. Pesticide exposure effects on *Daphnia magna* heartbeat. *Daphnia magna* were exposed to either control (left) or 100 ppb imidacloprid (right), and the heartbeat was recorded with slow-motion video at 7× slowed speed. Video S3. Pesticide exposure effects on *Daphnia magna* blood flow. *Daphnia magna* were exposed to either control (left) or 100 ppb imidacloprid (right), and the blood flow was recorded with slow-motion video at 7× slowed speed.
