# Supplementary material for: Cardiovascular Performance Measurement in Water Fleas by Utilizing High-Speed Videography and ImageJ Software and Its Application for Pesticide Toxicity Assessment
Source: Animals (Basel). 2020 Sep 5;10(9):1587. doi: 10.3390/ani10091587 (PMC7552287; doi:10.3390/ani10091587)

Supplementary protocol

Cardiovascular Performance Measurement in Water Fleas by Utilizing High-speed Videography and ImageJ Software and Its Application for Pesticide Toxicity Assessment

Fiorency Santoso 1,2, Viacheslav V. Krylov 3, Agnes L. Castillo 4, Ferry Saputra 1, Hong-Ming Chen 5, Hong-Thih Lai 5 * and Chung-Der Hsiao 1,6,7 *

1. Department of Bioscience Technology, Chung Yuan Christian University, Chung-Li 320314, Taiwan

2. Master program of Nanotechnology, Chung Yuan Christian University, Chung-Li 320314, Taiwan

3. Papanin Institute for Biology of Inland Waters, Russian Academy of Sciences, Borok, Nekouzskii raion, Yaroslavl oblast, 152742 Russia

4. Faculty of Pharmacy, The Graduate School and Research Center for the Natural and Applied Sciences, University of Santo Tomas, Manila 1008 Philippines

5. Department of Aquatic Biosciences, National Chiayi University, 300 Syuefu Rd. Chiayi, 60004 Taiwan

6. Department of Chemistry, Chung Yuan Christian University, Chung-Li 320314, Taiwan

7. Center for Nanotechnology, Chung Yuan Christian University, Chung-Li 320314, Taiwan

* corresponding to Hong-Thih Lai (htlai@mail.ncyu.edu.tw); Chung-Der Hsiao (cdhsiao@cycu.edu.tw)

Required Software

VirtualDub

ImageJ

Excel

ORIGIN® 9.1
Data Analysis and Graphing Software
www.originlab.com

NEW!
Prism 8 Portable
Analyze, graph and present your scientific work.

Outline of analysis pipeline

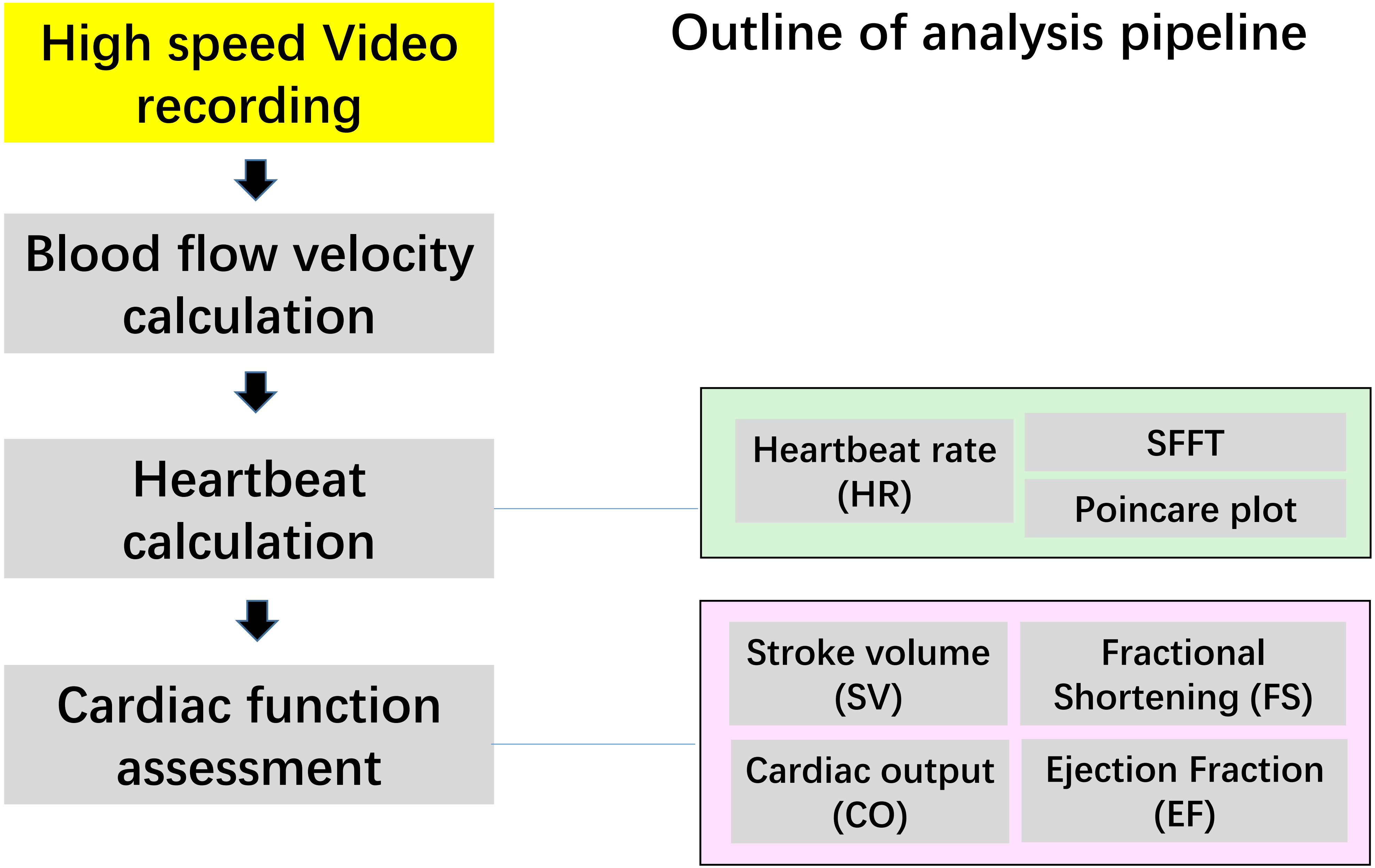

1

Video Capture Apparatus

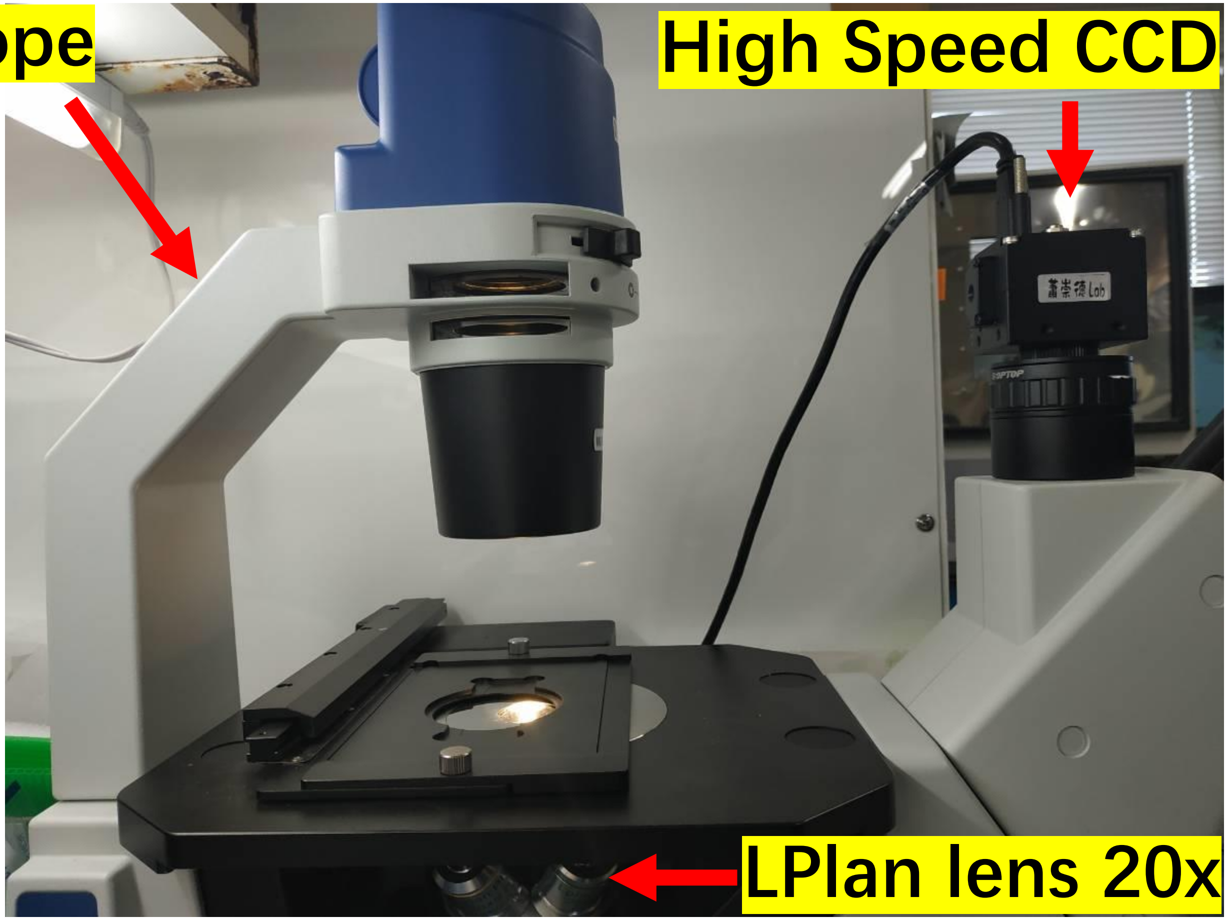

To capture heart and blood flow images

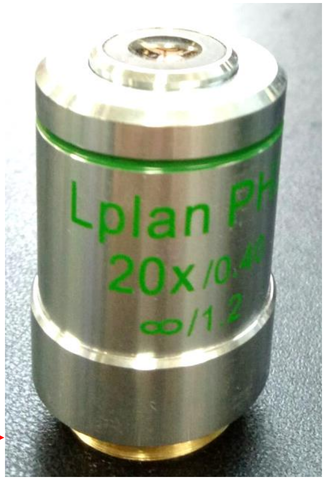

Video Recording using HiBestViewer App
(AZ Instrument, Taiwan)

1

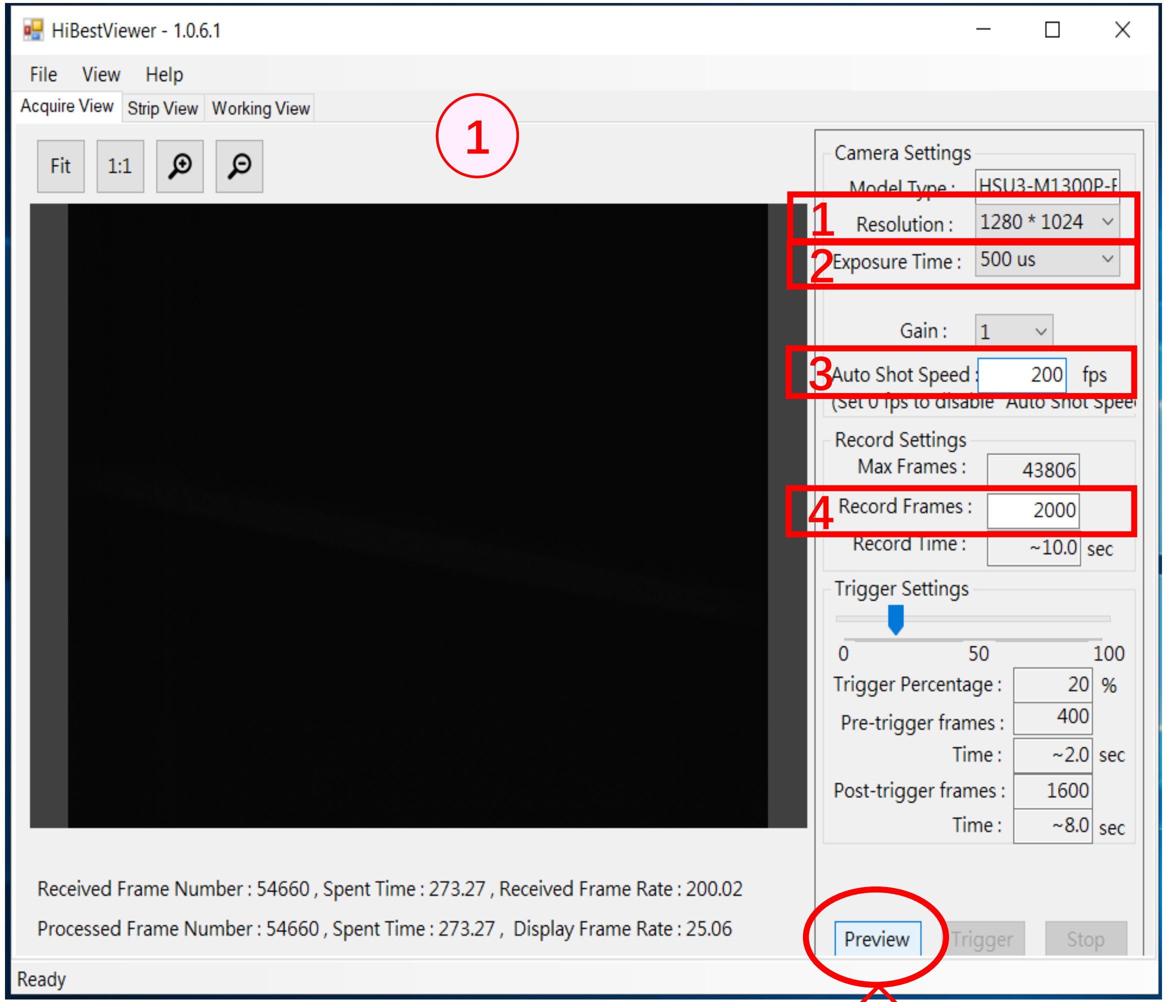

5. After setting up, choose preview

- **Resolution :** measure used to describe the sharpness and clarity of an image.
- **Exposure time :** length of time when the digital sensor inside the camera is exposed to light. **The resolution & exposure time supported by camera is limited, so user is only allowed to select appropriate one.**
- **Auto Shot Speed :** how many photos the unit can take within one second (fps)
- **Record Frames:** Set up the number of images which want to be stored. User can set up the value based on requirements, but the maximum cannot be over the former field (Max Frames), and the minimum cannot be less than or equal to 0

Preview Result (*Daphnia magna* heart)

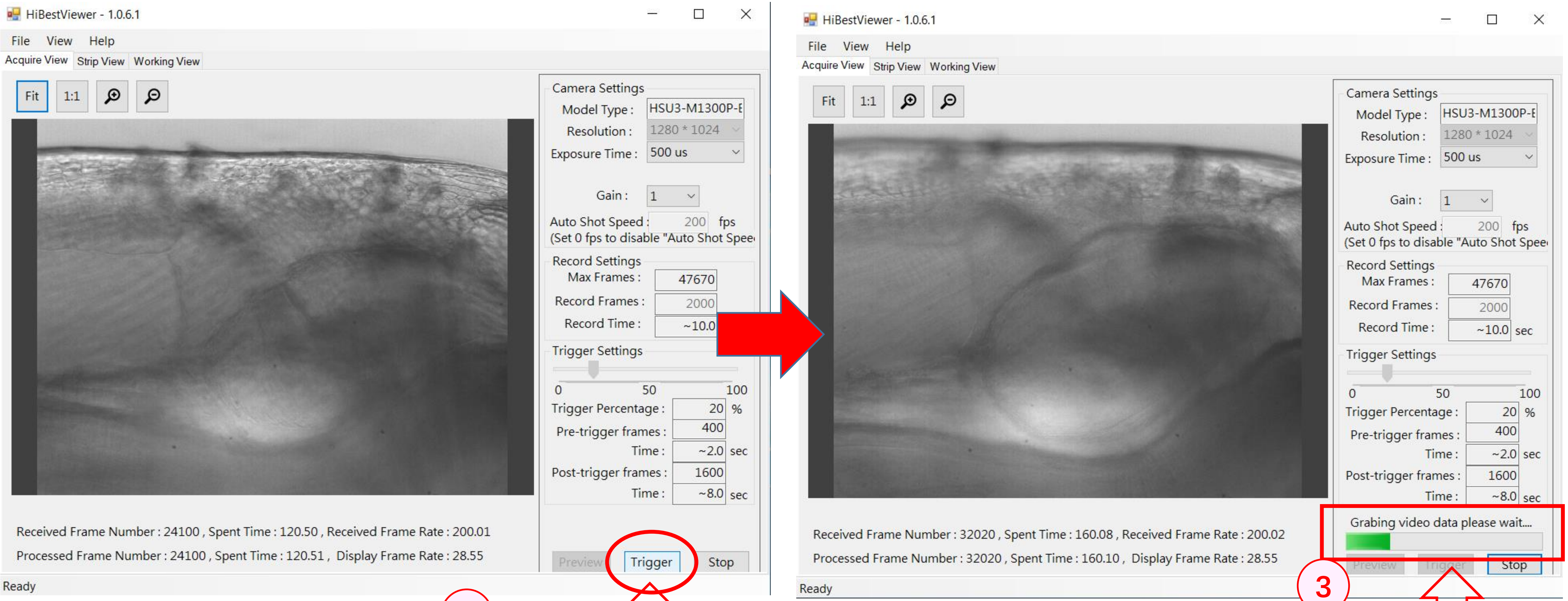

2 Perform **Trigger** to start video recording

3 Video is recorded @ 200 fps

Preview Result

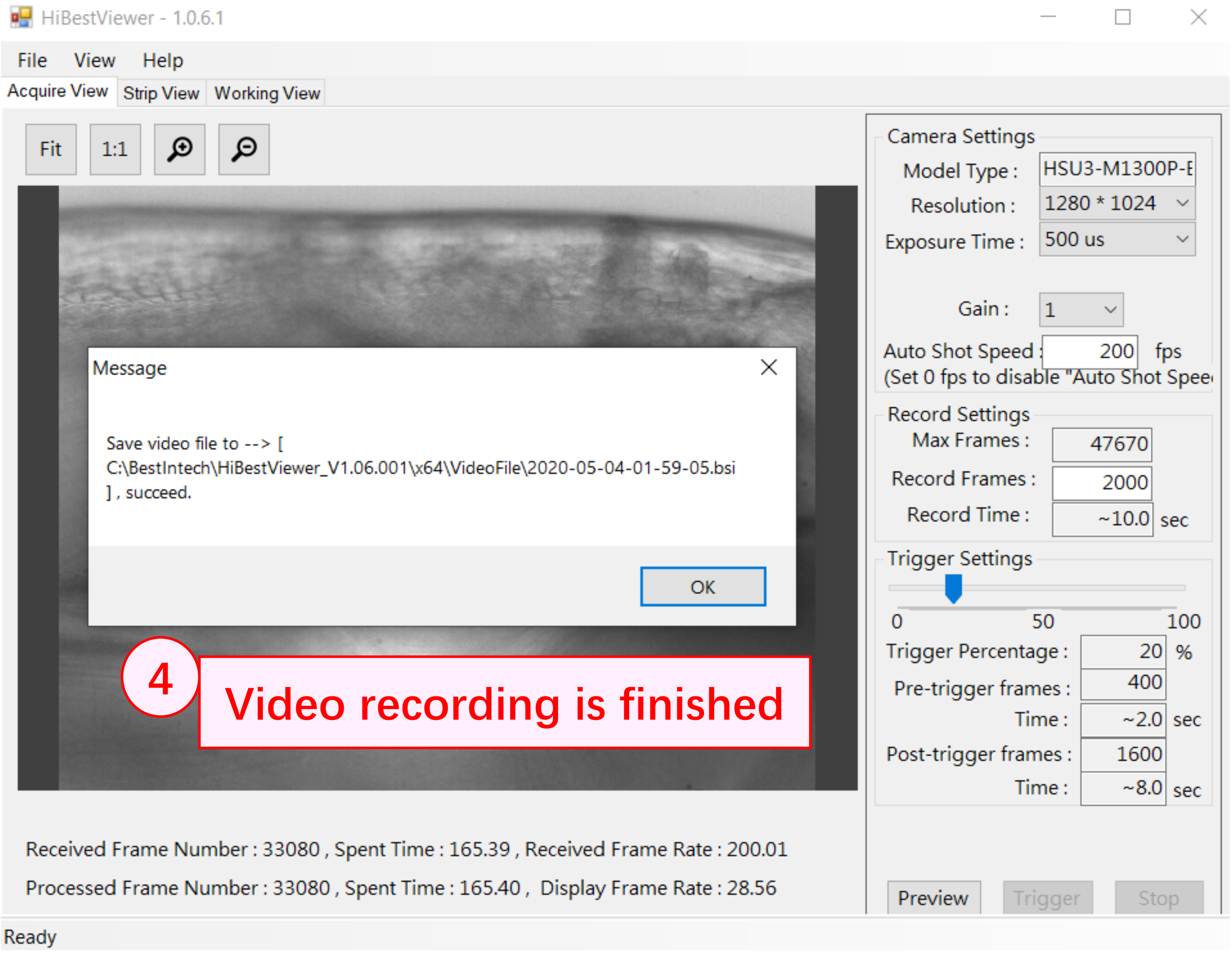

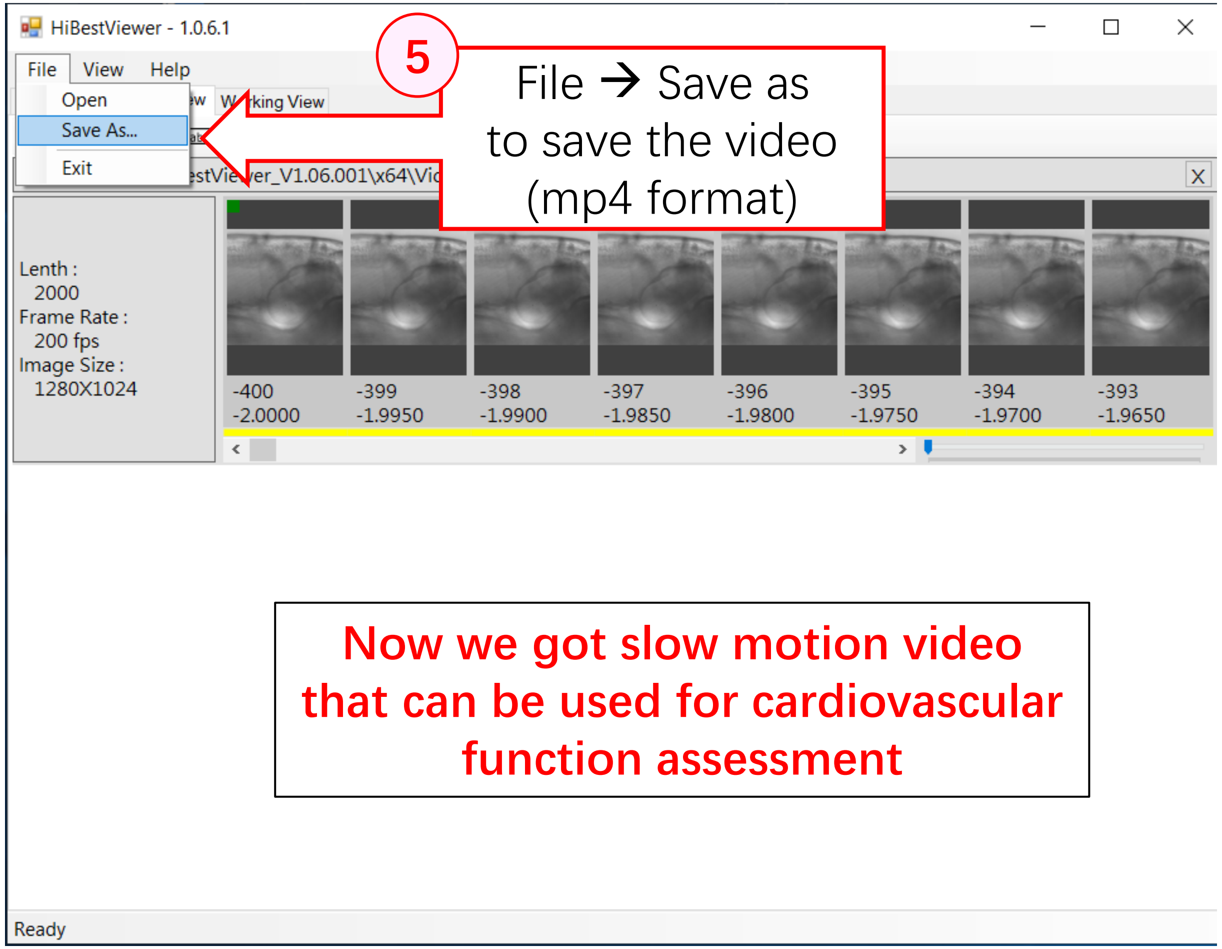

Convert to Uncompressed Format in VirtualDub

Virtual Dub will convert the original video (.mp4 format into .avi format)

ImageJ can only proceed file format (uncompressed AVI) from Virtual Dub

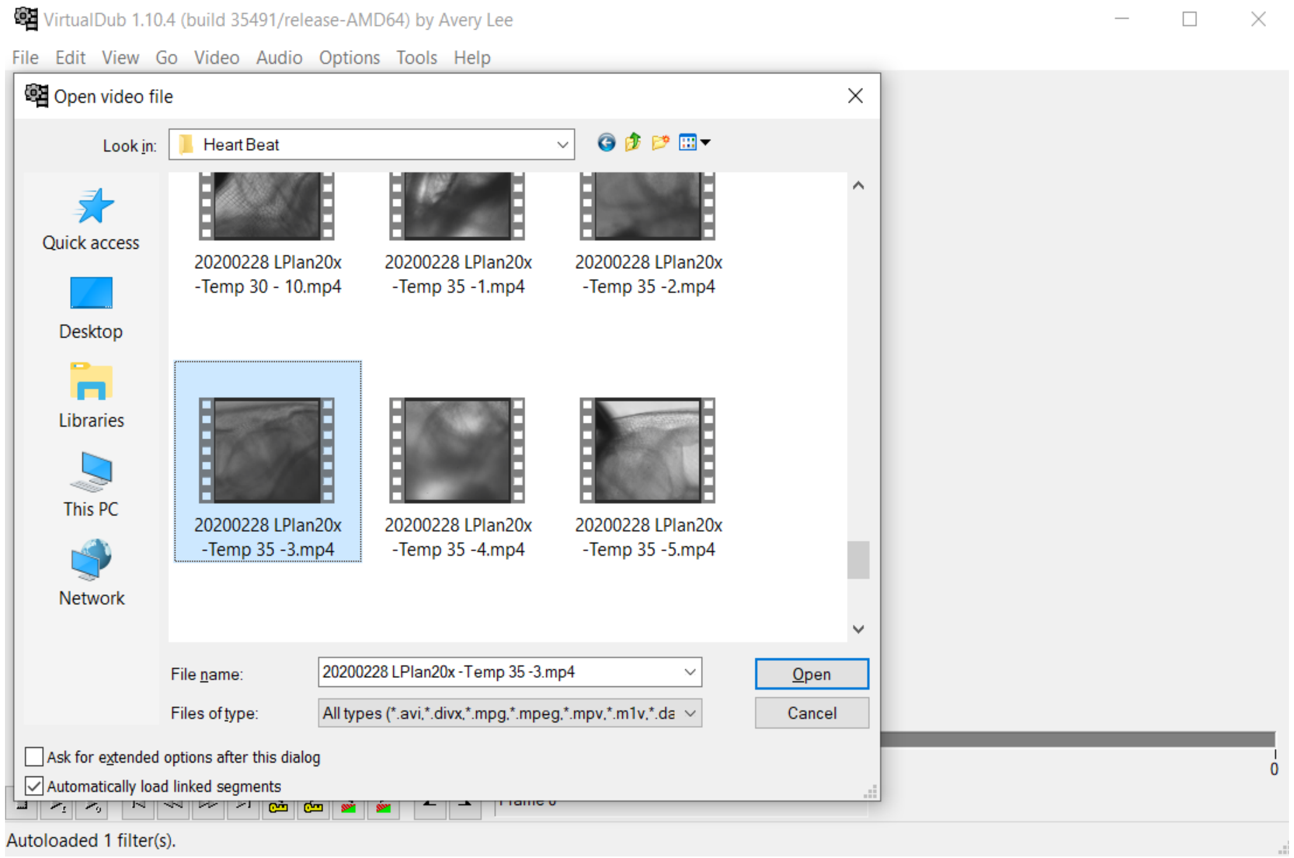

- Click Ctrl+O to select the video file
- Click F7 function key to convert the file format to uncompressed AVI

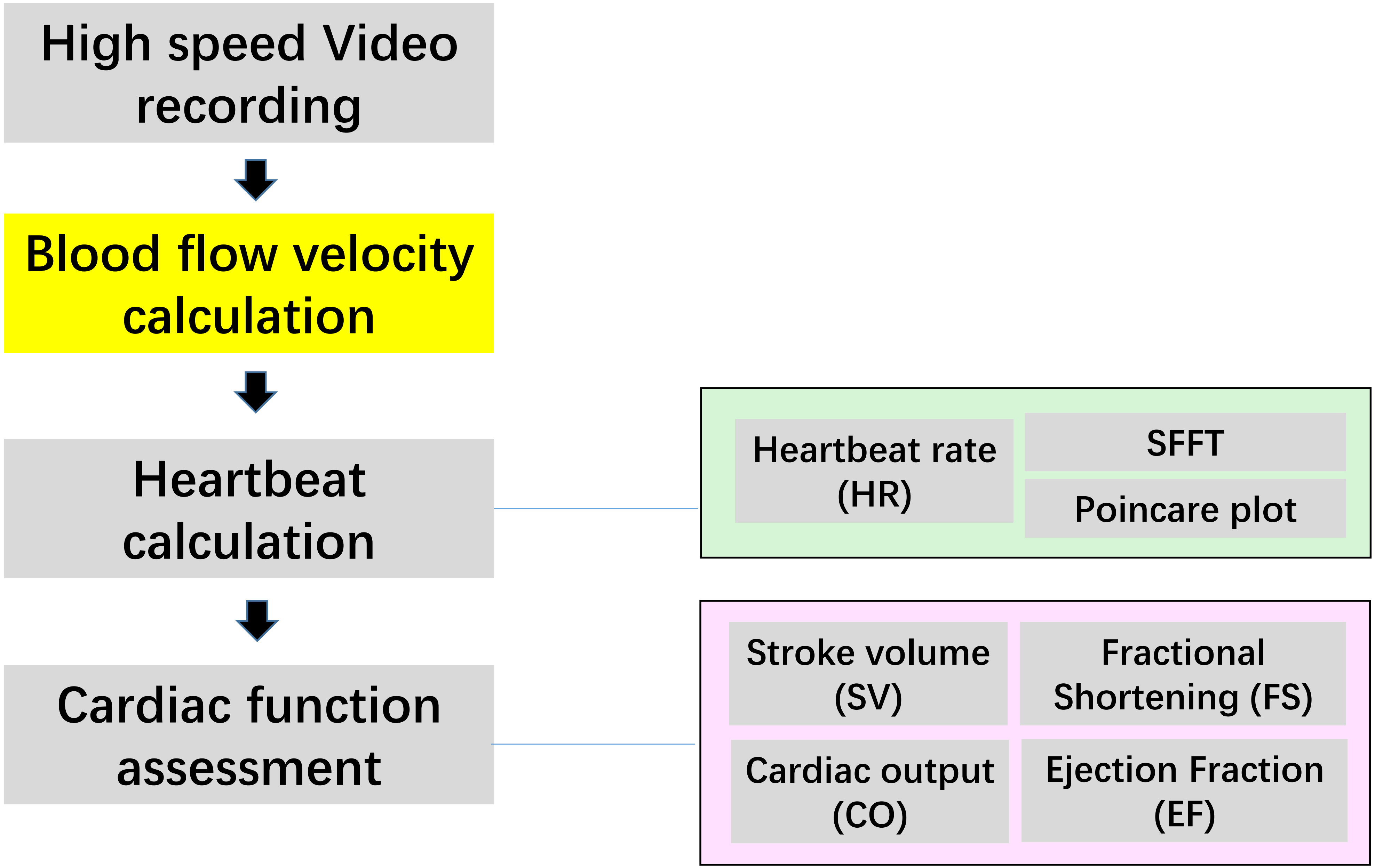
High speed Video recording
Blood flow velocity calculation
Heartbeat calculation
Cardiac function assessment
Heartbeat rate (HR)
SFFT
Poincare plot
Stroke volume (SV)
Fractional Shortening (FS)
Cardiac output (CO)
Ejection Fraction (EF)

Open the Video and Convert it into **Grayscale**

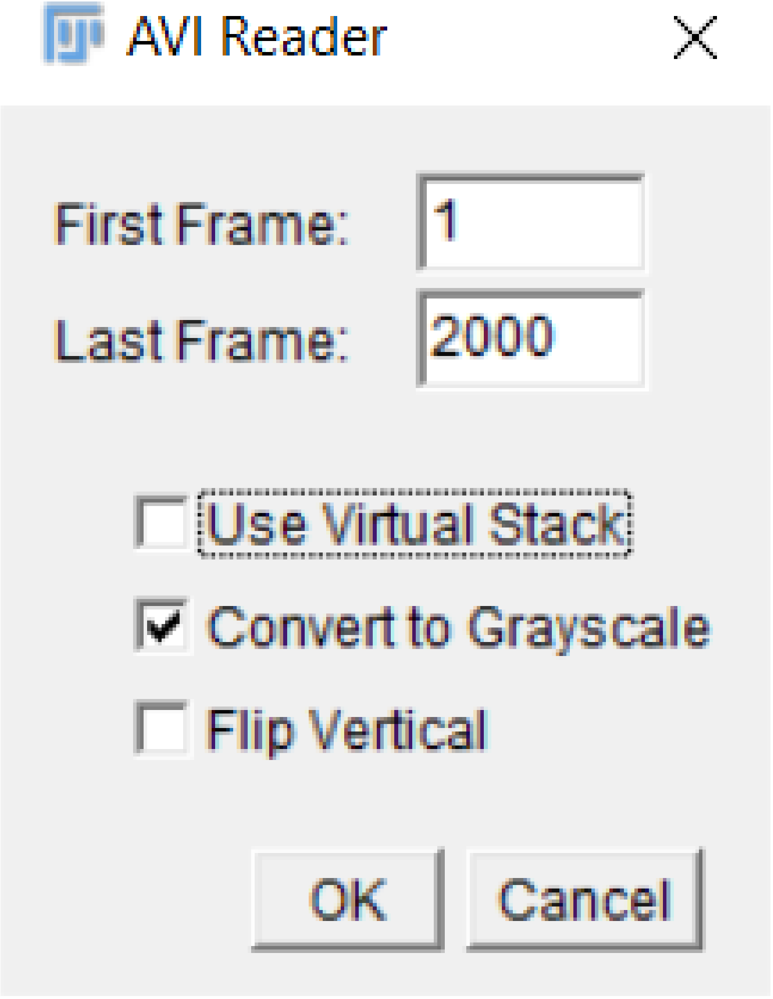

- Drag the video from Virtual Dub format to the ImageJ and convert it into grayscale

Use MTrackJ plugin for blood flow calculation

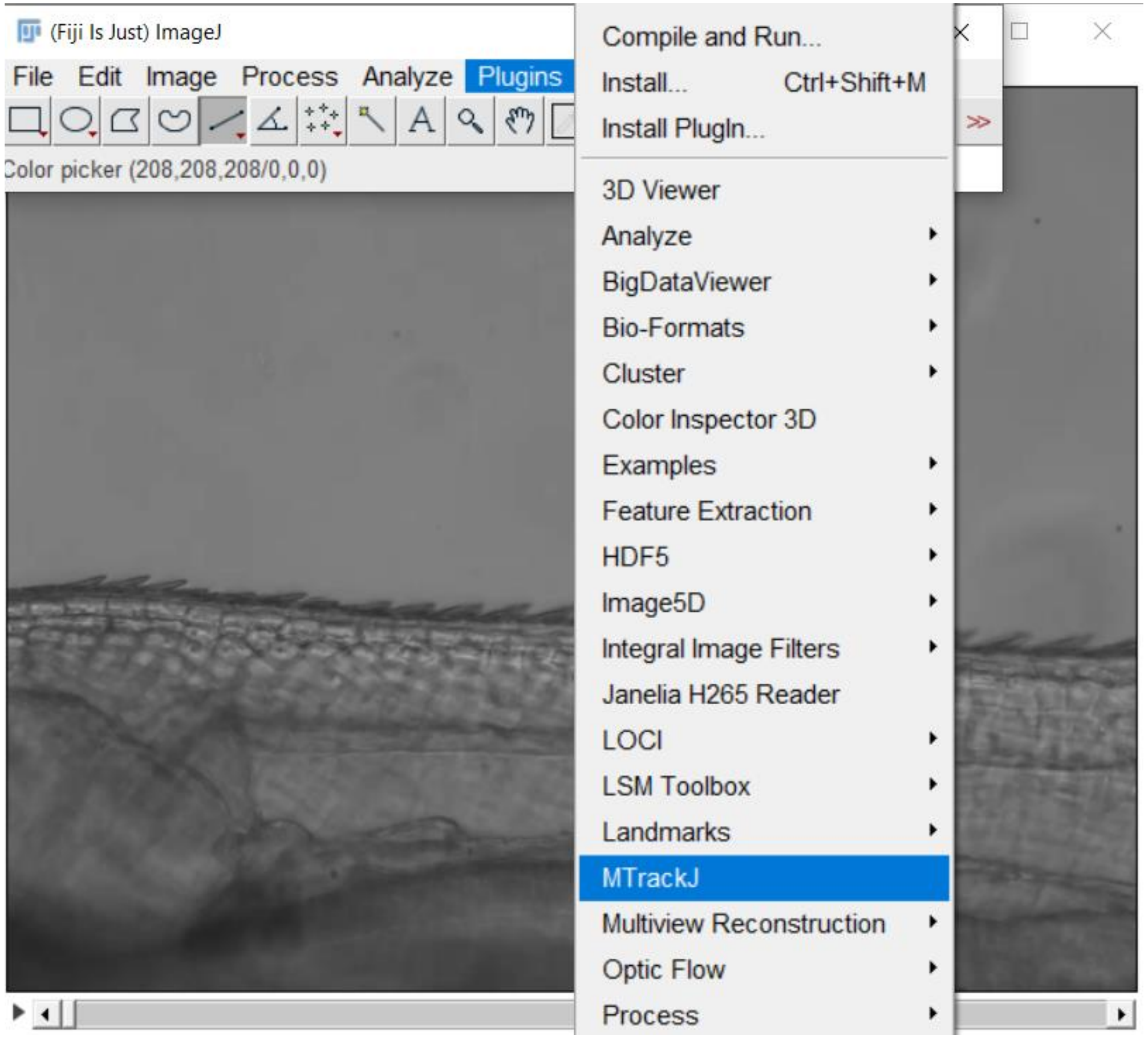

- Select Plugins -> MTrackJ

MTrackJ is used for tracking of moving objects in image sequences and the measurement of track statistics.

MTrackJ Setup for single hemocyte tracking

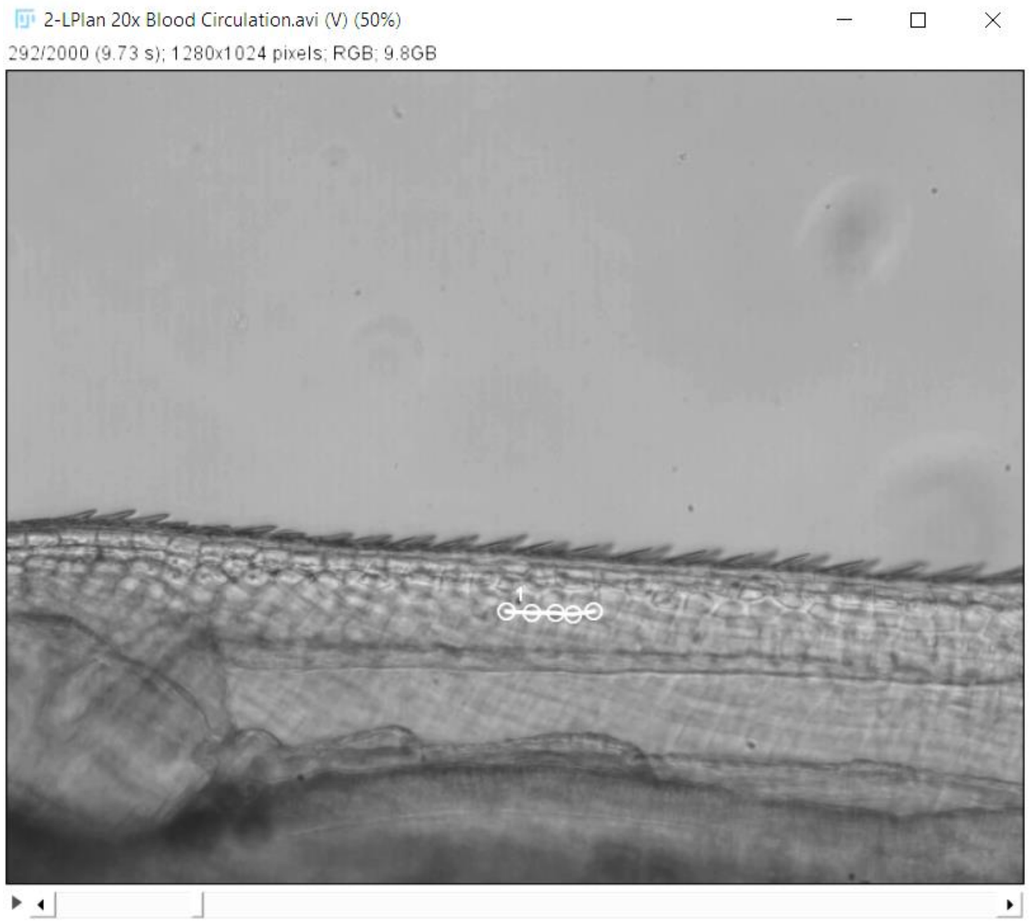

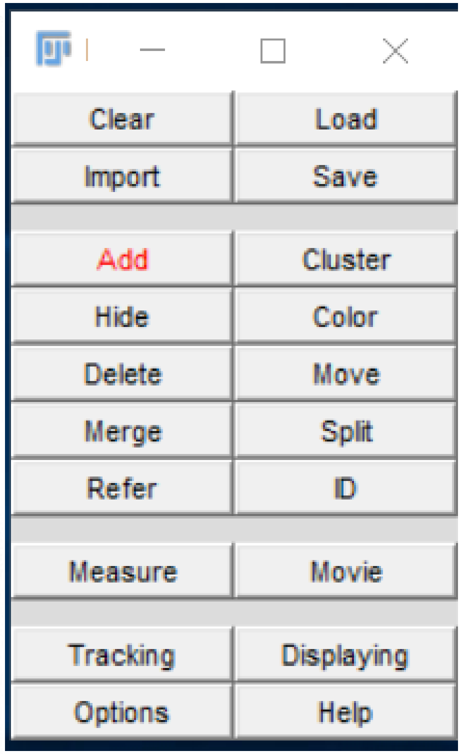

- Select add, then choose blood cell with highest velocity (here we usually select 5 positions for single moving hemocyte)

Measure the Blood Flow Distance

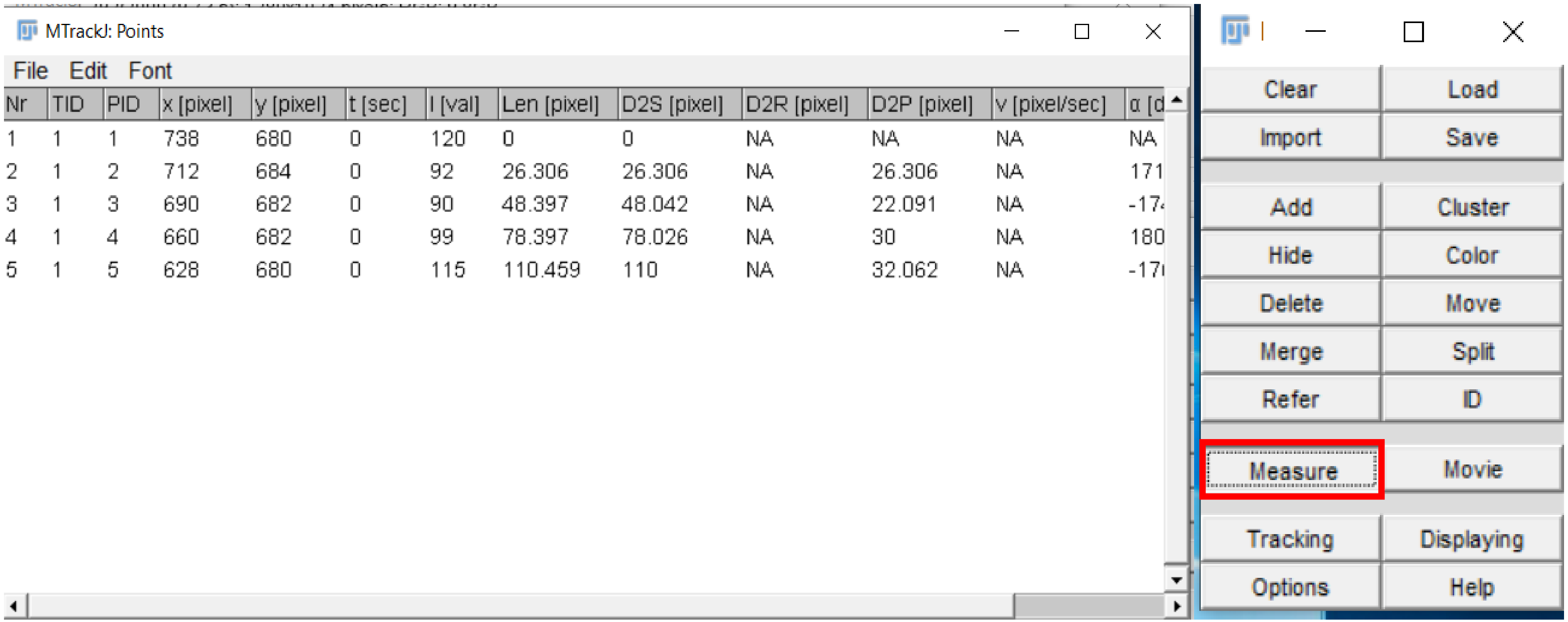

Nr	TID	PID	x [pixel]	y [pixel]	t [sec]	I [val]	Len [pixel]	D2S [pixel]	D2R [pixel]	D2P [pixel]	v [pixel/sec]	α [d
1	1	1	738	680	0	120	0	0	NA	NA	NA	NA
2	1	2	712	684	0	92	26.306	26.306	NA	26.306	NA	171
3	1	3	690	682	0	90	48.397	48.042	NA	22.091	NA	-17[illegible]
4	1	4	660	682	0	99	78.397	78.026	NA	30	NA	180
5	1	5	628	680	0	115	110.459	110	NA	32.062	NA	-17[illegible]

- Select **measure** to get the raw data and copy it to Excel
- MTrackJ measurement is used to discover the **x,y coordinates** of each blood cell, and we can further convert it into moving distance

	A	B	C	D	E	F	G	H	I	J	K	L	M	N	O
1	Number	TID	PID	x (pixel)	Δx (pixel)	y (pixel)	t (sec)	I (val)	Len (pixel)	D2S (pixel)	D2R (pixel)	D2P (pixel)	v (pixel/sec)	α (deg)	Da (deg)
2	1	1	1	738	26	680	0	120	0	0	NA	NA	NA	NA	NA
3	2	1	2	712	22	684	0	92	26.306	26.306	NA	26.306	NA	171.254	NA
4	3	1	3	690	30	682	0	90	48.397	48.042	NA	22.091	NA	-174.81	13.941
5	4	1	4	660	32	682	0	99	78.397	78.026	NA	30	NA	180	-5.194
6	5	1	5	628		680	0	115	110.459	110	NA	32.062	NA	-176.42	3.576
7															
8															
9															
10															
11															
12															

Only X position is used here for linear velocity calculation (since blood cell moving from left to right direction in our recorded videos)

- Calculate the Δx by substrate the x+1 with x position
- Choose **five points** from the same blood cell for locomotion tracking
- Choose at least **4 independent blood cells** (with the highest velocity) for data duplicate
- Pick the cell with highest number of Δx to measure the **maximum blood flow distance**

Select Region of Interest (ROI)

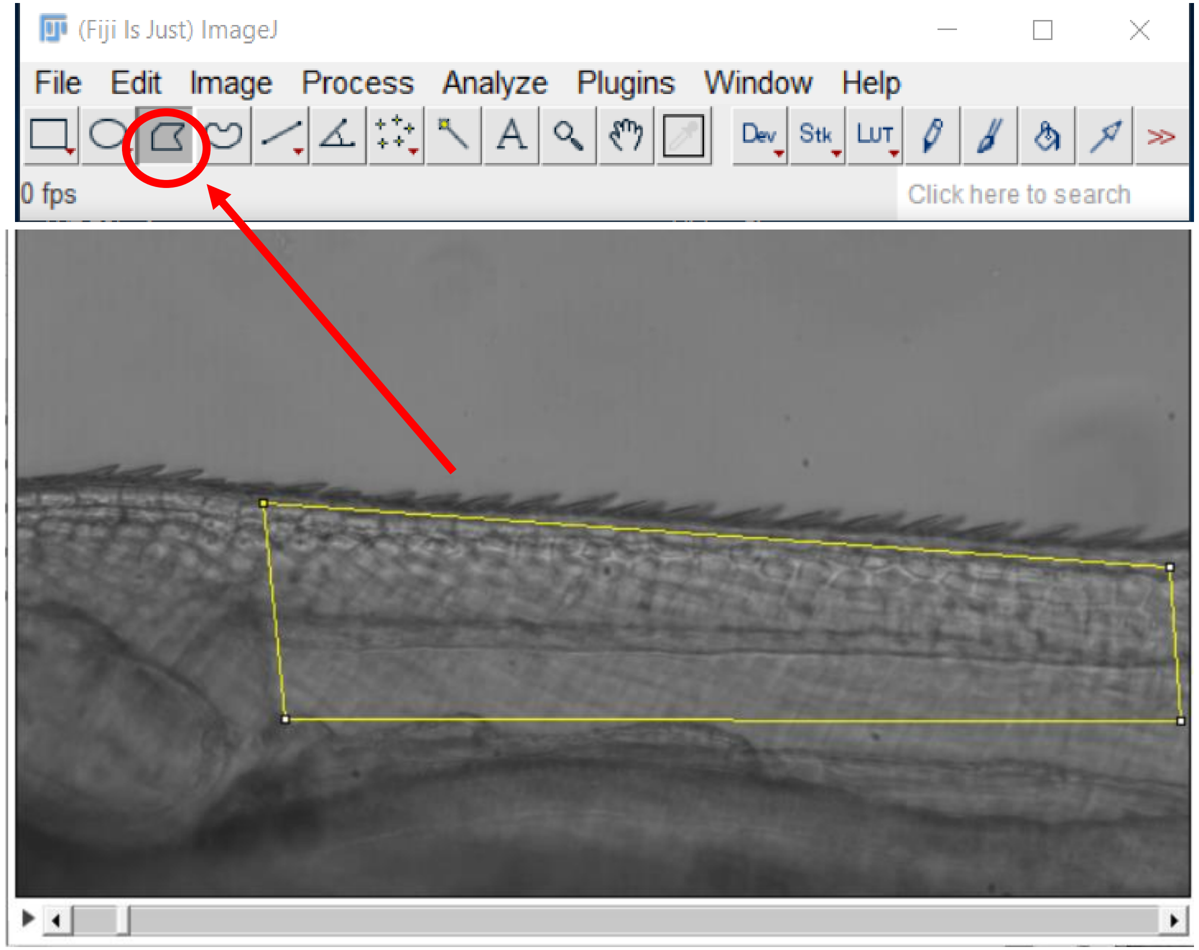

- Choose the polygon section and selections the region that are going to be analyzed (blood chamber)

Clear outside to simplify the analysis complexity

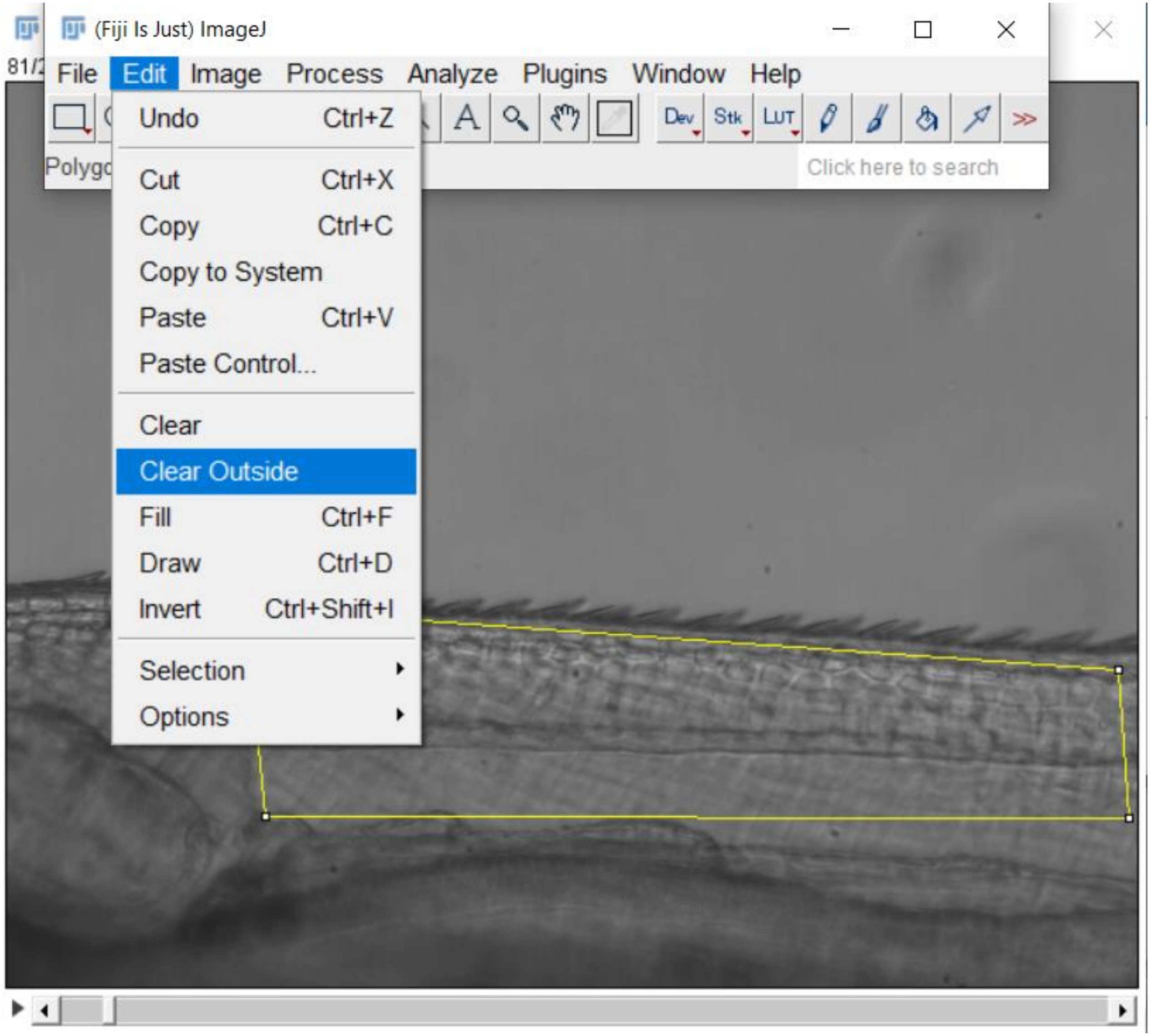

- Erase the area outside the current image selection ROI to ease the analysis

Clear Outside Result

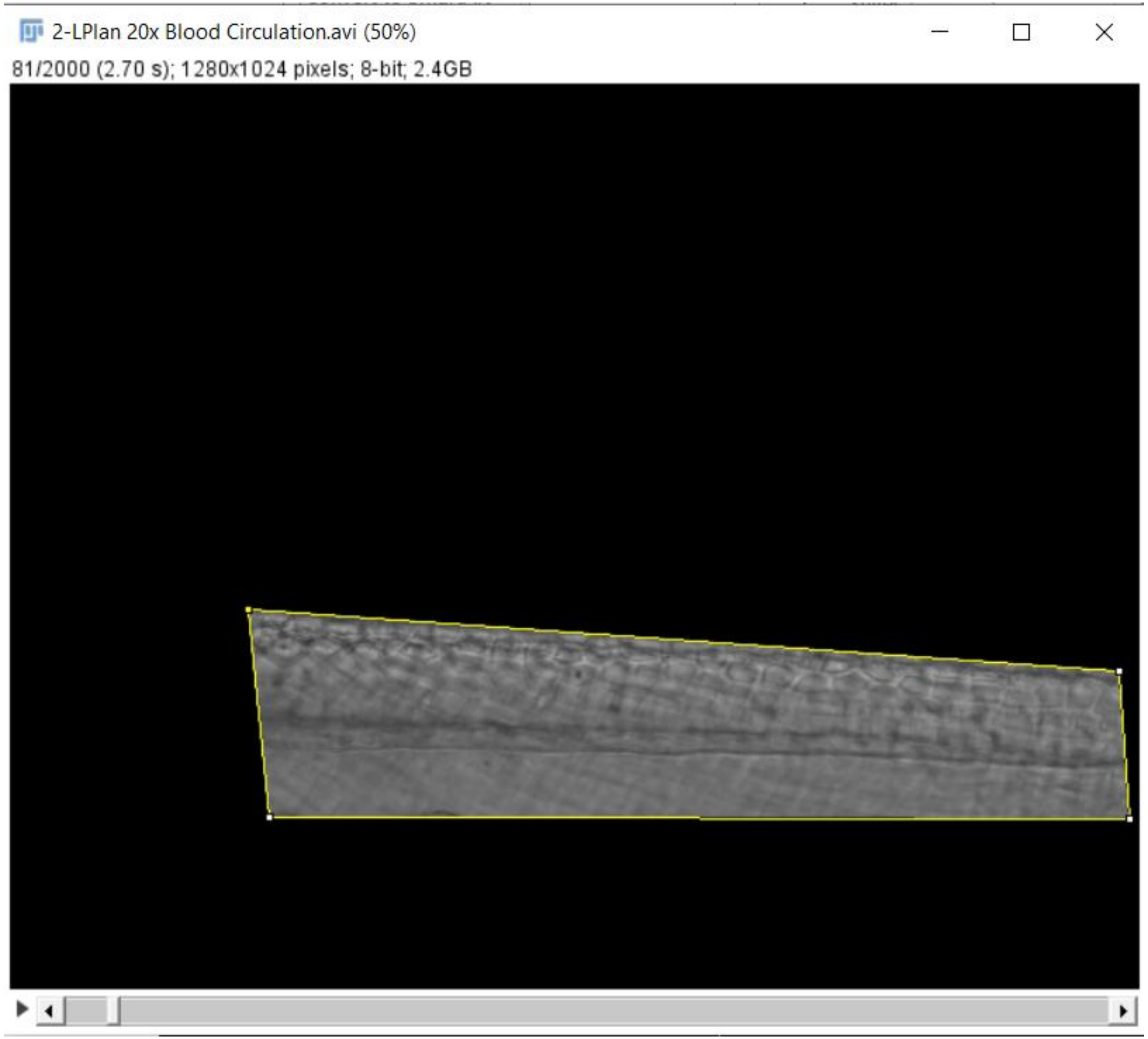

Invert the Selected Area

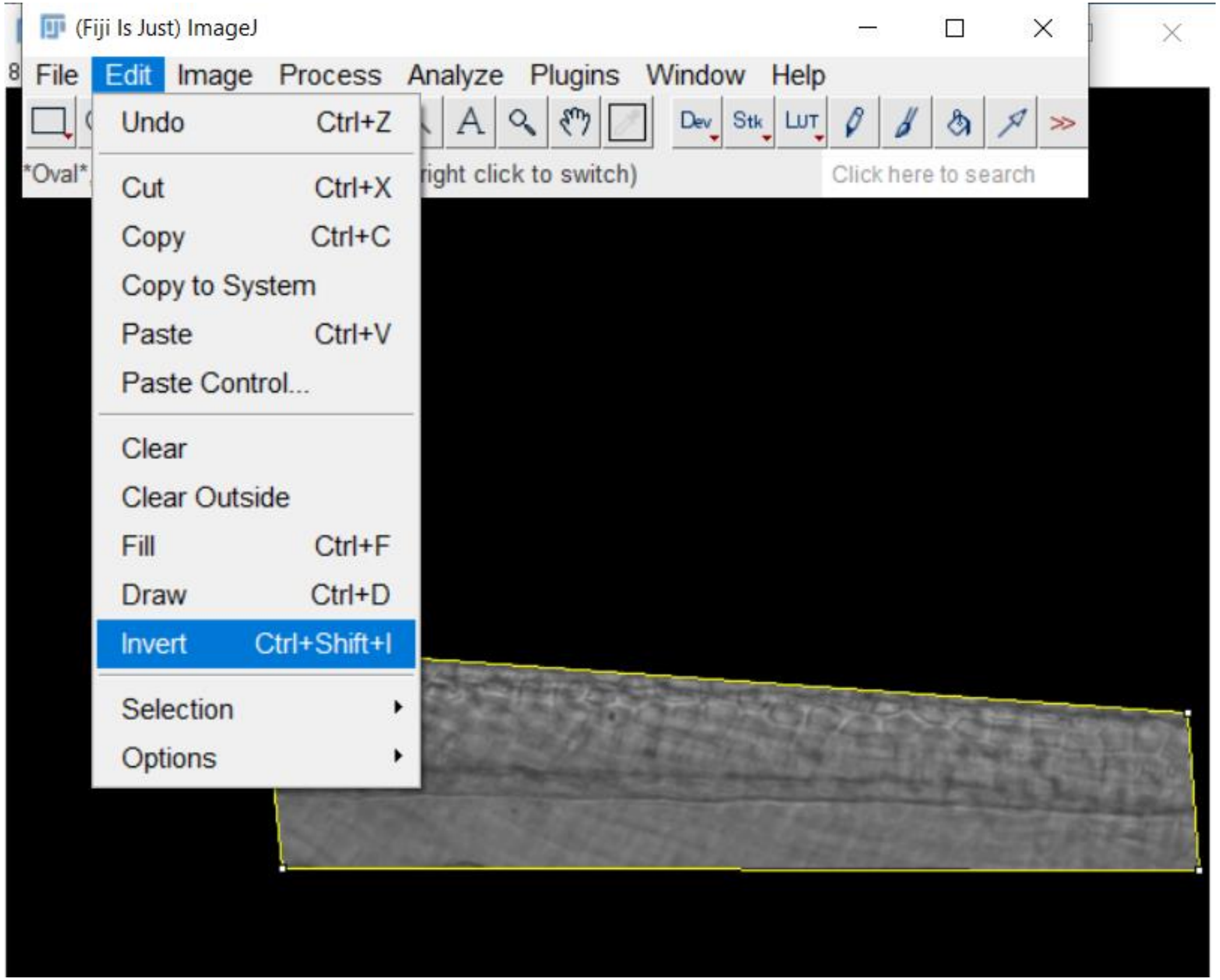

- Invert are used for creating a reversed image, similar to a photographic negative, of the entire image or selection

Invert Result

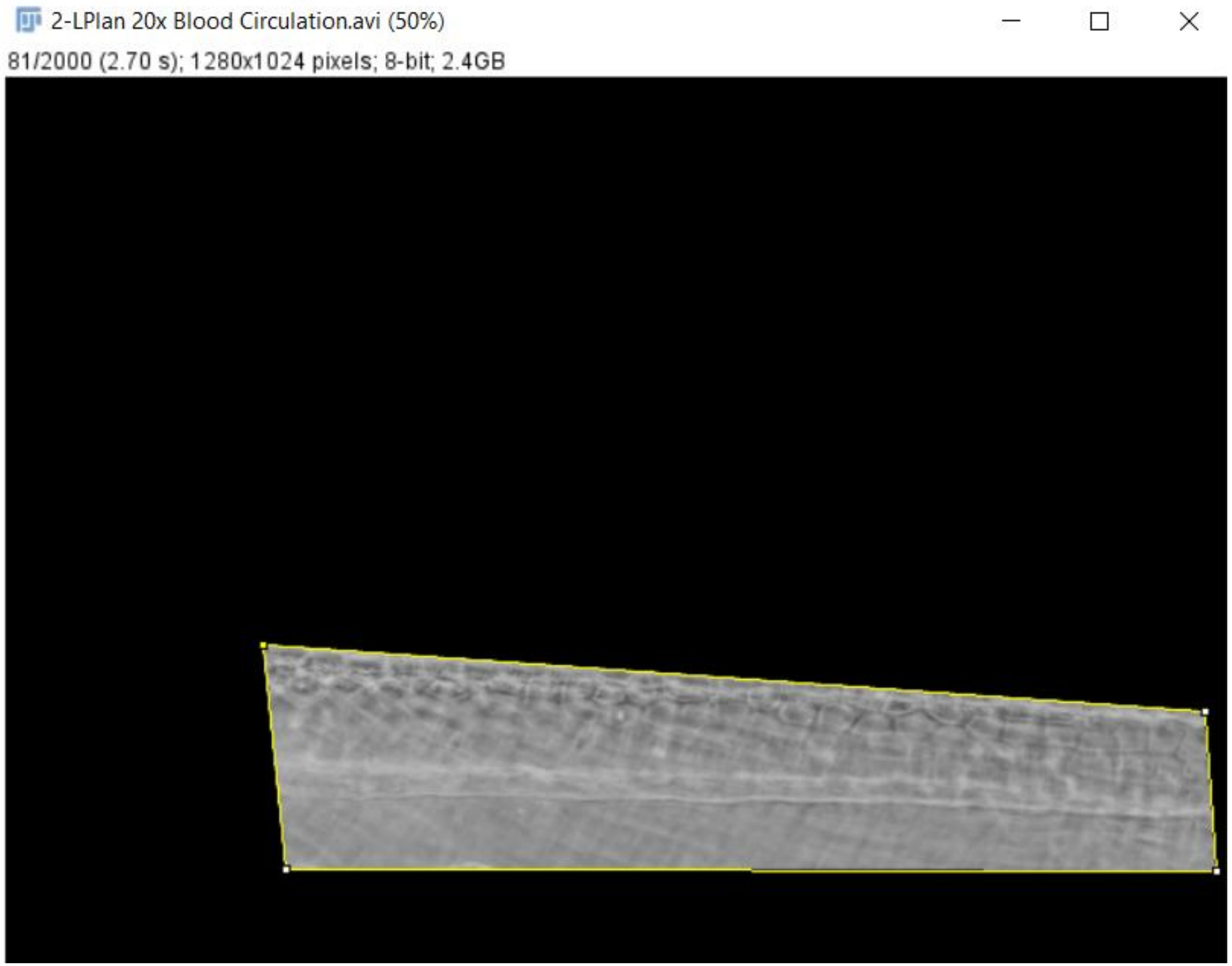

Select Stack Differences

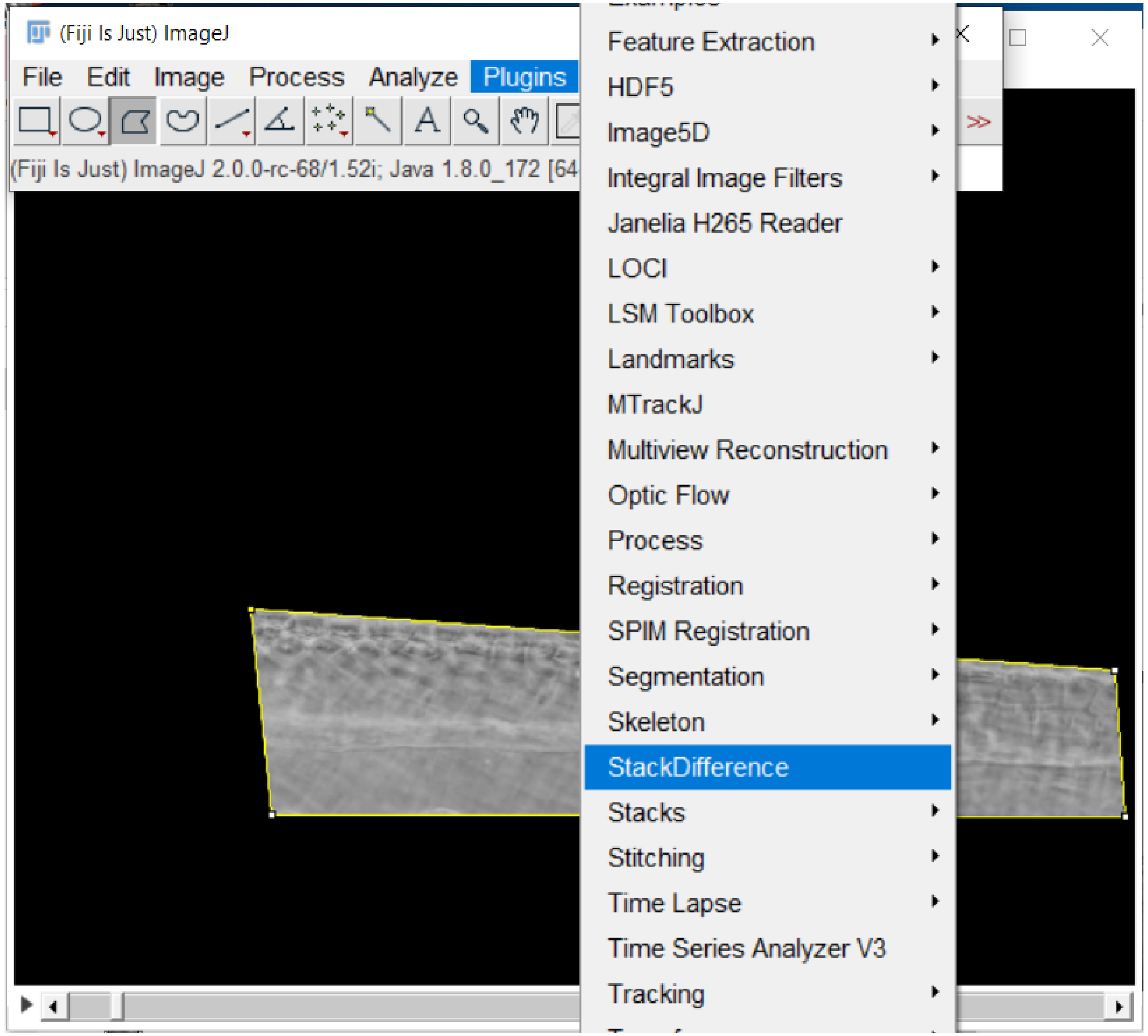

Plugins -> Stack Difference

- **Stack Differences** are used to detect the dynamic pixel changes between different frames

Stack Difference Result → Trackmate

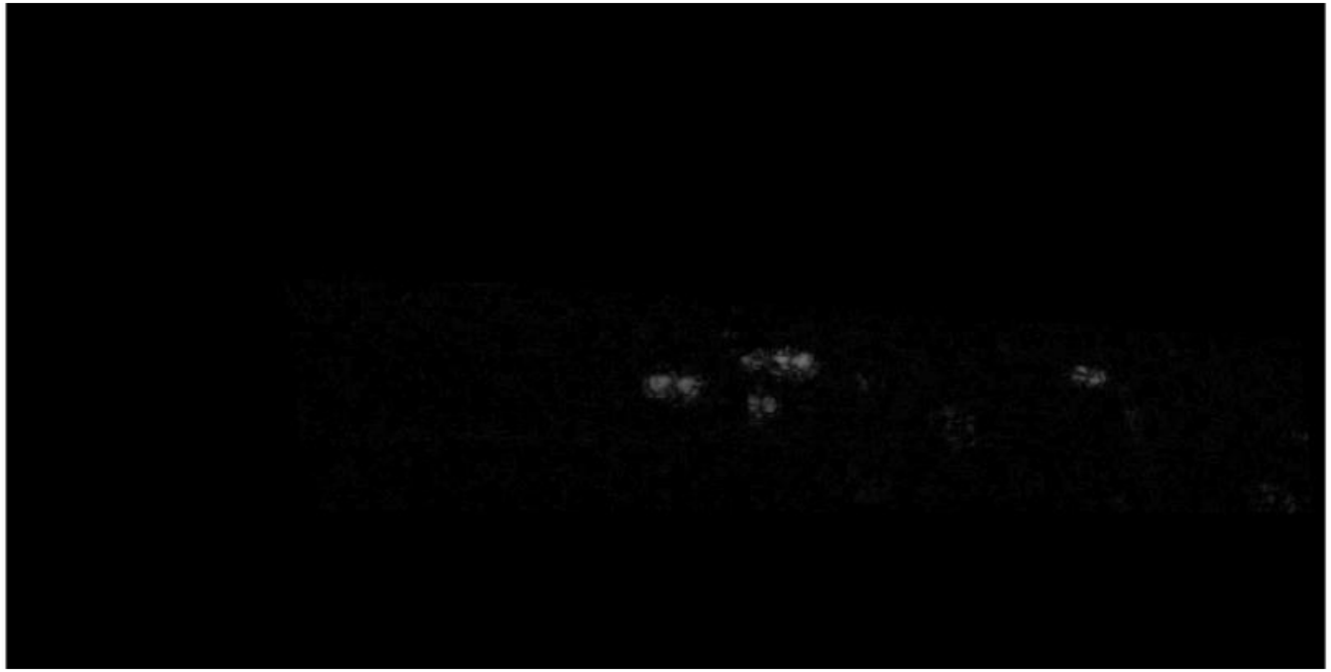

- Now the moving objects (blood cells) can be highlighted and the non-moving background can be ignored by using Stack difference plugin

TrackMate can deal with single particles, or spot-like objects. They are bright objects over a dark background for which the object contour shape is not important, but for which the main information can be extracted from the X,Y,Z coordinates over time

Select Trackmate

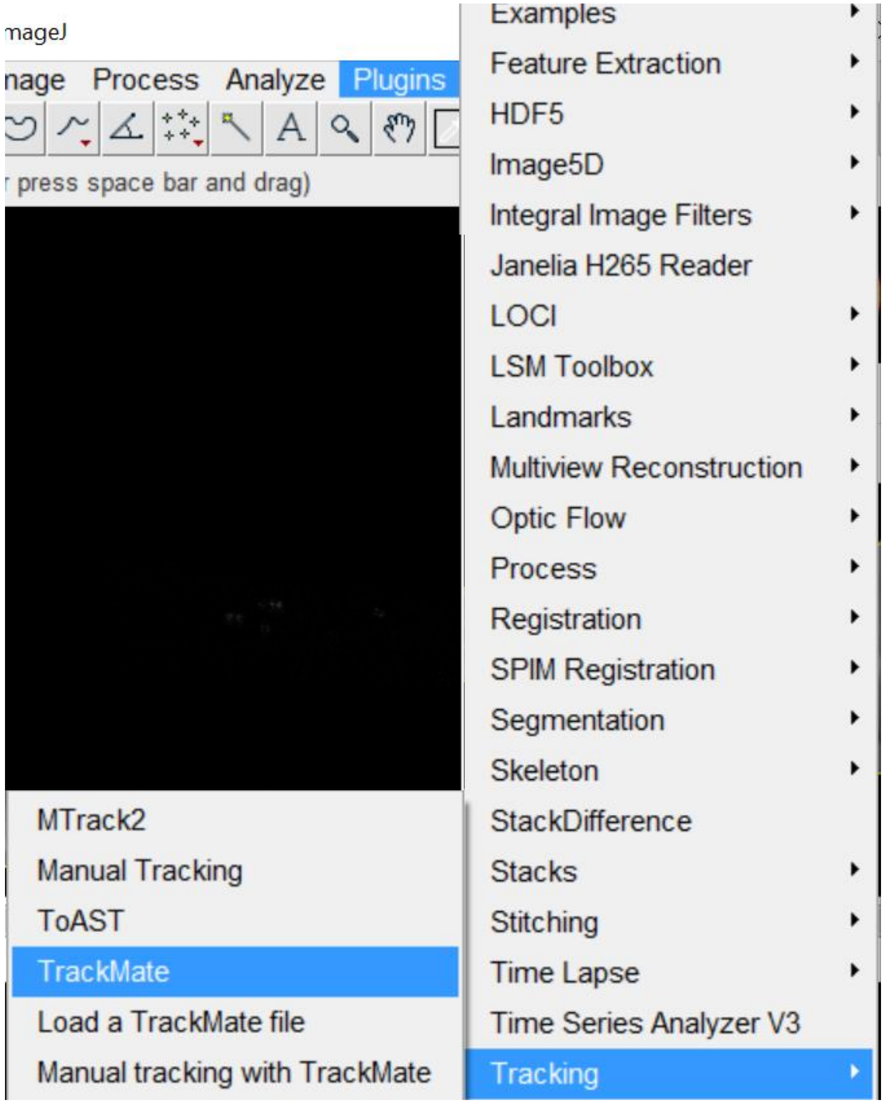

Plugins -> Tracking -> Trackmate

Check Spatial and Temporal Calibration

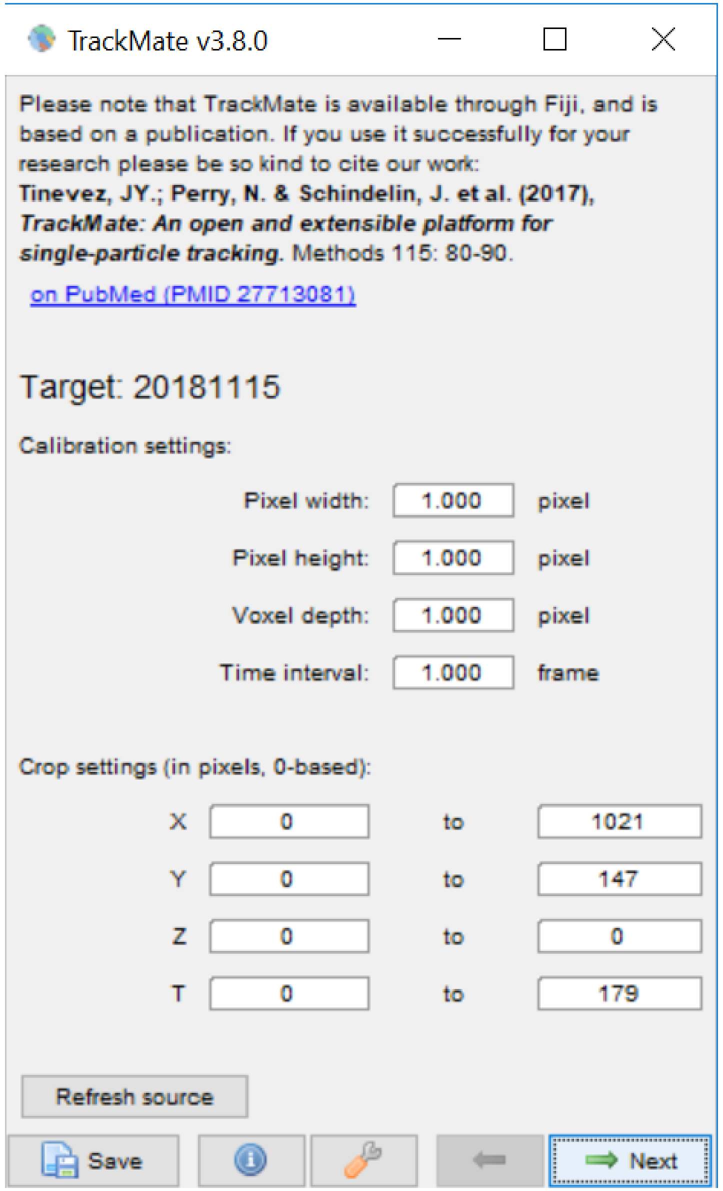

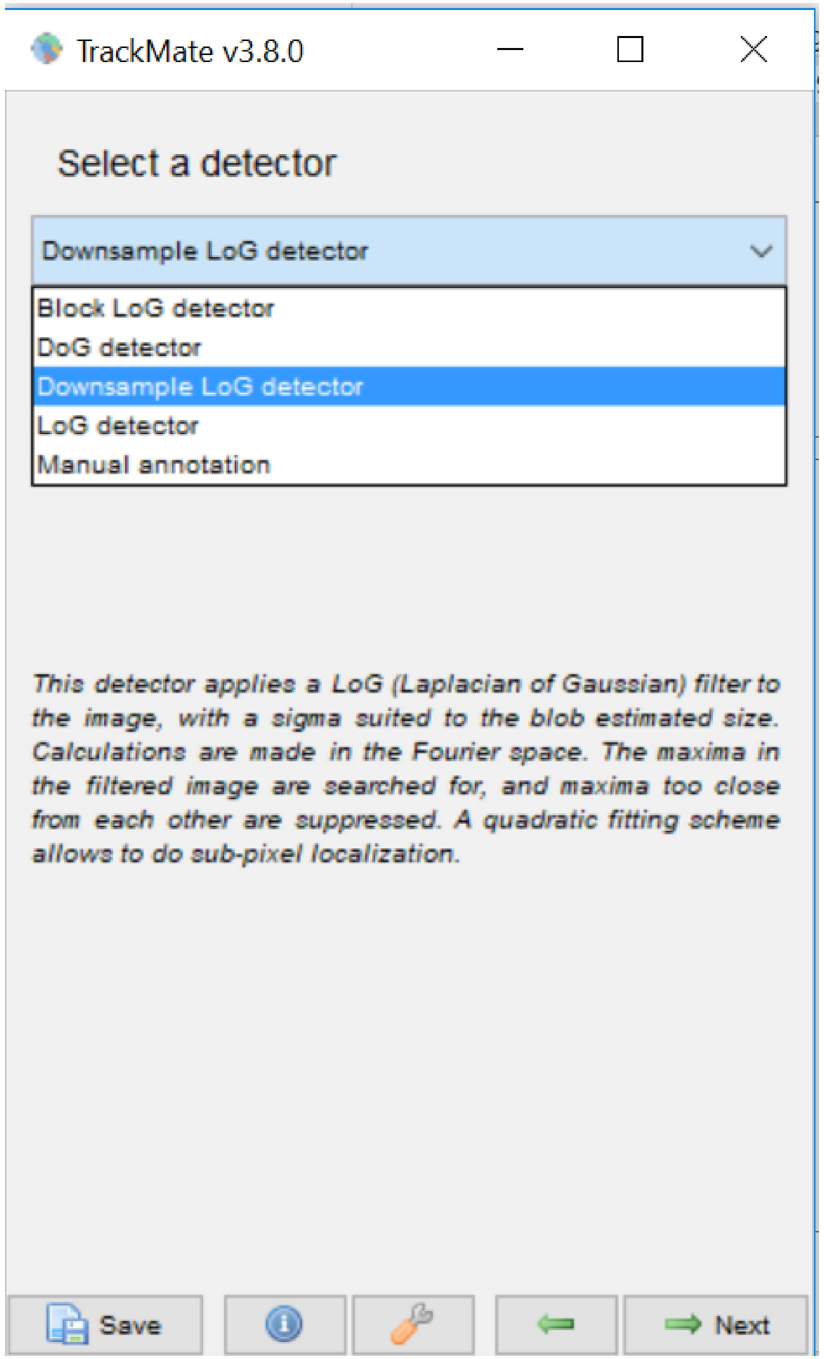

Object Detector

- LoG Detector -> optimal for object sizes 5 to 20 pixels
- Difference of Gaussian particle (DoG) Detector -> optimal for object sizes below 5 pixels
- Downsample LoG Detector -> optimal for object **sizes above 20 pixels**

In this study, we choose
Downsample LoG detector

Adjusting Parameter

- Estimated blob diameter
  Enter approximate object size based on the measurement
- Threshold
  Helping dealing with gigantic number of spots
- Preview

In this study, we choose **50** for **estimated blob diameter**, and **0.15** for **threshold**

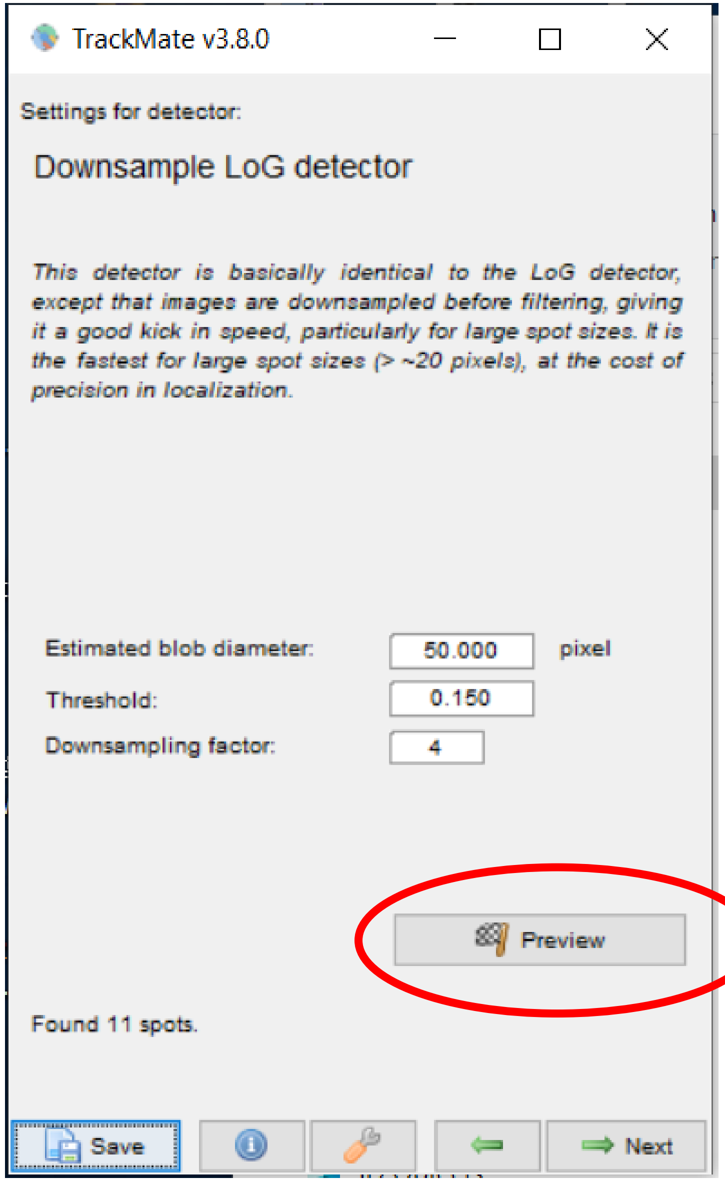

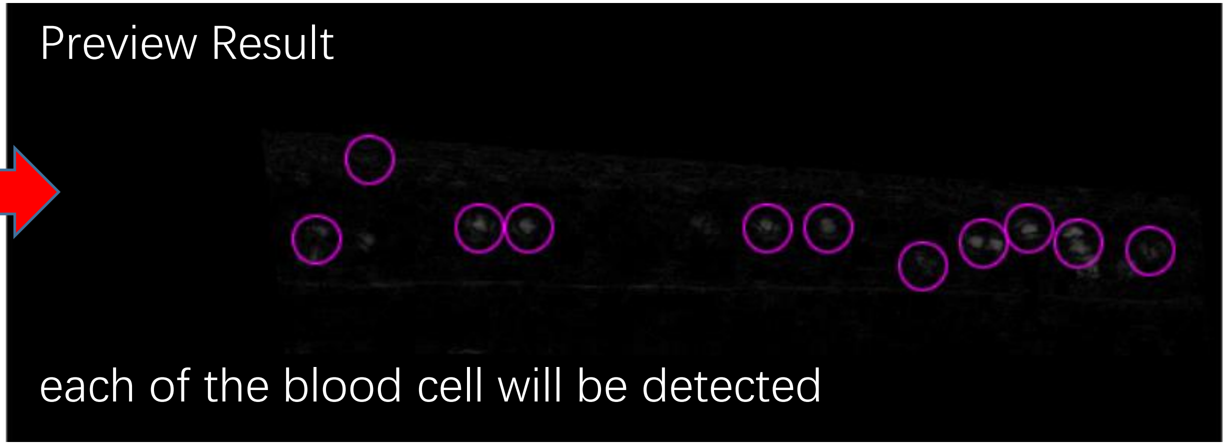

2

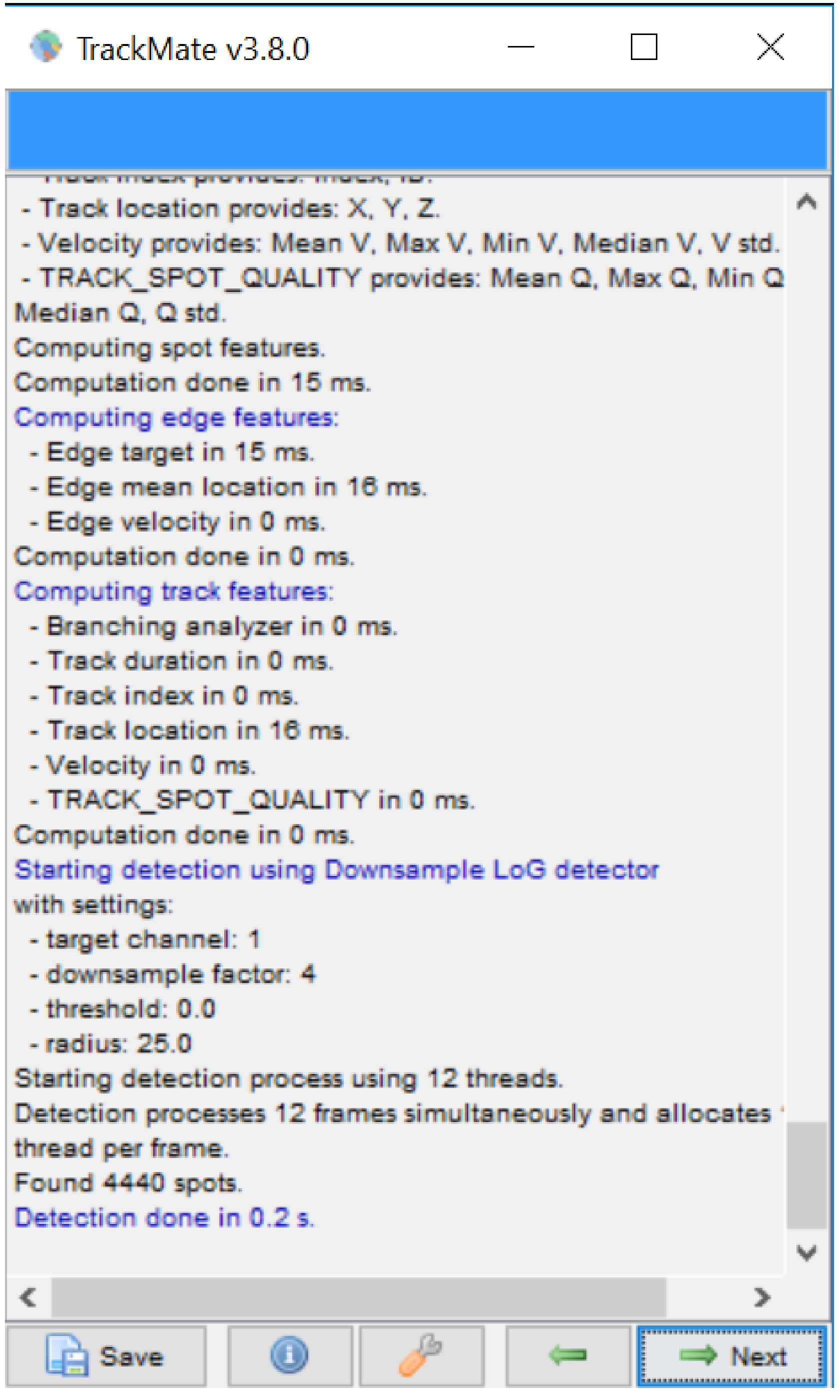

Detection Process

TrackMate takes advantage of multi-core computers, which seems to be the standard nowadays. It will segment one time-frame per core available. On computers with many cores, the progress bar will seems to move in a bulky way: if you have 16 cores, 16 time-points will be segmented at once, and it is likely that they will be finished approximately on the same time. So don't be worried if the progress bar does not move in the beginning for large images.

2

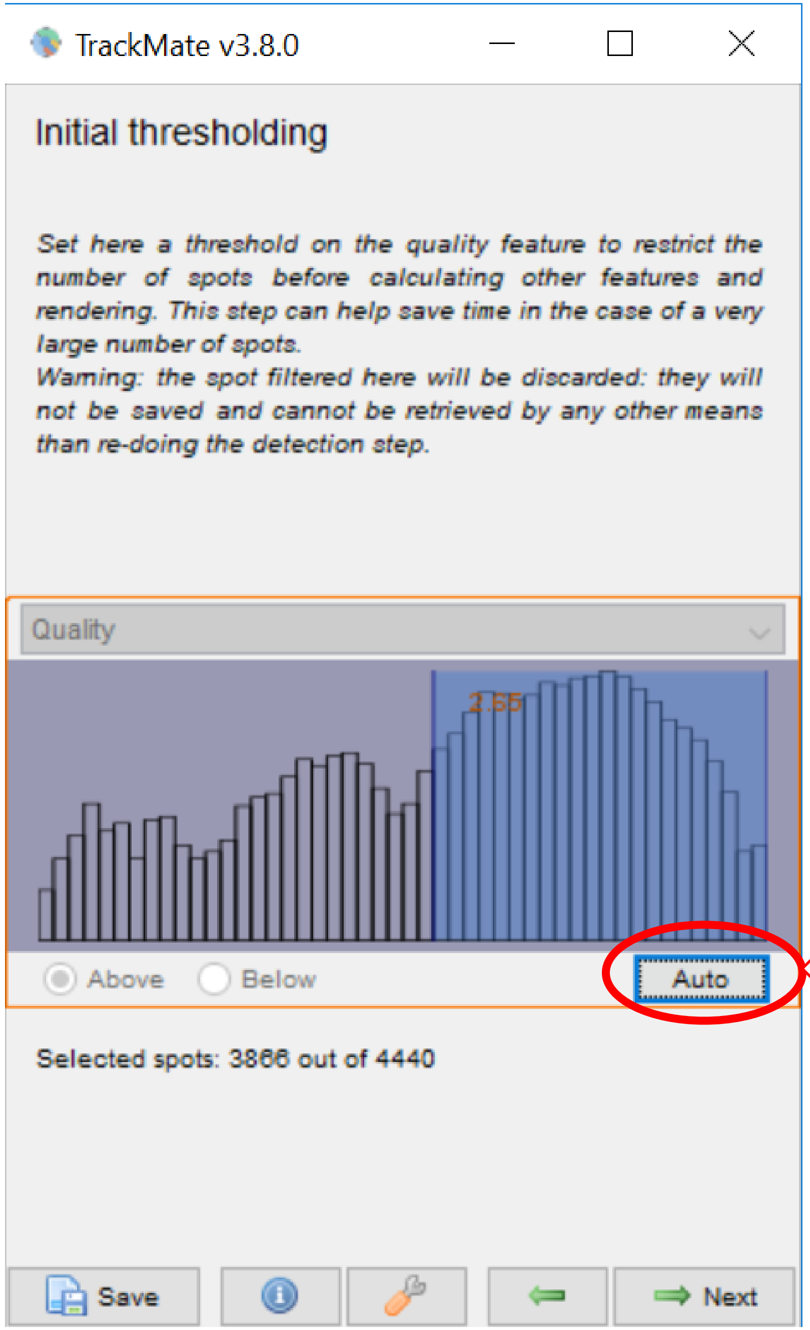

Choose Auto

Initial Object Filtering

- One of the filter that set by the segmenter and arbitrary measure, object with certain value will be discarded if not met the criteria

2

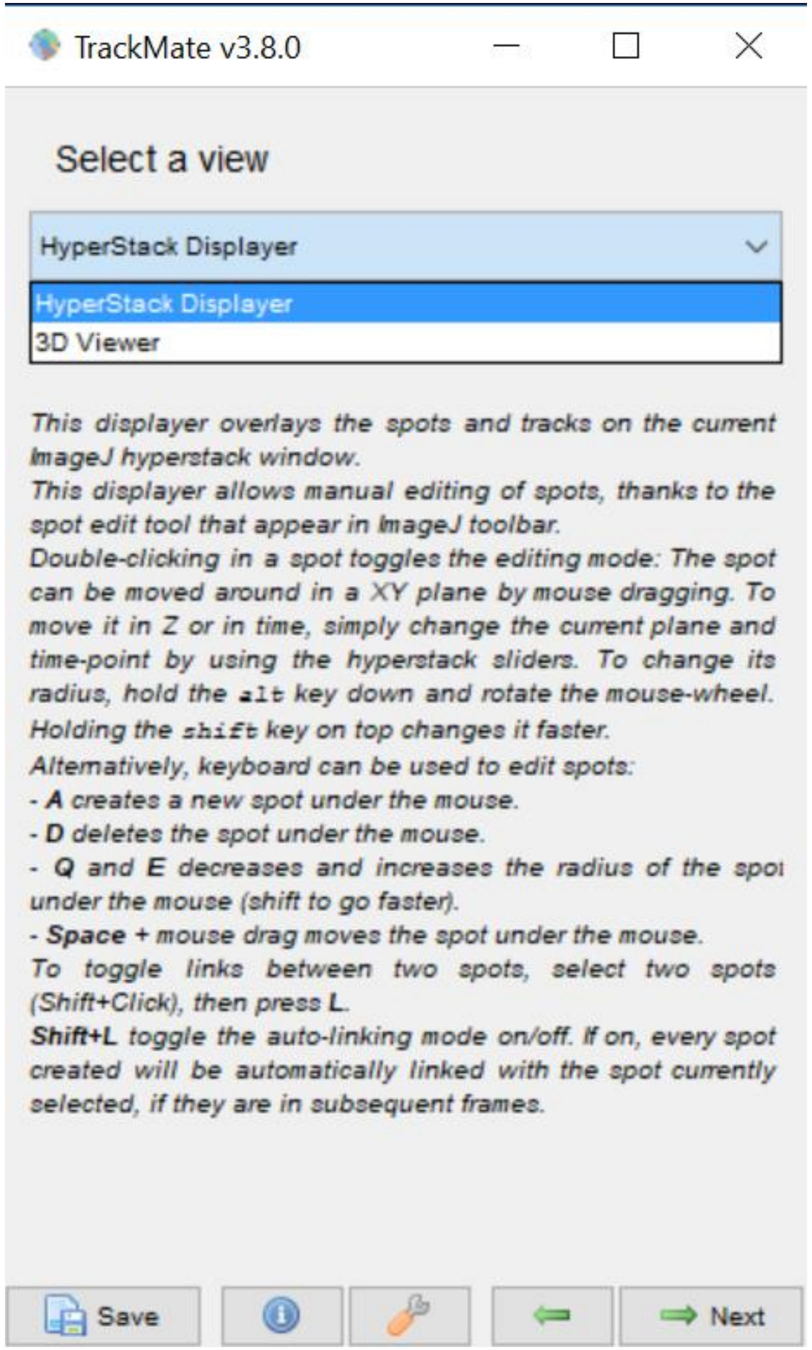

Selecting a View

- Hyperstack Displayer simply reuses ImageJ stack window and overlay the results non-destructively over image
- 3D viewer to calculate in 3D, in very complicated case

In this study, we choose **Hyperstack Displayer**

2

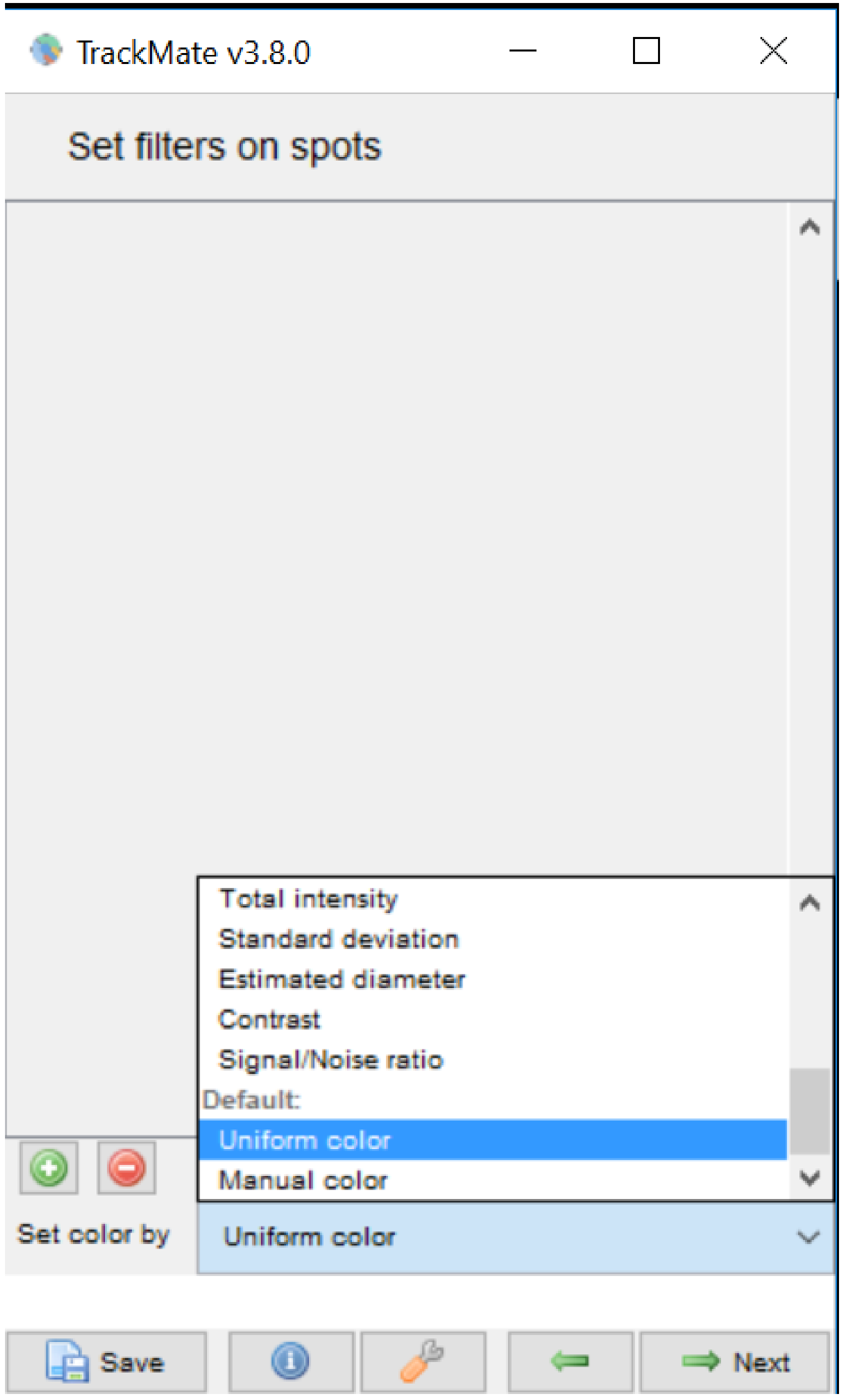

Object Filtering

- One of the filter feature to distinguish the object from noise
- Uniform color: based on the object color
- Estimated diameter: based on the size of the object

In this study, we choose
Uniform color

2

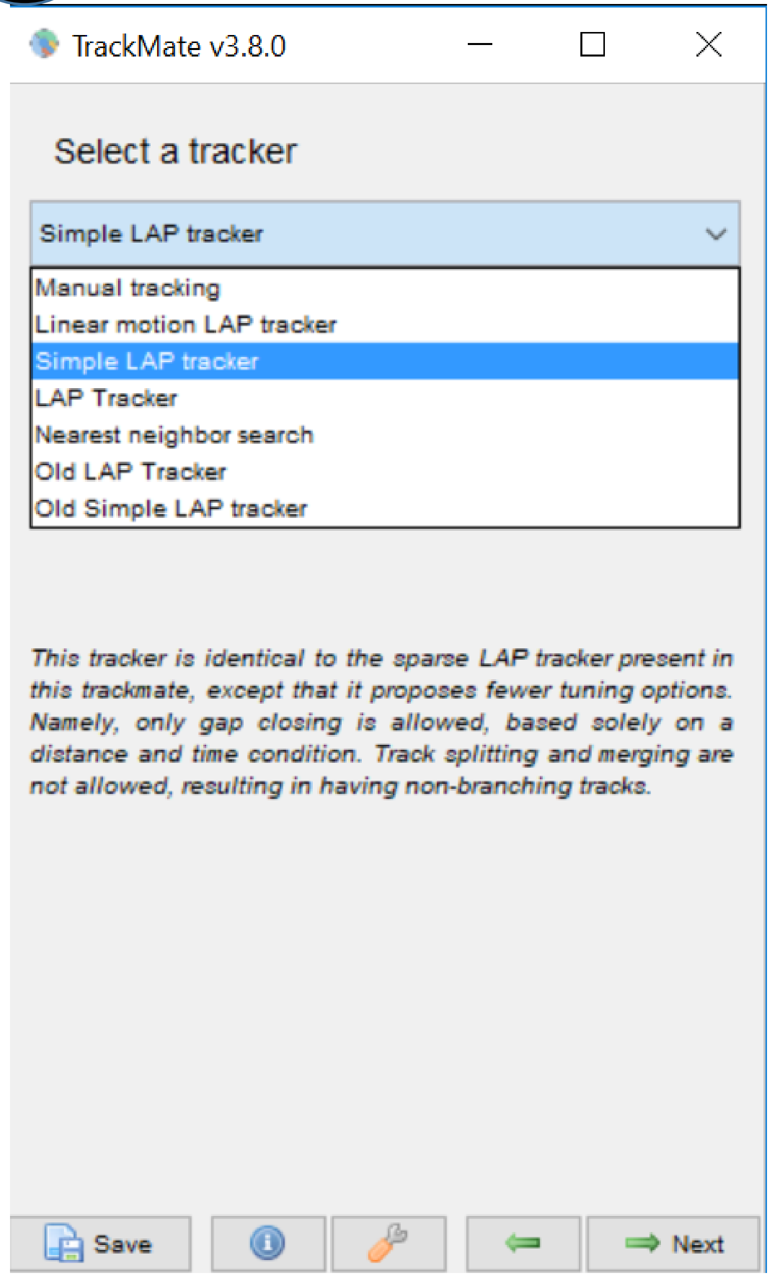

- Linear Assignment Problem (LAP)
- Simple LAP tracker
  -> Simplified version that has less settings, suitable for particle that did not merge nor divide
- Nearest neighbor search
  -> Each object in the frame is linked to another frame regardless of another object, work only with the most optimum one

In this study, we choose
simple LAP tracker

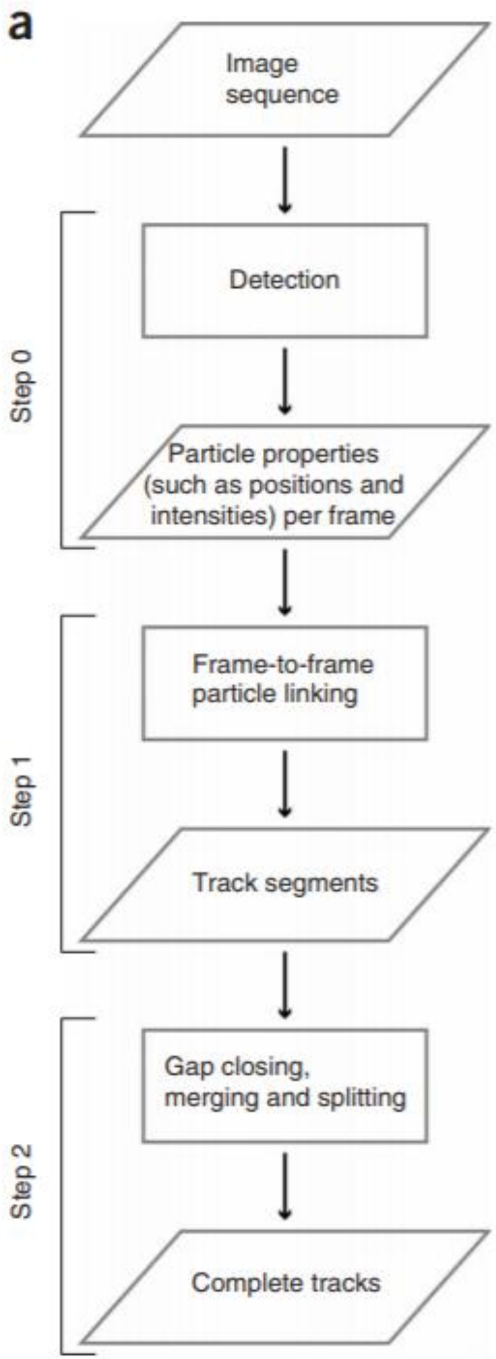

2

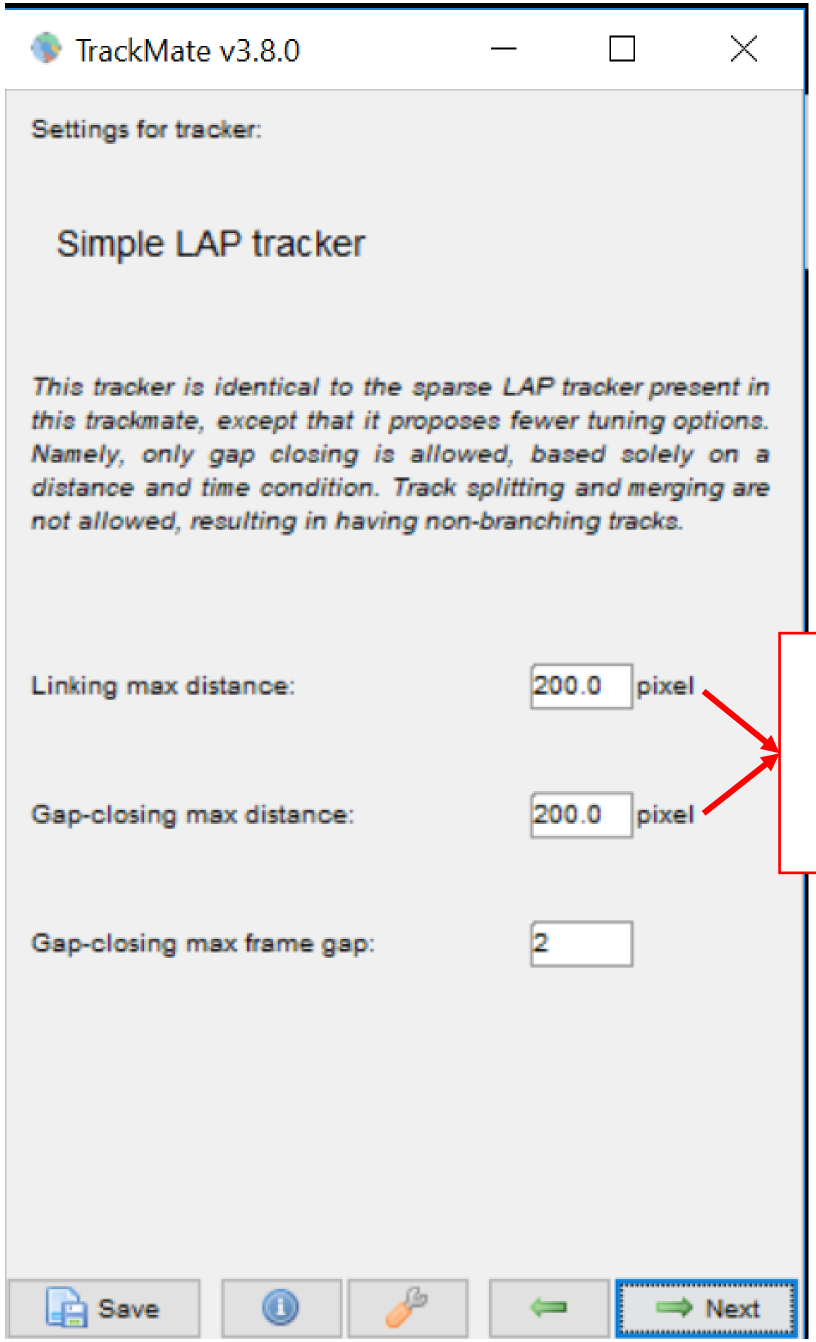

Filled with the max distance which measured by MTrackJ

- Linking max distance
  -> Maximum pixel of the object between two frames that allowed as the linking distance
- Gap-closing max frame gap
  -> Object would be detected if distance in the first frame is not greater than the next frame
- Gap-closing max frame gap
  -> Set maximal time interval between two objects to be detected

2

Tracking Process

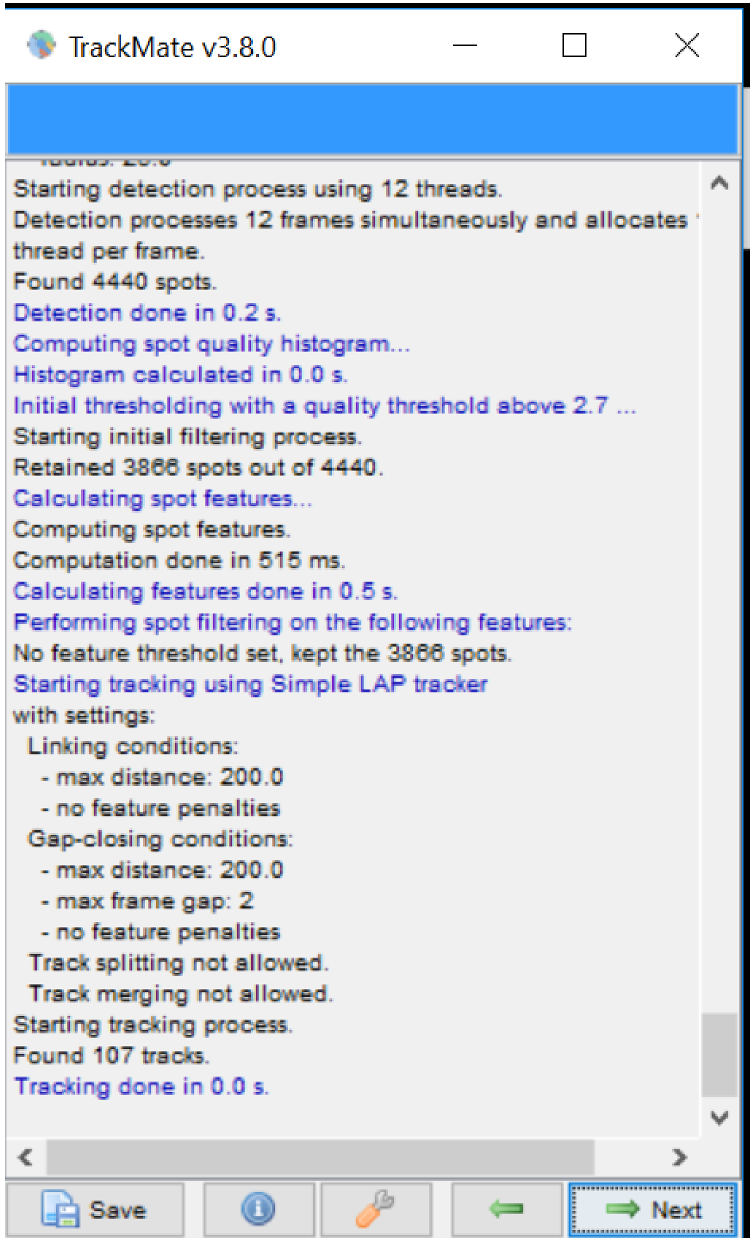

Set Filter on Tracks

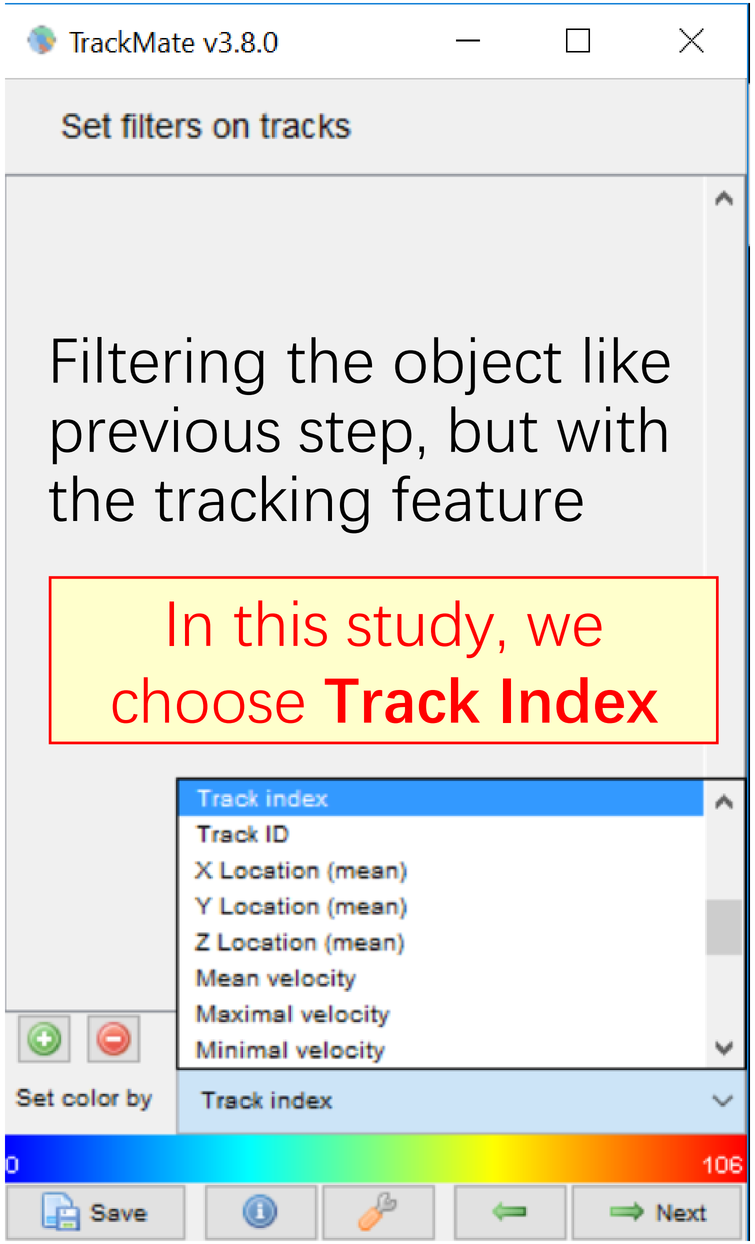

Filtering the object like previous step, but with the tracking feature

In this study, we choose **Track Index**

2

Select the Output Type

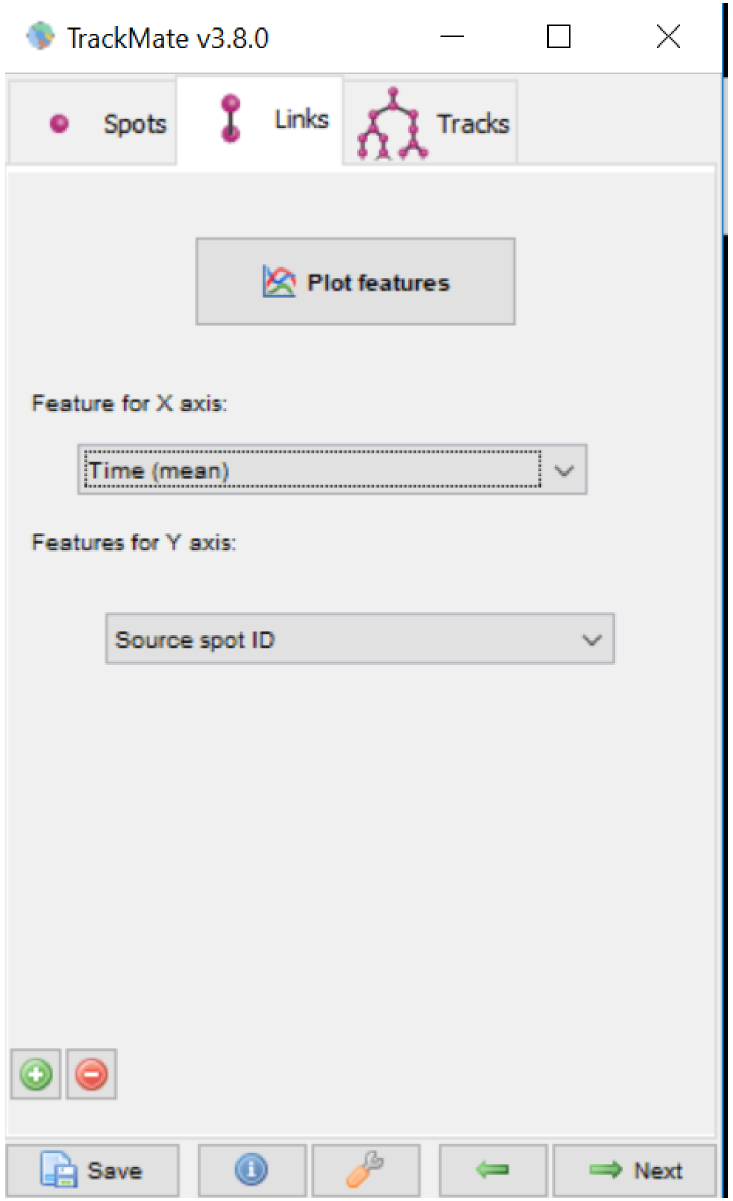

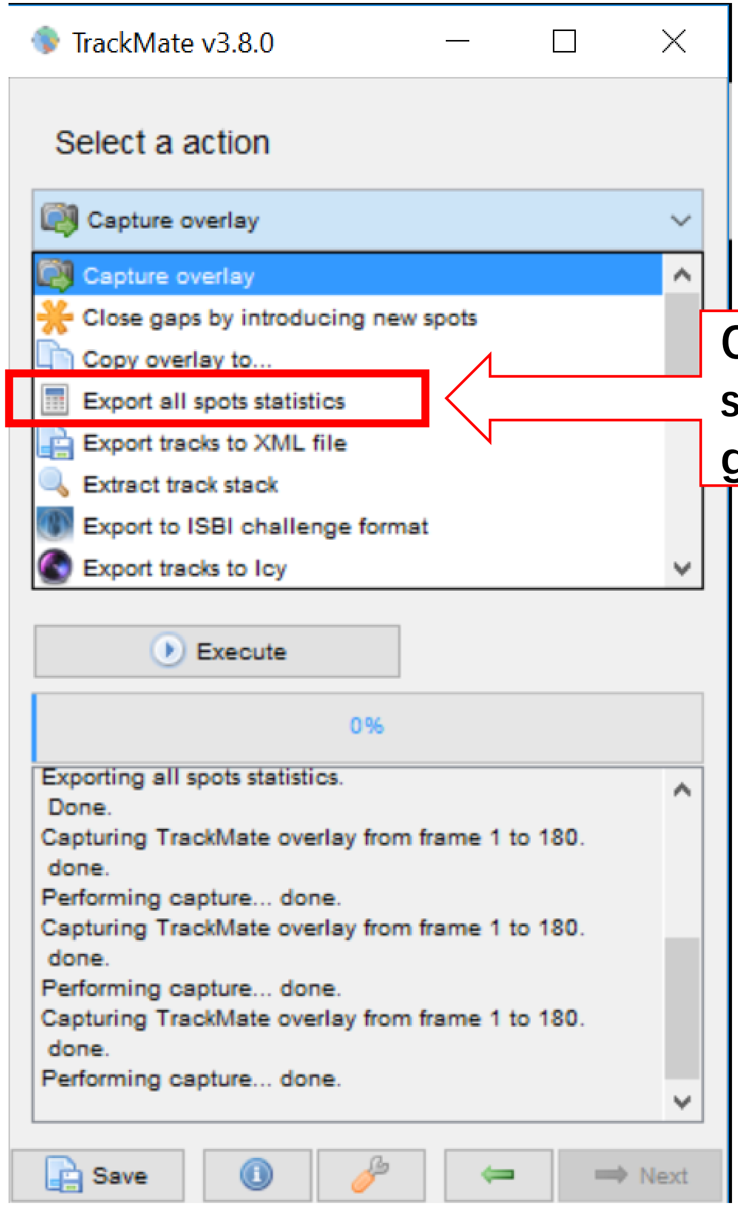

Choose export all spots statistics to get the raw data

All Spots statistics

File Edit Font

Label	ID	TRACK_ID	QUALITY	POSITION_X	POSITION_Y	POSITION_Z	POSITION_T	FRAME	RADIUS	VISIBILITY	MANUAL_COLOR	MEAN_INTENSITY	MEDIAN_INTENSITY
ID112	112	0	3.185	332	52	0	0	0	25	1	-10921639	115.399	119
ID104	104	1	3.291	532	40	0	0	0	25	1	-10921639	108.836	111
ID106	106	None	2.657	196	44	0	0	0	25	1	-10921639	98.600	99
ID107	107	2	3.514	448	44	0	0	0	25	1	-10921639	114.424	123
ID108	108	3	3.037	864	44	0	0	0	25	1	-10921639	117.477	119
ID109	109	4	3.606	412	48	0	0	0	25	1	-10921639	117.248	123
ID111	111	5	3.605	96	52	0	0	0	25	1	-10921639	104.836	107
ID232	232	1	3.196	536	44	0	1	1	25	1	-10921639	114.559	111
ID233	233	3	2.957	872	44	0	1	1	25	1	-10921639	117.175	118
ID236	236	4	3.660	424	48	0	1	1	25	1	-10921639	118.972	124
ID238	238	5	3.494	100	52	0	1	1	25	1	-10921639	104.622	107
ID231	231	2	3.404	472	44	0	1	1	25	1	-10921639	114.766	121
ID239	239	0	3.045	332	52	0	1	1	25	1	-10921639	114.286	118
ID81	81	3	3.193	876	40	0	2	2	25	1	-10921639	116.542	118
ID82	82	6	2.684	224	44	0	2	2	25	1	-10921639	101.080	104
ID83	83	2	3.836	428	44	0	2	2	25	1	-10921639	114.506	124
ID84	84	7	2.737	176	48	0	2	2	25	1	-10921639	101.690	103
ID85	85	0	2.737	280	48	0	2	2	25	1	-10921639	107.899	110
ID88	88	5	3.622	104	52	0	2	2	25	1	-10921639	105.211	106
ID89	89	4	3.176	340	52	0	2	2	25	1	-10921639	115.724	119
ID79	79	1	3.298	548	40	0	2	2	25	1	-10921639	110.760	112
ID324	324	1	3.263	556	40	0	3	3	25	1	-10921639	112.419	113
ID326	326	3	3.226	884	40	0	3	3	25	1	-10921639	117.547	119
ID327	327	2	3.957	436	44	0	3	3	25	1	-10921639	116.138	125
ID329	329	7	2.873	176	48	0	3	3	25	1	-10921639	102.541	103
ID330	330	6	2.791	240	48	0	3	3	25	1	-10921639	105.401	107
ID331	331	0	2.755	284	48	0	3	3	25	1	-10921639	108.595	111
ID332	332	4	3.185	336	48	0	3	3	25	1	-10921639	110.217	117
[illegible]	[illegible]	[illegible]	[illegible]	[illegible]	[illegible]	[illegible]	[illegible]	[illegible]	[illegible]	[illegible]	[illegible]	[illegible]	[illegible]

2

Copy the Raw Data into Excel

	A	B	C	D	E	F
1	Label	TrackID	X	Y	Frame	
2	ID269900	0	532	76	0	
3	ID269972	0	540	76	1	
4	ID269929	0	556	80	2	
5	ID270135	0	572	80	3	
6	ID270014	0	584	80	4	
7	ID270077	0	592	80	5	
8	ID270046	0	596	80	6	
9	ID269956	0	604	84	7	
10	ID270190	0	612	84	8	
11	ID270163	0	632	84	9	
12	ID269909	1	28	120	0	
13	ID269988	1	32	120	1	
14	ID269935	1	40	120	2	
15	ID270141	1	44	120	3	
16	ID270024	1	56	120	4	
17	ID270086	1	64	120	5	
18	ID270054	1	76	124	6	
19	ID269968	1	84	124	7	
20	ID270196	1	80	124	8	
21	ID270169	1	144	124	9	
22	ID270227	1	140	124	10	

Sheet1 Sheet2 Sheet3

A. Label
Show each detected tracking point ID

B. TrackID
Show each detected tracking point ID based on the same object

C. X
Show X position of the object in pixel

D. Y
Show Y position of the object in pixel

E. Frame
Show each detected tracking point frame number

2

Data Analysis

Individual blood cell ID

	A	B	C	D	E	F	G	H	I	J	K	L	M	N	O	P	Q
1	Label	TrackID	X	X2-X1	Y	Y2-Y1	Distance (	0	Total Indiv	Average Ir	0		Average Ir	Average I	Average S	MIN	MAX
2	ID269900	0	532	8	76	0	8	8	0	0	1	nan	10.14367	1.73693	312.6473	30.82192	1439.09
3	ID269972	0	540	16	76	4	16.49242	24.49242	0	0	2	nan	7.11608	1.218507	219.3312		
4	ID269929	0	556	16	80	0	16	40.49242	0	0	3	nan	3.763939	0.64451	116.0118		
5	ID270135	0	572	12	80	0	12	52.49242	0	0	4	nan	6.094427	1.043566	187.8419		
6	ID270014	0	584	8	80	0	8	60.49242	0	0	5	nan	9.670738	1.655948	298.0707		
7	ID270077	0	592	4	80	0	4	64.49242	0	0	6	nan	8	1.369863	246.5753		
8	ID270046	0	596	8	80	4	8.944272	73.43669	0	0	7	nan	8.912316	1.526082	274.6947		
9	ID269956	0	604	8	84	0	8	81.43669	0	0	8	nan	9.129362	1.563247	281.3844		
10	ID270190	0	612	20	84	0	20	101.4367	0	0	9	nan	12.17984	2.085588	375.4059		
11	ID270163	0	632	nan	84	nan	nan	0	101.4367	10.14367	0	10.14367	6.157379	1.054346	189.7822		
12	ID269909	1	28	4	120	0	4	4	0	0	1	nan	9.029157	1.546088	278.2959		

D. X2-X1 -> subtraction of the next and previous time point of X axis

F. Y2-Y1 -> subtraction of the next and previous time point of Y axis

G. **Distance (pixel)** -> $\sqrt{(\boldsymbol{X2}-\boldsymbol{X1})^2+(\boldsymbol{Y2}-\boldsymbol{Y1})^2}$

I. Total Individual Distance (pixel) -> summary of each individual distance

J. Average Individual Distance (pixel) -> summary of each individual distance divided by detected track number

N. Average Individual Distance (μm) -> conversion from pixel into μm by scale

O. **Average Speed (μm/sec)** -> average individual distance (μm) multiply by recording speed (in this case 200fps)

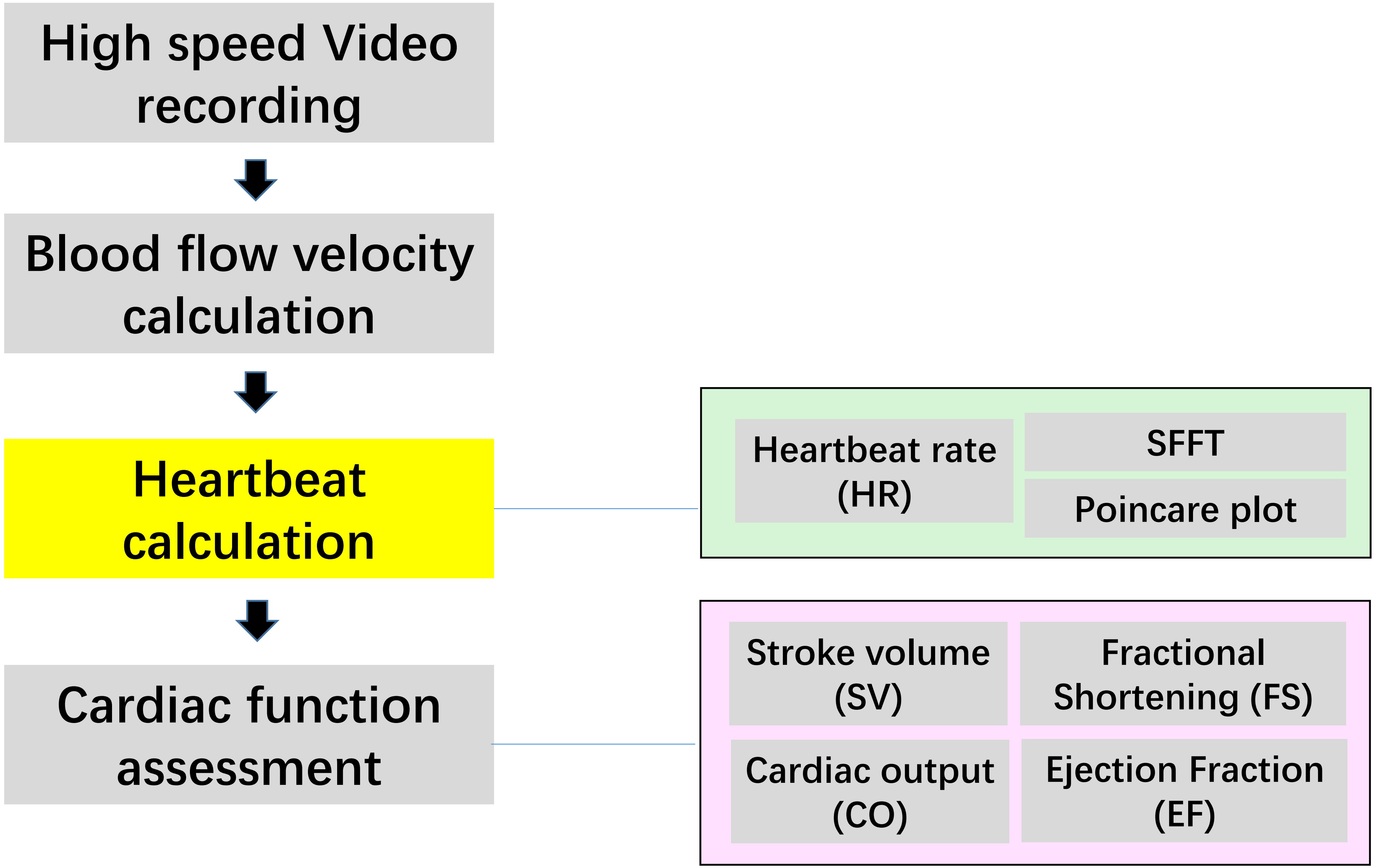
High speed Video recording
Blood flow velocity calculation
Heartbeat calculation
Cardiac function assessment
Heartbeat rate (HR)
SFFT
Poincare plot
Stroke volume (SV)
Fractional Shortening (FS)
Cardiac output (CO)
Ejection Fraction (EF)

Open the Video File with ImageJ

- Click File + Open to Select Video File
- Select the VirtualDub converted video
- In AVI Reader of ImageJ, choose the frames selected and check Use Virtual Stack

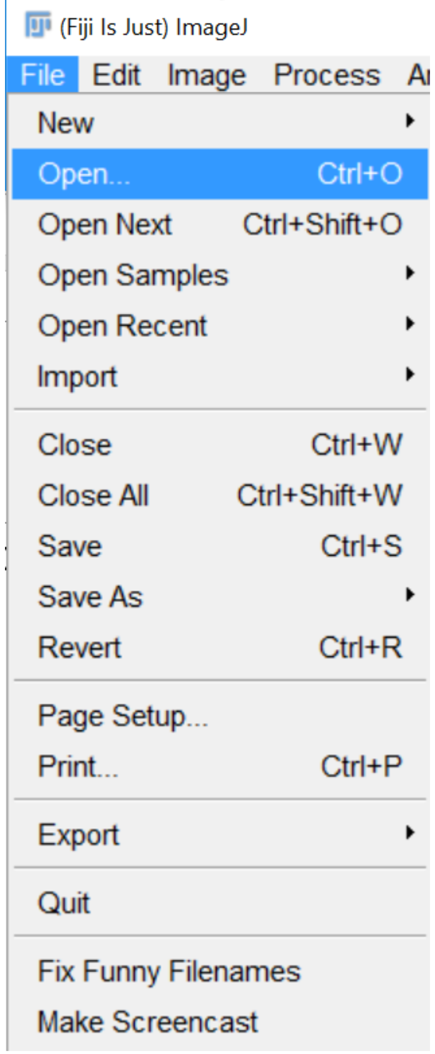

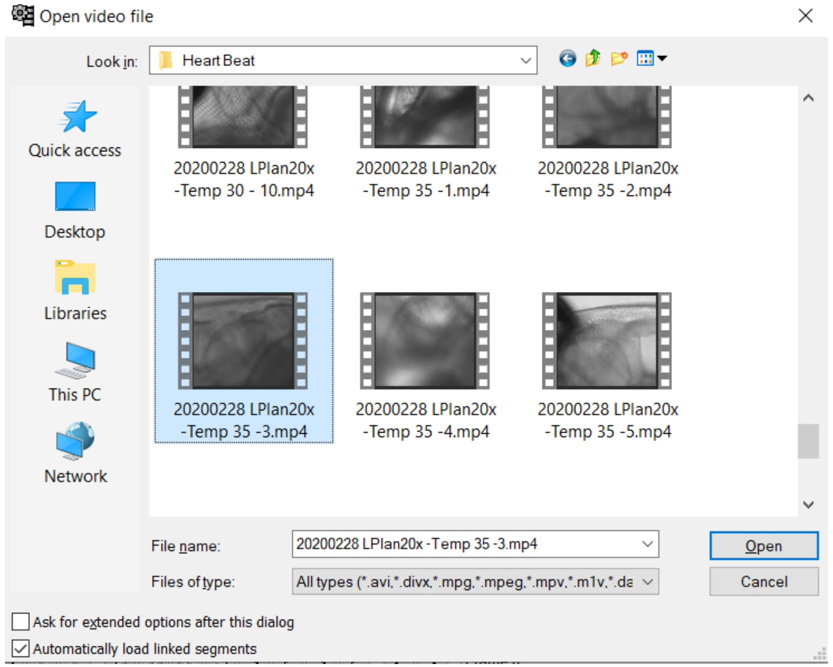

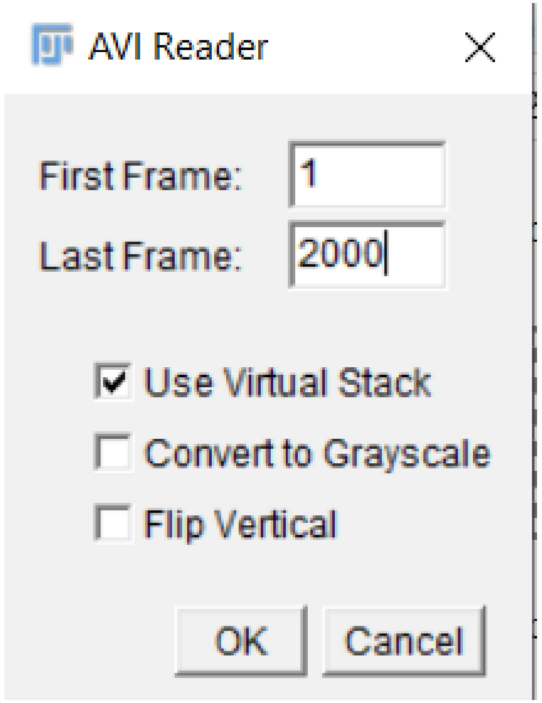

Select Region of Interesting (ROI) in ImageJ

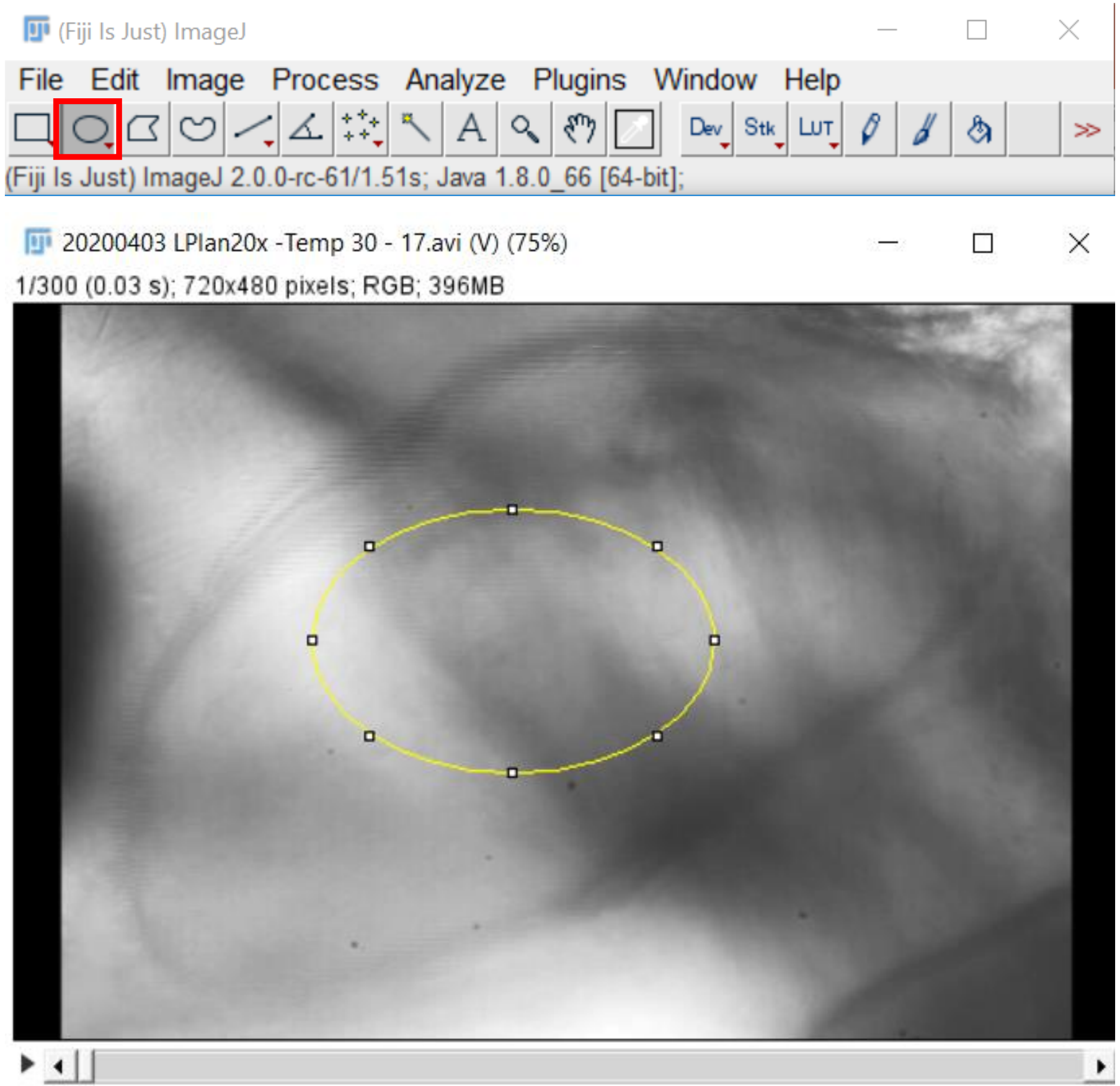

- Use *Oval* brush selection to choose ROI in specific heart area
- **The ROI can be specified in middle or the entire heart region to measure the beating rate**

Use Time Series Analyzer Plugin

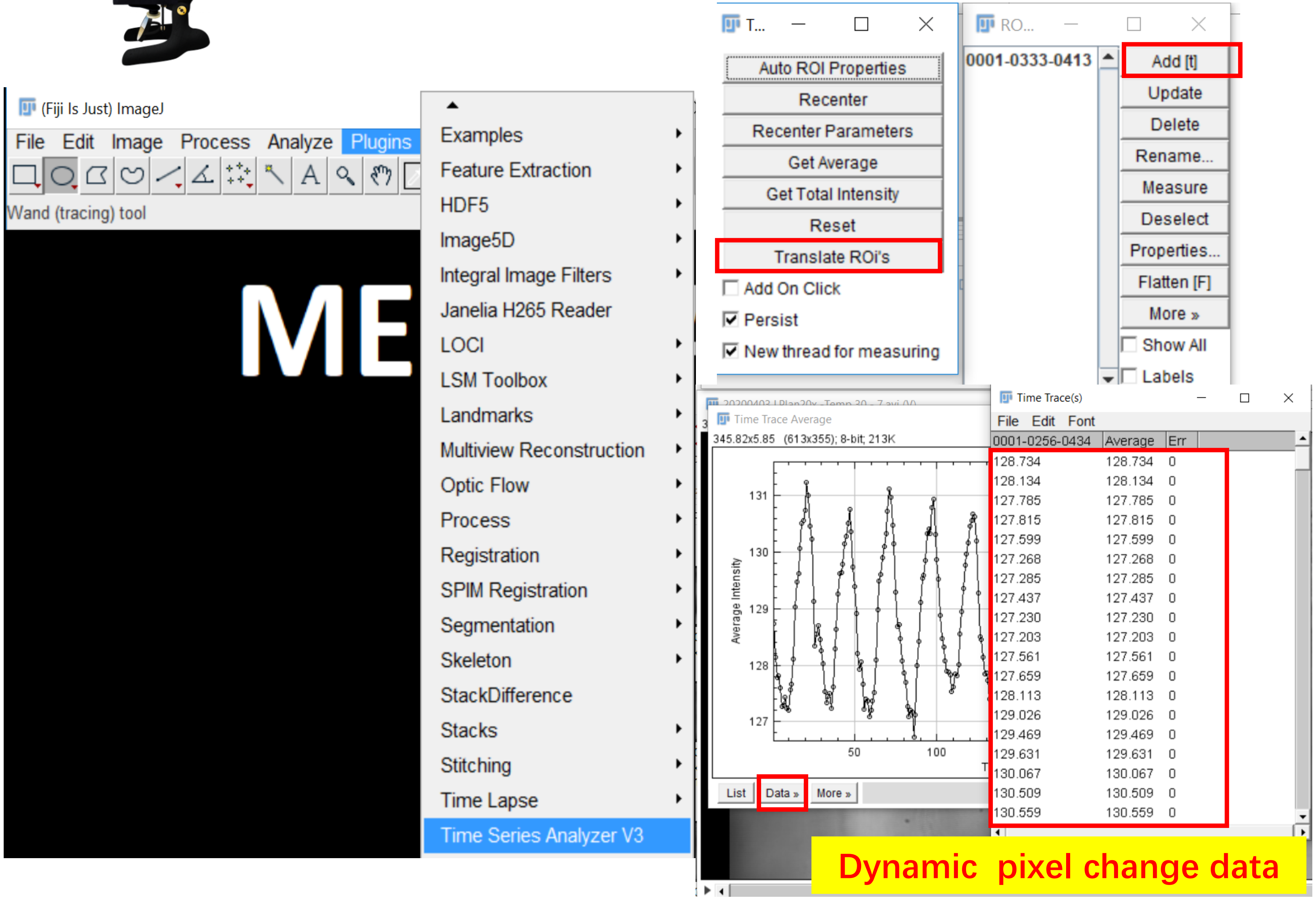

- Choose Time Series Analyzer V3 plugin
- Click "Add" Button to choose ROI
- Click "Get Average" to get the peak and data
- Click "Save" in the peak window to keep the data for further analysis
- After this analysis, the **dynamic pixel change data** can be extracted into Excel

Input The Data Into Origin 9.1

Book1 *

	A(X)	B(Y)	C(Y)	D(Y)	E(Y)	F(Y)
Long Name						
Units						
Comments	Time	1	2	3	4	5
F(x)=						
1	0.03333	95.418	113.231	123.853	125.36	116.915
2	0.06667	95.735	113.382	123.466	124.81	116.997
3	0.1	96.001	113.11	123.612	125.33	117.797
4	0.13333	96.015	112.853	124.09	125.395	118.947
5	0.16667	96.391	113.139	124.024	125.278	120.078
6	0.2	96.132	112.628	124.068	124.999	120.606
7	0.23333	96.674	111.905	124.087	125.542	121.759
8	0.26667	96.913	112.209	124.482	124.459	122.121
9	0.3	97.11	112.159	124.262	123.188	121.898
10	0.33333	96.765	111.423	124.758	123.228	121.858
11	0.36667	97.316	111.112	125.099	122.619	122.268
12	0.4	97.32	111.157	124.541	121.767	121.987
13	0.43333	97.187	110.714	125.023	121.955	122.143
14	0.46667	97.137	111.152	125.328	121.923	122.448
15	0.5	97.279	112.046	125.277	121.473	120.55
16	0.53333	97.037	112.248	124.549	121.576	119.36
17	0.56667	97.424	111.935	125.328	122.063	118.898
18	0.6	97.412	112.768	125.534	121.897	118.055
19	0.63333	97.508	113.159	125.341	123.013	117.333
20	0.66667	97.227	113.377	125.407	124.342	116.872
21	0.7	97.682	113.282	125.597	124.545	116.705
22	0.73333	97.595	113.859	125.282	124.982	116.042
23	0.76667	97.546	113.963	125.615	125.496	116.383
24	0.8	97.313	114.404	125.539	125.71	116.585
25	0.83333	97.714	114.552	125.677	125.345	117.444
26	0.86667	97.319	114.457	125.233	125.559	117.753
27	0.9	97.573	113.977	125.721	126.398	119.093
28	0.93333	97.806	114.677	125.721	126.427	120.709
29	0.96667	97.819	114.875	125.041	126.949	121.684
30	1	97.428	114.845	125.3	127.211	122.484

Sheet1

- The data matrix is directly copy from Excel and paste to Origin 9.1

Make Graphic by using Origin 9.1

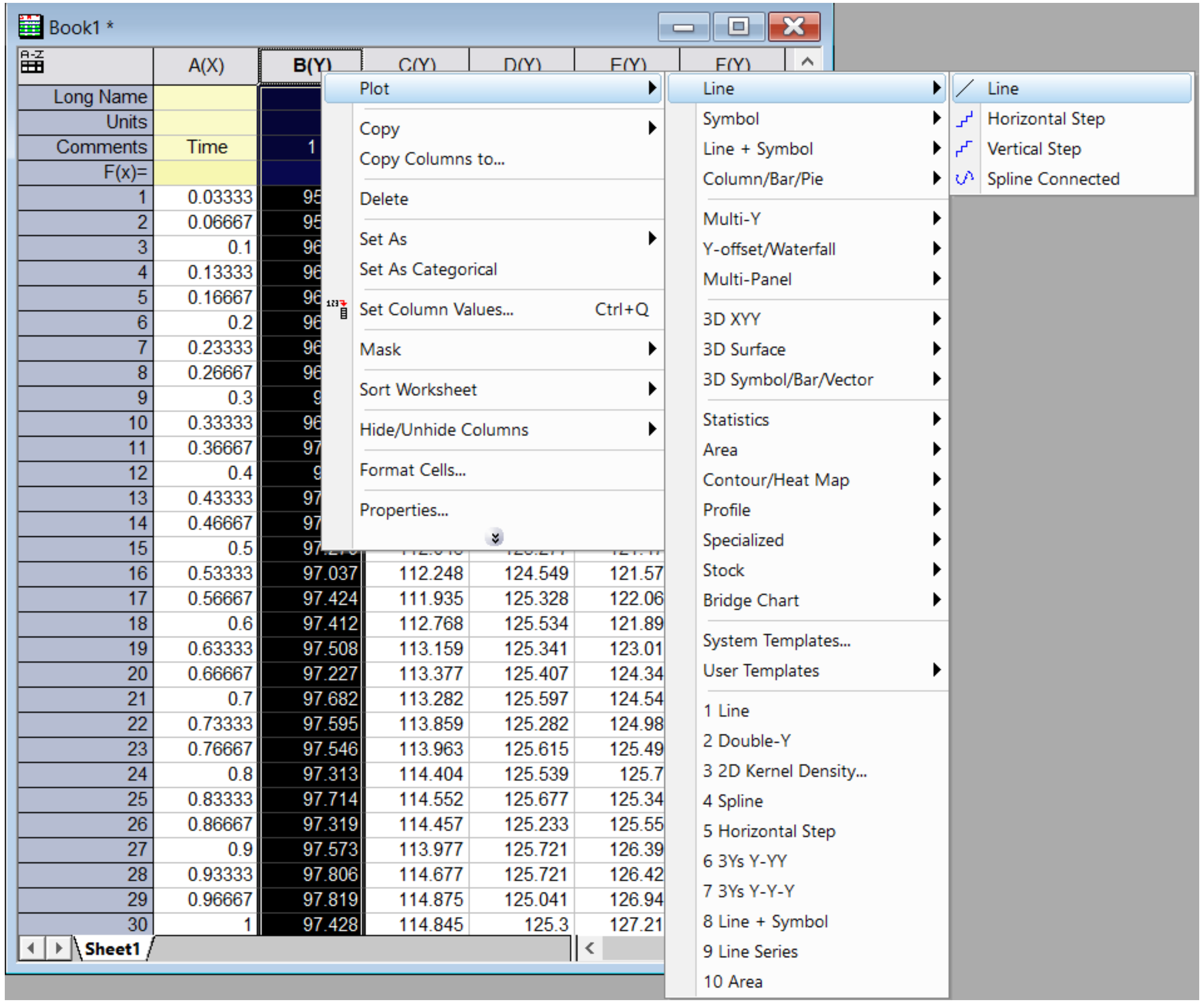

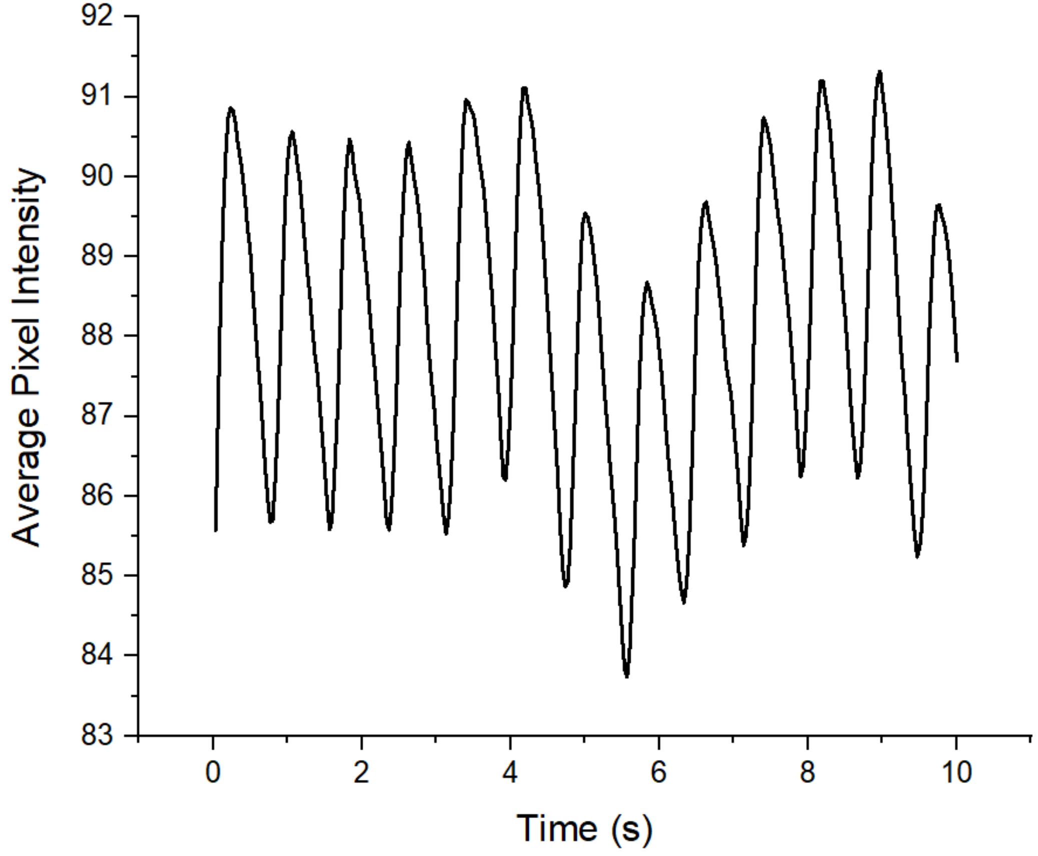

Use Peak Analyzer to detect the peak interval

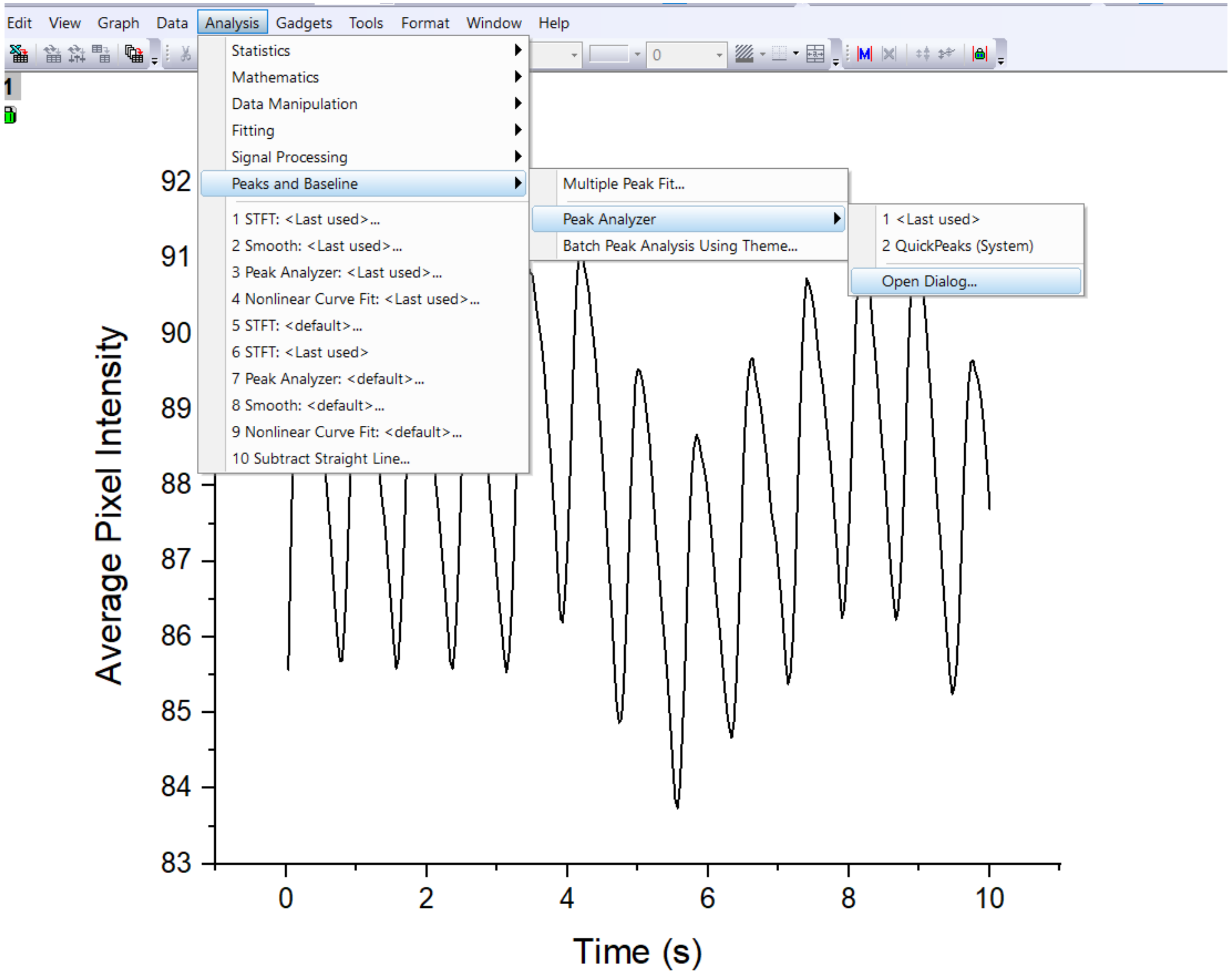

- After select graphic, in tool box, use Analysis -> Peaks and Baseline -> Peak Analyzer -> Open Dialog (for new setup) or Last used (for previous setup)

Setting up Peak Analyzer

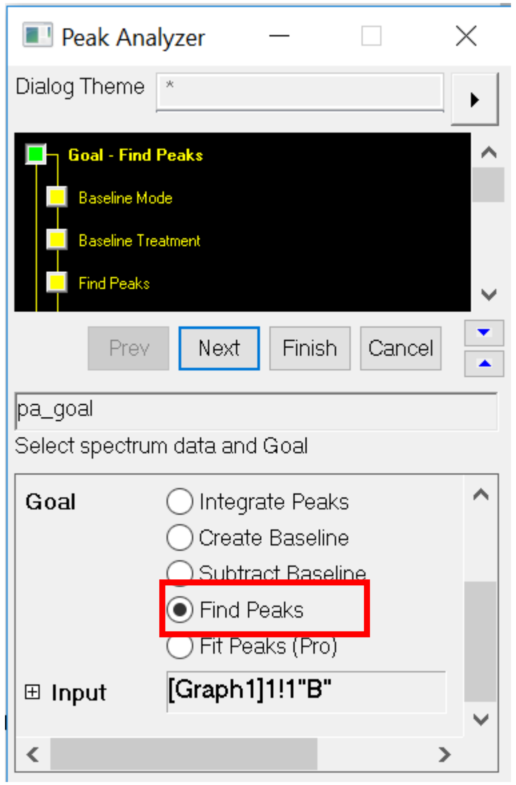

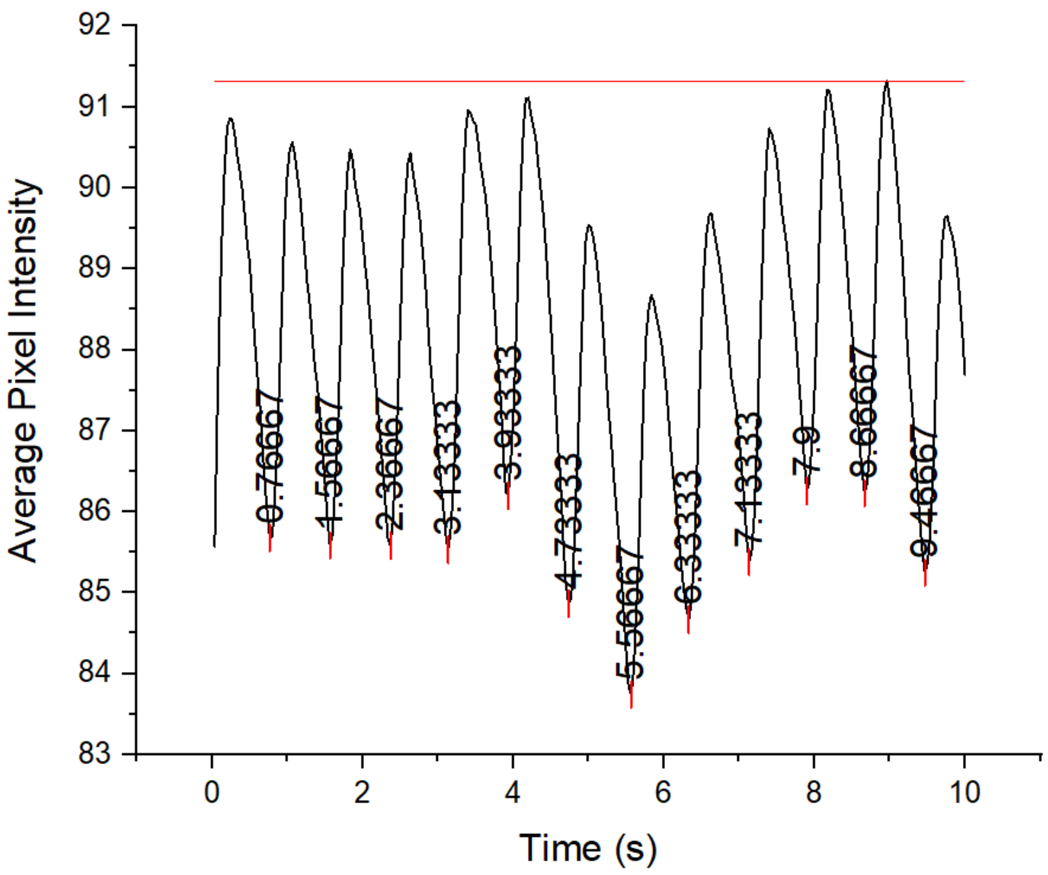

- To obtain time and intensity of each peak, we use "Find Peaks" function

Copy the Data for Further Analysis

Book1 *

	A(X)	B(Y)
Long Name	Peak Centers	Peak Centers
Units		
Comments	Peak Centers of "Smoothed Y1"	Peak Centers of "Smoothed Y1"
F(x)=		
1	0.76667	85.676
2	1.56667	85.58291
3	2.36667	85.57822
4	3.13333	85.53258
5	3.93333	86.20045
6	4.73333	84.86963
7	5.56667	83.73886
8	6.33333	84.66687
9	7.13333	85.38191
10	7.9	86.25433
11	8.66667	86.22774
12	9.46667	85.2433
13		
14		
15		
16		
17		
18		
19		
20		
21		
22		
23		
24		
25		
26		
27		
28		
29		
30		

Sheet1 | Baseline_Data1 | **Peak_Centers1**

	A	B	C
1	Time	Peak Intensity	
2	0.76667	85.676	
3	1.56667	85.58291	
4	2.36667	85.57822	
5	3.13333	85.53258	
6	3.93333	86.20045	
7	4.73333	84.86963	
8	5.56667	83.73886	
9	6.33333	84.66687	
10	7.13333	85.38191	
11	7.9	86.25433	
12	8.66667	86.22774	
13	9.46667	85.2433	
14			
15			

- Minimize the graphic and open the sheet
- Change the window into Peak_Centers
- Copy X and Y data and paste into Excel.
- X as the peak time and Y as peak intensity

Insert New Column in Excel

- **Insert new column between time and peak to calculate time interval**

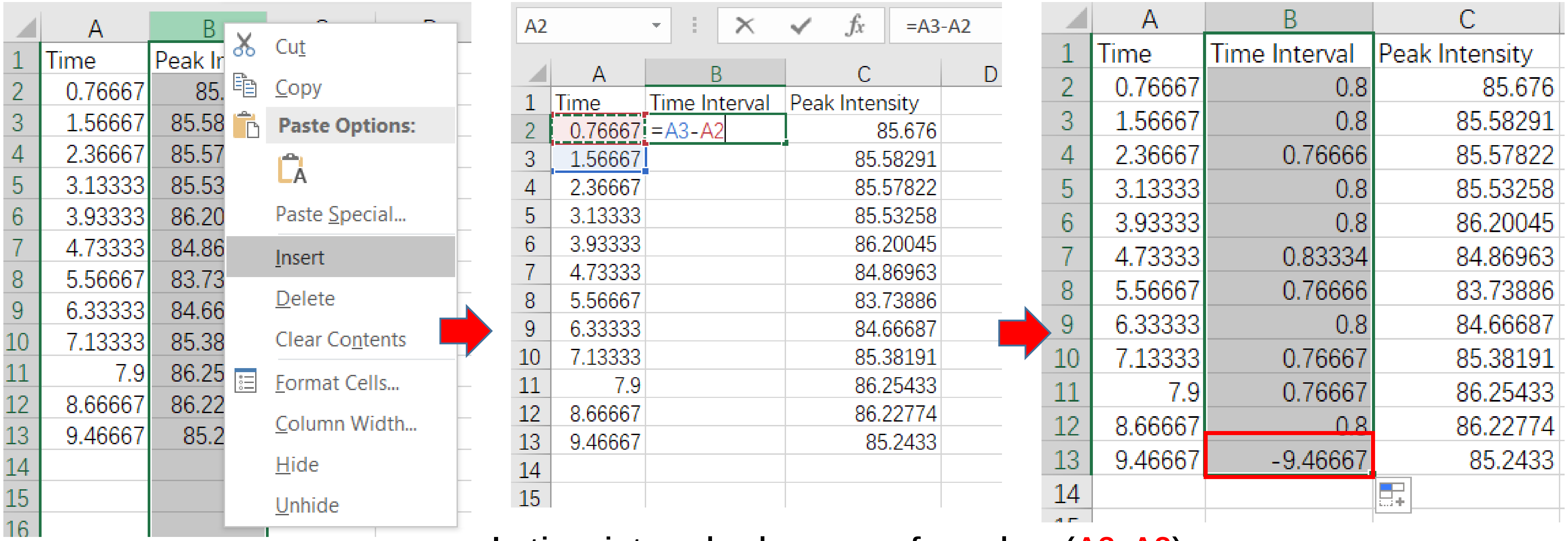

- **In time interval column, use formula: =(A3-A2) or two time point that want to be analysed**
- **Drag down the formula and delete the last calculation as it will show false result**

Calculate Time Interval and Beat per Minute

	A	B	C
1	Time	Time Interval	Peak Intensity
2	0.76667	0.8	85.676
3	1.56667	0.8	85.58291
4	2.36667	0.76666	85.57822
5	3.13333	0.8	85.53258
6	3.93333	0.8	86.20045
7	4.73333	0.83334	84.86963
8	5.56667	0.76666	83.73886
9	6.33333	0.8	84.66687
10	7.13333	0.76667	85.38191
11	7.9	0.76667	86.25433
12	8.66667	0.8	86.22774
13	9.46667		85.2433
14			
15		=average(B2:B12	
16		AVERAGE(**number1**, [number2], …	
17			
18			

	A	B	C
1	Time	Time Interval	Peak Intensity
2	0.76667	0.8	85.676
3	1.56667	0.8	85.58291
4	2.36667	0.76666	85.57822
5	3.13333	0.8	85.53258
6	3.93333	0.8	86.20045
7	4.73333	0.83334	84.86963
8	5.56667	0.76666	83.73886
9	6.33333	0.8	84.66687
10	7.13333	0.76667	85.38191
11	7.9	0.76667	86.25433
12	8.66667	0.8	86.22774
13	9.46667		85.2433
14			
15		0.790909091	
16		=60/B15	
17			

- Calculate average time interval by using the formula: =AVERAGE(B2:B12) or the average of selected time interval
- Calculate the average heart rate (bpm) by using the formula: =60/average time interval

Perform Short-time Fourier transform (STFT) to get heart beat frequency over time changes

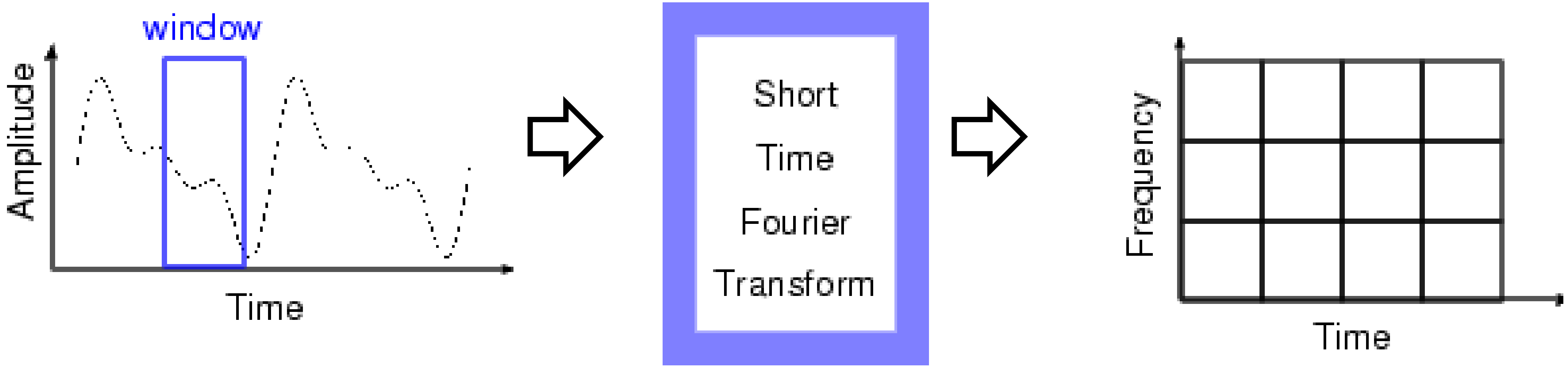

Perform STFT by using Origin 9.1 software

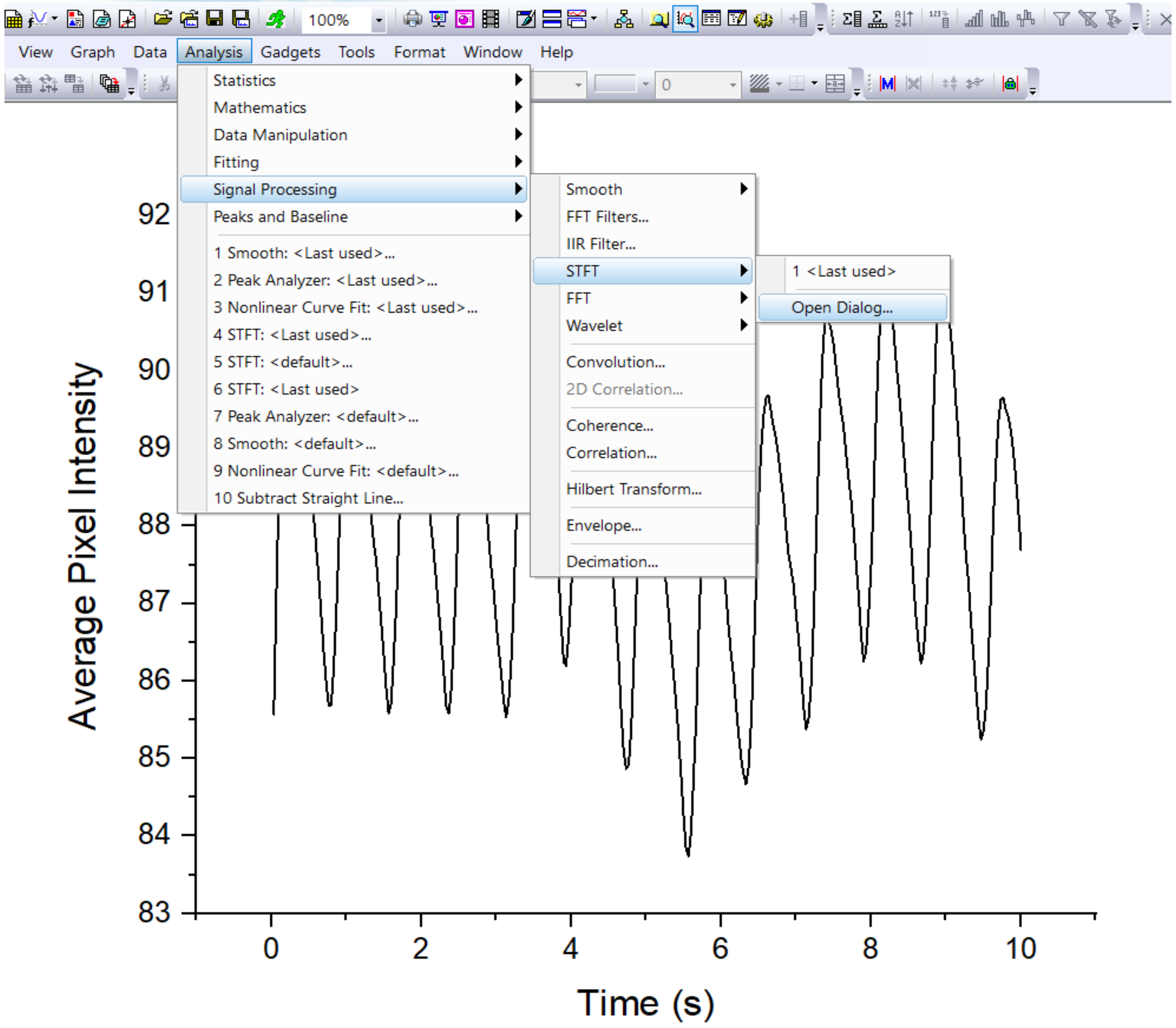

- Click Analysis -> Signal Processing -> STFT -> Open Dialog (for new setup) or <Last used> for previous setup

Perform STFT by using Origin 9.1 software

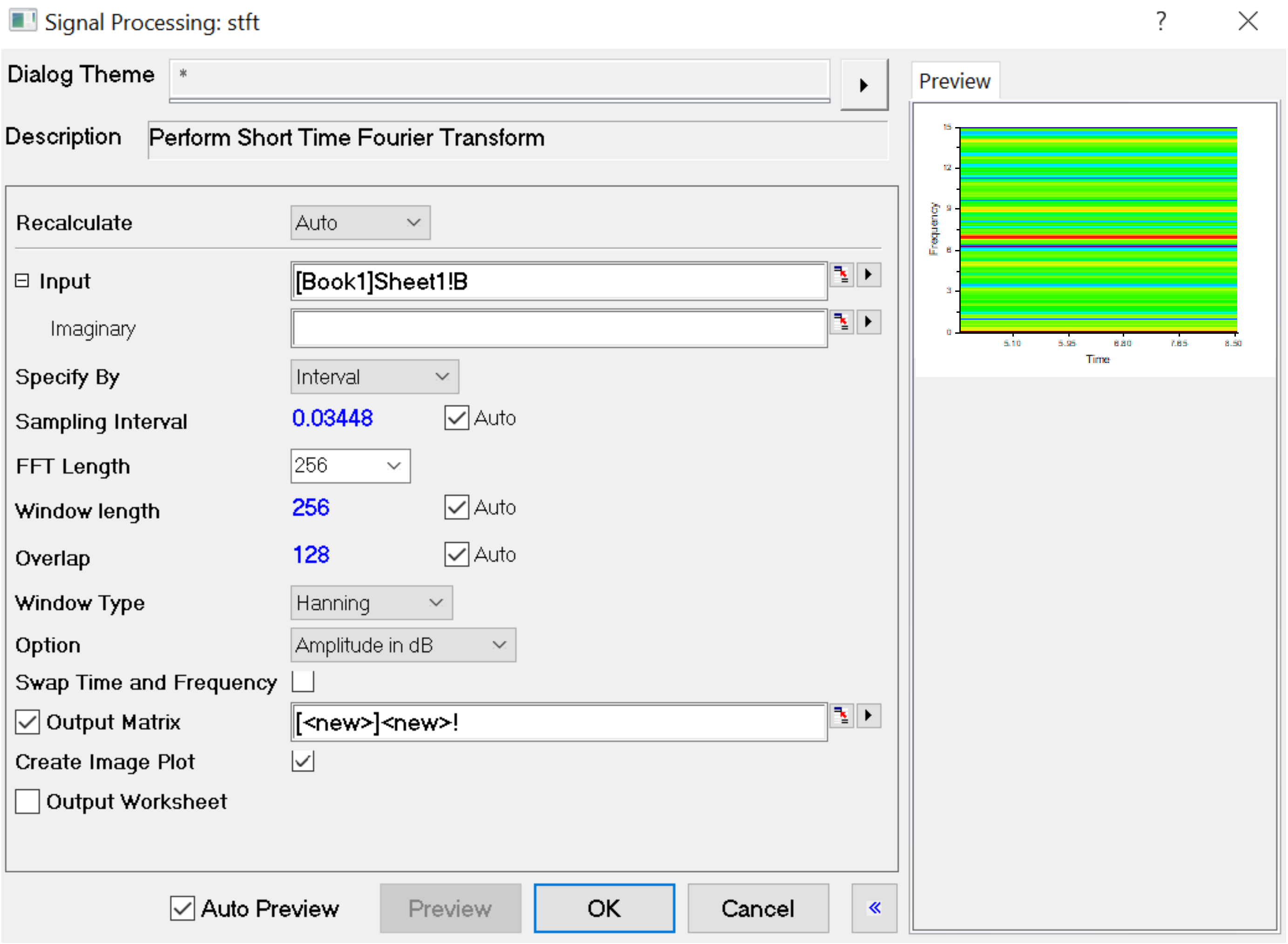

- Change the setup based on the need
- On this experiment, no need to change the parameter (we use the default setting)

Change Scale in STFT Result

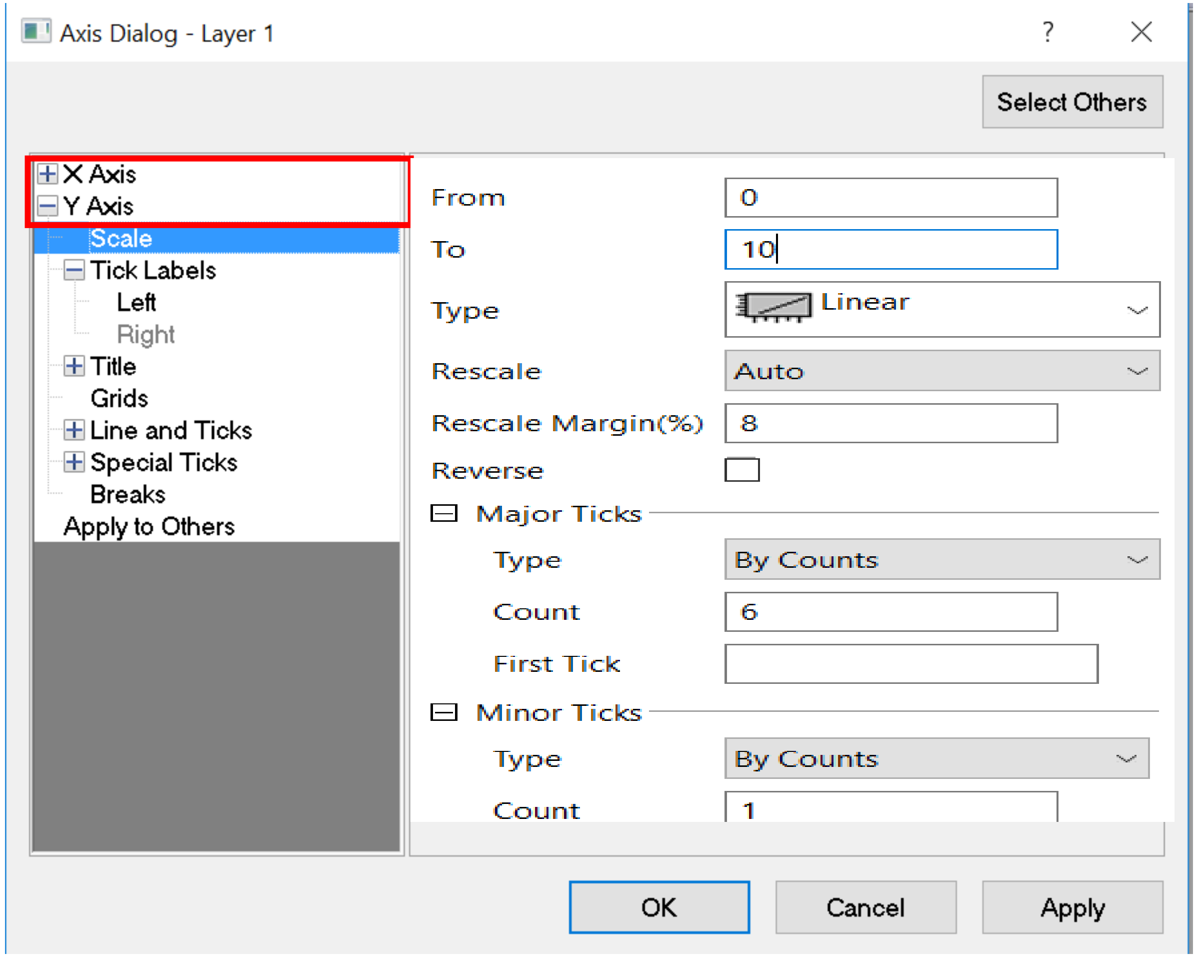

- When the scale range is too wide, it can be adjusted
- To change the X and Y scale double click on the graphic result

Changed Scale STFT Result

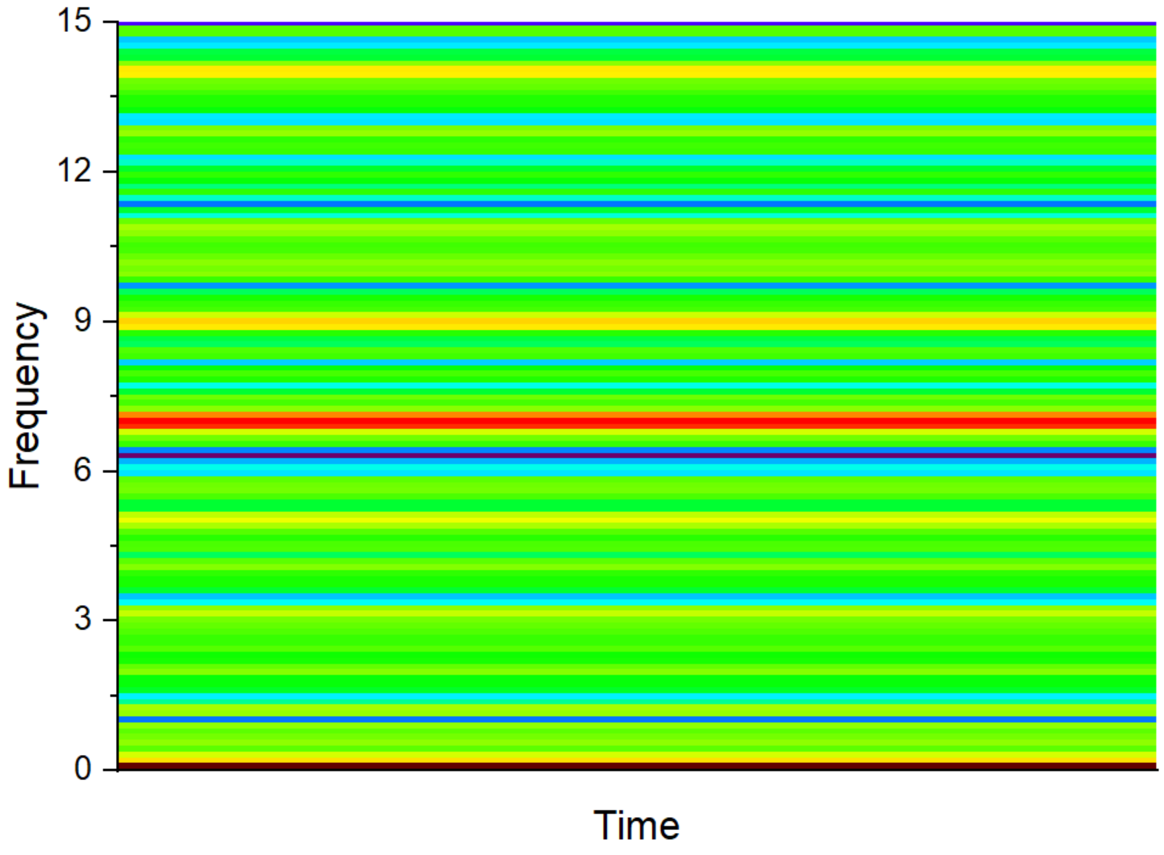

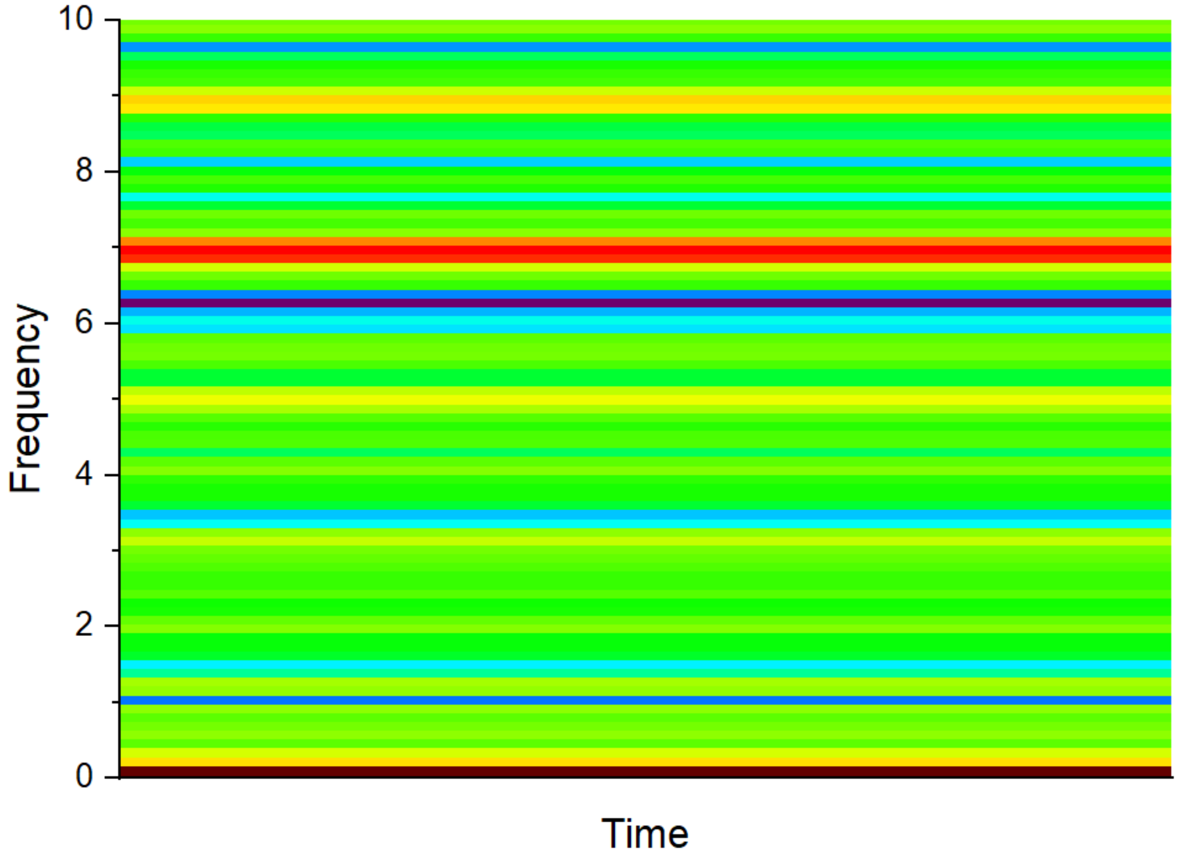

The Daphnia heart beat Frequency = 6.8 – 7.0 Hz = 408-420 bpm

Perform Poincare Plot by using Origin 9.1 software

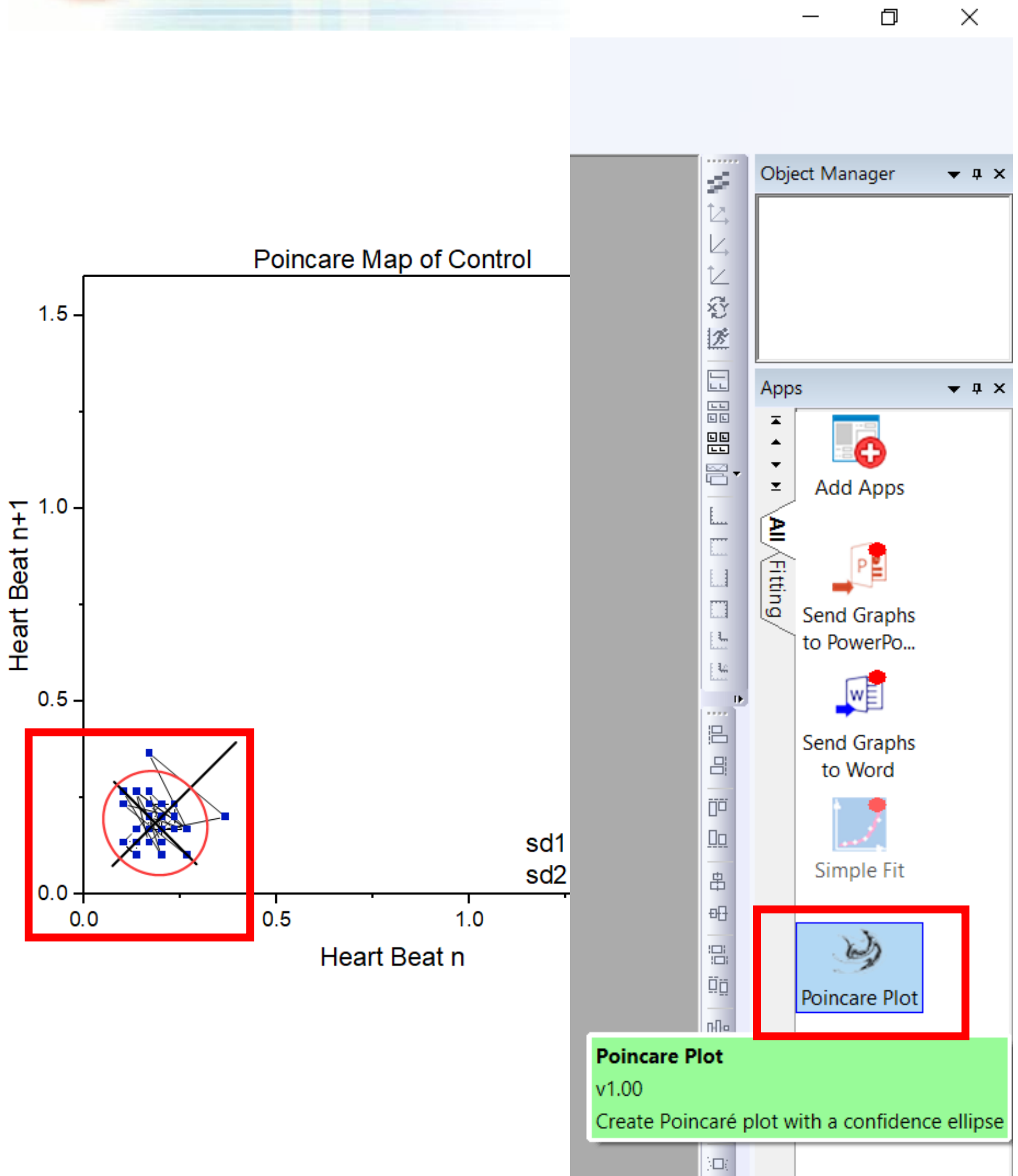

- Poincare Plot, is a scatter graph that visualize the heart rate variability (HRV)
- Poincare Plot plugin can be downloaded from https://www.originlab.com/fileExchange/details.aspx?fid=404
- After download, then the File need to be drag and drop into the Origin Workspace , therefore the icon can be seen on the right side

Perform Poincare Plot by using Origin 9.1 software

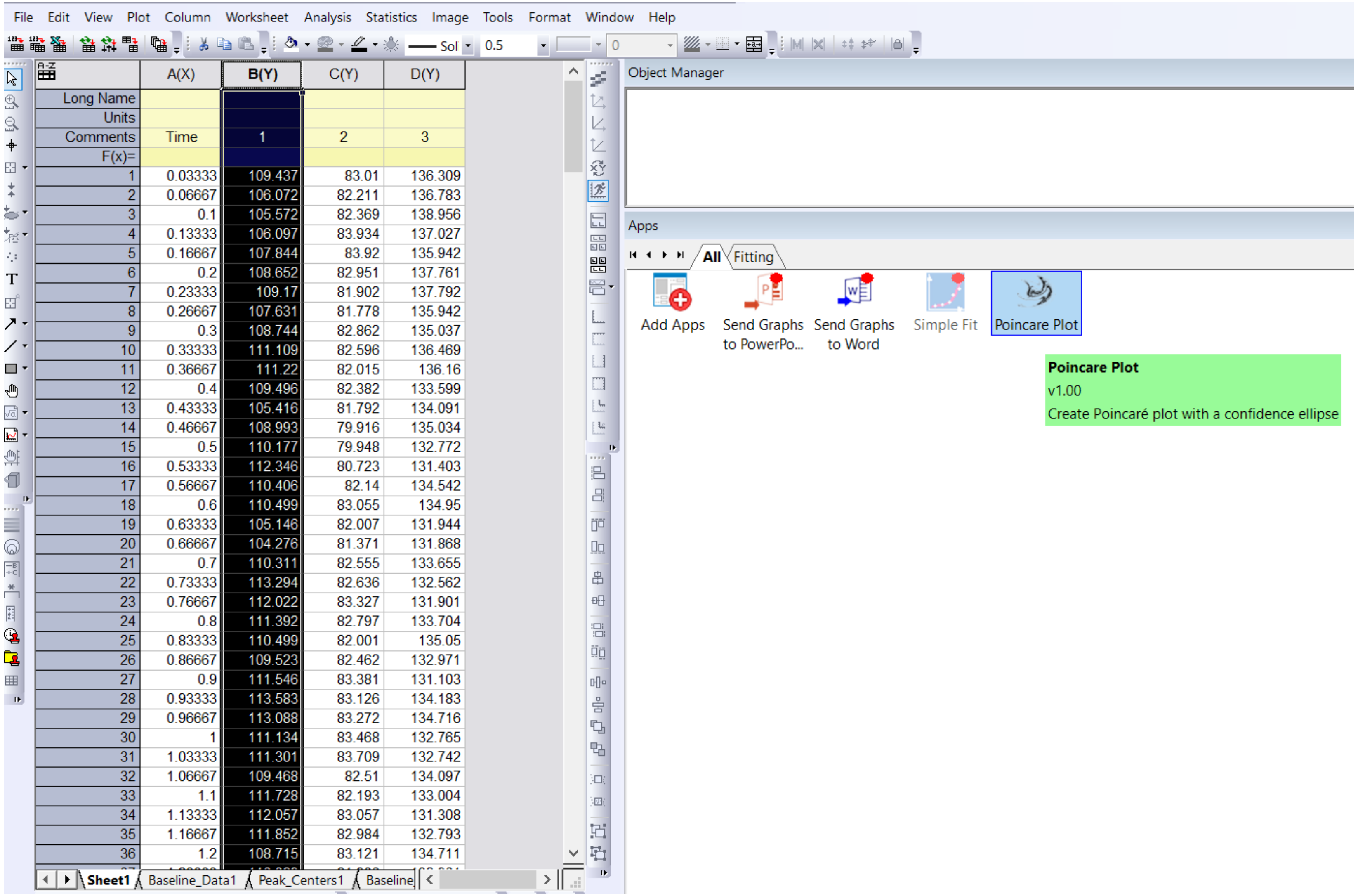

- Copy all the data into Origin 9.1
- Choose 1 column of data and perform Poincare Plot

Perform Poincare Plot by using Origin 9.1 software

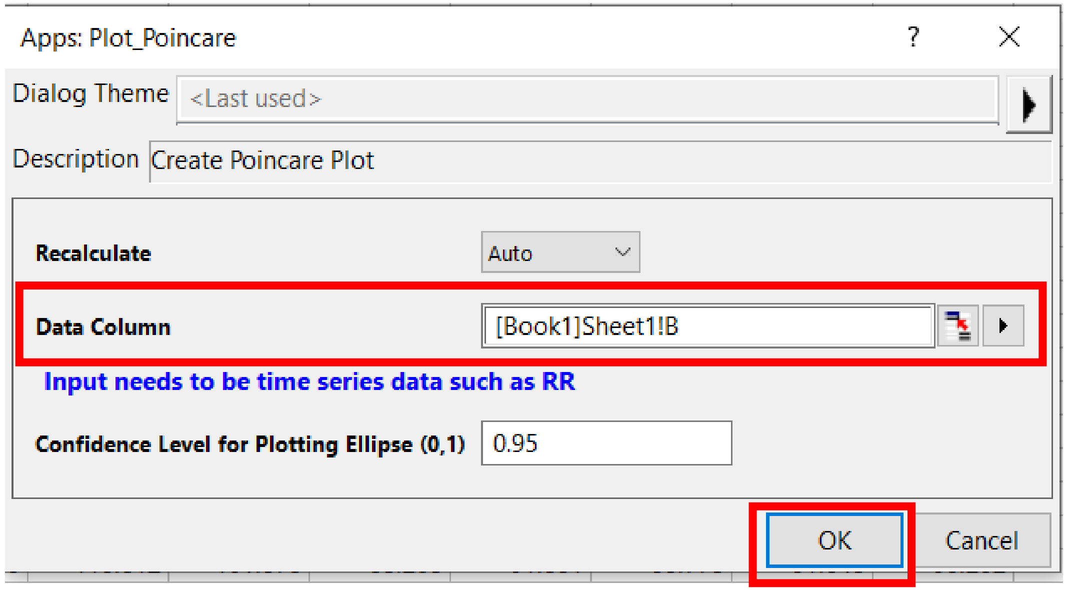

- After performing Poincare plot, it will open a new dialog → make sure the "Data Column" is correct → Press OK

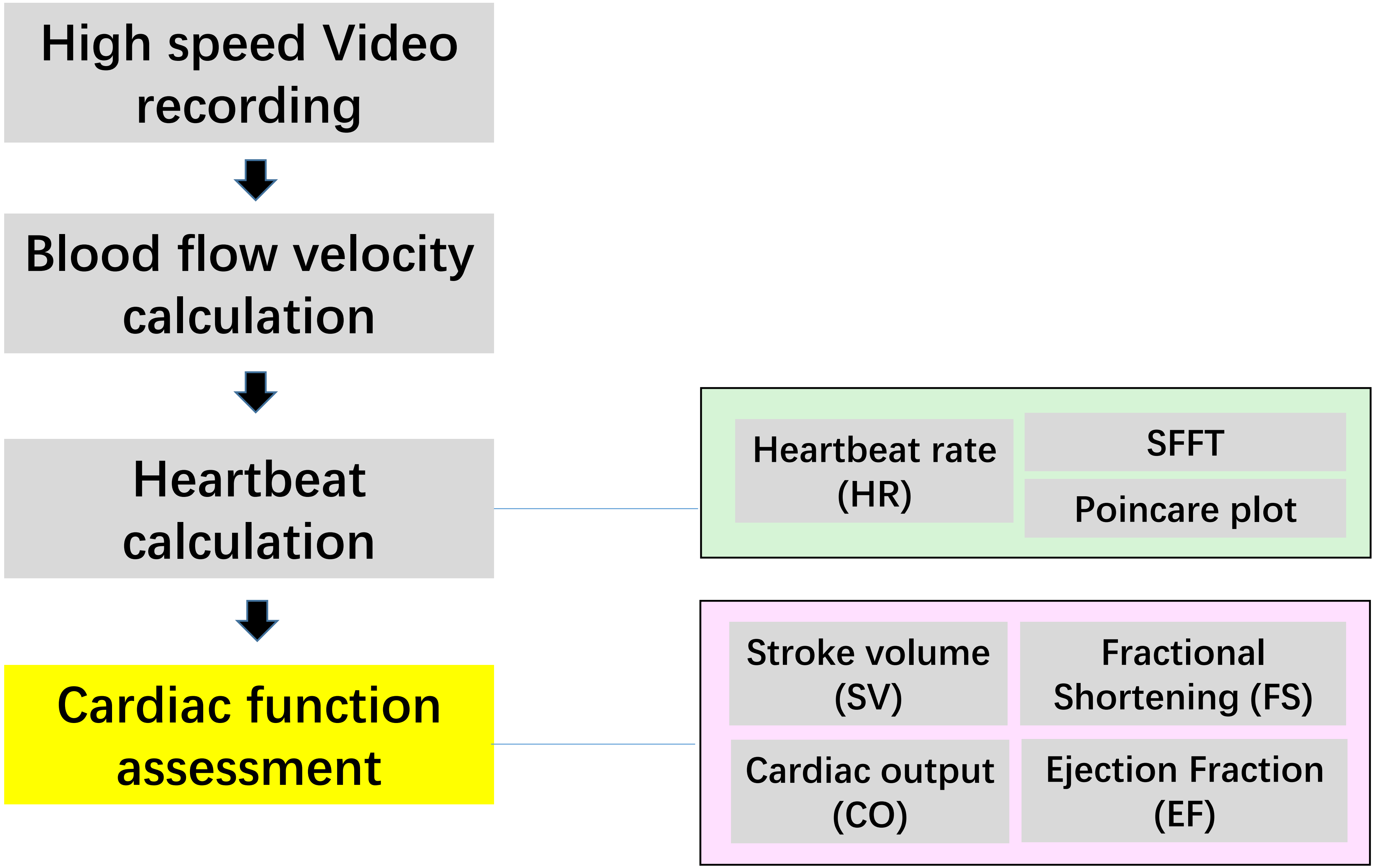
High speed Video recording
Blood flow velocity calculation
Heartbeat calculation
Cardiac function assessment
Heartbeat rate (HR)
SFFT
Poincare plot
Stroke volume (SV)
Fractional Shortening (FS)
Cardiac output (CO)
Ejection Fraction (EF)

CARDIOVASCULAR FUNCTION MEASUREMENT

To measure other parameter of cardiovascular function, we first calculate the diameter of the heart at diastolic and systolic stage, where D_L refers to the length of the long diagonal axis and D_S refers to the length of the short diagonal axis

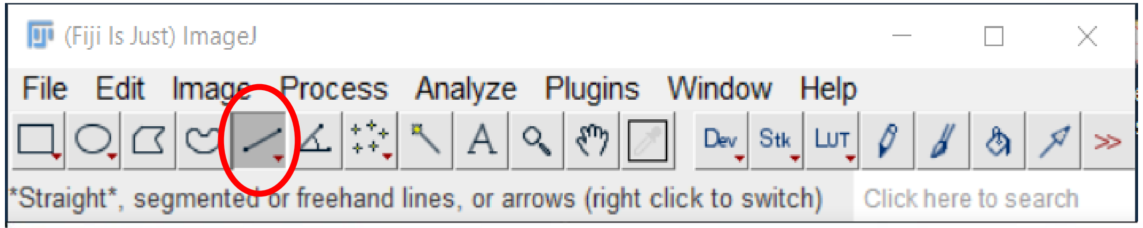

“Straight” line plugin in ImageJ was used to measure the diameter of the heart

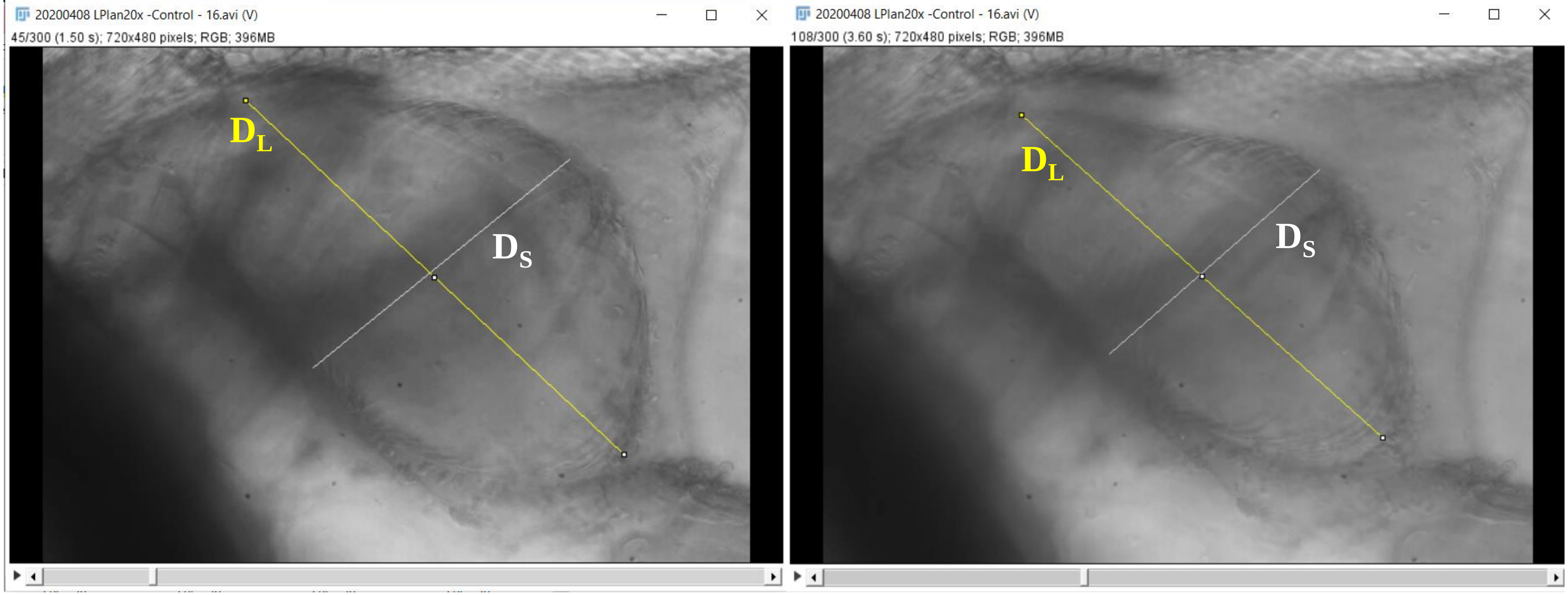

STROKE VOLUME & CARDIAC OUTPUT MEASUREMENT

Stroke volume (SV), the volume ejected in each heartbeat was calculated by subtract the heart end diastolic volume (EDV) and end systolic volume (ESV). (Equation 1). Daphnids was considered to have heart with ellipsoid shape, therefore the heart volume can be measured by using Equation (2)

(Equation 1)

$$SV = EDV - ESV$$

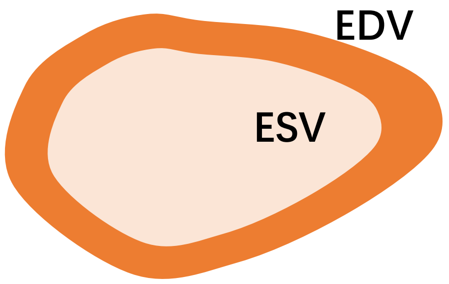

(Equation 2)

$$\text{Volume} = \frac{1}{6} \times \pi \times D_L \times D_S^2$$

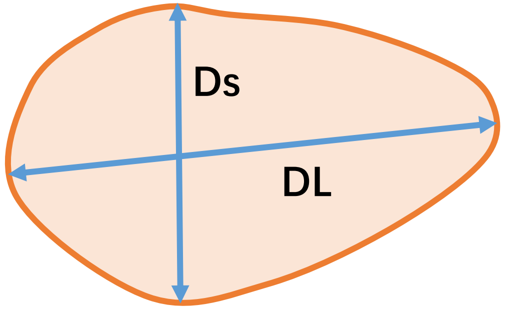

Cardiac output (CO) is the amount of blood the heart pumps in 1 minute. It is determined by multiplying the heart rate by the stroke volume

$$CO = SV \times HR$$

FRACTIONAL SHORTENING & EJECTION FRACTION MEASUREMENT

Fractional shortening (FS) is calculated by measuring the percentage change in heart diameter during diastolic and systolic phase (Equation 3), where D_{SD} refers to the length of the short diagonal axis at diastolic phase and D_{SS} refers to the length of the short diagonal axis at systolic phase.

Ejection Fraction (EF) is the percentage change in end diastolic volume and was calculated by Equation 4

(Equation 3)

$$FS = \frac{D_{SD} - D_{SS}}{Dss} \times 100\%$$

(Equation 4)

$$EF = \frac{SV}{EDV} \times 100\ \%$$

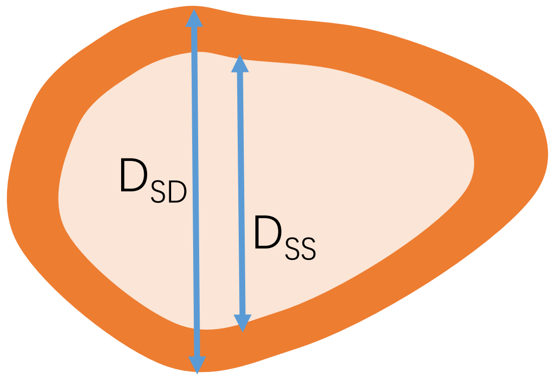

Supplement: Supplementary file 1 [file animals-10-01587-s001.zip › 20200804_Cardiovascular Function Calculation on Daphnids (SOP).pdf]
